# Evolving *Escherichia coli* to use a tRNA with a non-canonical fold as an adaptor of the genetic code

**DOI:** 10.1093/nar/gkae806

**Published:** 2024-09-24

**Authors:** Martin P Edelmann, Sietse Couperus, Emilio Rodríguez-Robles, Julie Rivollier, Tania M Roberts, Sven Panke, Philippe Marlière

**Affiliations:** Department of Biosystems Science and Engineering, Bioprocess Laboratory, ETH Zurich, 4056 Basel, Switzerland; Department of Biosystems Science and Engineering, Bioprocess Laboratory, ETH Zurich, 4056 Basel, Switzerland; Department of Biosystems Science and Engineering, Bioprocess Laboratory, ETH Zurich, 4056 Basel, Switzerland; TESSSI, The European Syndicate of Synthetic Scientists and Industrialists, 75002 Paris, France; Department of Biosystems Science and Engineering, Bioprocess Laboratory, ETH Zurich, 4056 Basel, Switzerland; Department of Biosystems Science and Engineering, Bioprocess Laboratory, ETH Zurich, 4056 Basel, Switzerland; TESSSI, The European Syndicate of Synthetic Scientists and Industrialists, 75002 Paris, France

## Abstract

All known bacterial tRNAs adopt the canonical cloverleaf 2D and L-shaped 3D structures. We aimed to explore whether alternative tRNA structures could be introduced in bacterial translation. To this end, we crafted a vitamin-based genetic system to evolve *Escherichia coli* toward activity of structurally non-canonical tRNAs. The system reliably couples (escape frequency <10^−12^) growth with the activities of a novel orthogonal histidine suppressor tRNA (HisT_UAC_) and of the cognate ARS (HisS) via suppression of a GTA valine codon in the mRNA of an enzyme in thiamine biosynthesis (ThiN). Suppression results in the introduction of an essential histidine and thereby confers thiamine prototrophy. We then replaced HisT_UAC_ in the system with non-canonical suppressor tRNAs and selected for growth. A strain evolved to utilize mini HisT, a tRNA lacking the D-arm, and we identified the responsible mutation in an RNase gene (*pnp*) involved in tRNA degradation. This indicated that HisS, the ribosome, and EF-Tu accept mini HisT *ab initio*, which we confirmed genetically and through *in vitro* translation experiments. Our results reveal a previously unknown flexibility of the bacterial translation machinery for the accepted fold of the adaptor of the genetic code and demonstrate the power of the vitamin-based suppression system.

## Graphical Abstract

**Graphical Abstract:**
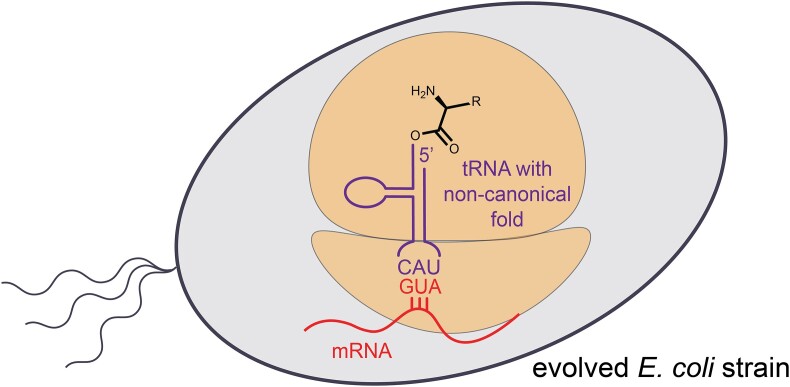

## Introduction

Transfer RNAs (tRNAs) are the adaptor molecules between the genetic code and a protein sequence in all known forms of life. Generally, tRNAs fold into a cloverleaf secondary structure that encompasses the following regions: the acceptor stem, the D-arm (D-stem and loop), the anticodon-arm (anticodon-stem and loop), the variable arm and the T-arm (T-stem and loop). This cloverleaf structure is formed by base pairings in the stem regions and was already suggested when the first tRNA was sequenced in 1965 (1). It was later confirmed by the first crystal structure of a tRNA in 1973, which also revealed the tRNA’s tertiary structure to be L-shaped (2). The L-shape results from interactions between residues in the different regions. One arm of the L is formed by stacking of the D-stem with the anticodon stem and the second arm by stacking of the T-stem with the acceptor stem. Since their initial discovery, the canonical cloverleaf structure in 2D and the L-shaped structure in 3D proved to be highly conserved across all domains of life (3). The L-shaped tertiary structure results in a high stability, is important for recognition by the machineries for tRNA processing and translation, and positions the anticodon and the attached amino acid as required by the ribosome (3).

The variable arm and the D-arm differ in size between different tRNA molecules and in rarer cases, the size of the other arms can also diverge from the standard length (4,5). To this day, the variable size of the arms is the entire extent of structural diversity found in cytosolic tRNAs (6). One example that demonstrates the extent of structural diversity found within cytosolic tRNAs is the unique selenocysteine tRNA. It is characterized by a long variable arm and extended acceptor and D-stems compared to their typical lengths, yet it still adopts the L-fold (7,8). Reports of a bovine mitochondrial tRNA lacking the D-arm and of mitochondrial tRNAs lacking the T-arm in nematodes demonstrated that the canonical L-fold is not an absolute requirement for translation (9,10). These studies described for the first time tRNA molecules that had to adopt non-canonical folds, due to the lack of interactions between the D- and T-arms that are necessary to form the canonical L-fold. More tRNAs that lack some of their arms were discovered, but their occurrence is exclusively restricted to mitochondria, where translation and tRNA processing systems could co-evolve to accommodate these non-canonical tRNAs acquired by a natural drift of the fold (11). It is, to the best of our knowledge, unclear whether a fundamentally different tRNA fold could be utilized by the cytosolic translation machinery and if this is possible, what adaptations would be required in a cell to achieve this.

We turned to an *Escherichia coli* model system to explore whether tRNAs with non-canonical folds could be used in cytosolic translation. It is unlikely that such foreign folds are accepted immediately as adaptors of the genetic code since all known bacteria exclusively use structurally canonical tRNAs. Therefore, we envisioned deploying a genetic system to force a host cell to evolve toward *in vivo* activity of non-canonical tRNAs, and through that process identify critical adaptations. For the development of the selection scheme, we propose the introduction of an alternative genetic code to the host strain through suppression by an orthogonal tRNA (otRNA) minimizing interference with endogenous components (Supplementary Figure S1a). The activity of the orthogonal suppressor tRNA and the cognate aminoacyl tRNA synthetase (ARS) is coupled to the growth of an *E. coli* strain by the expression of a mutated conditionally essential target gene. Due to the mutation, only the target gene product that is synthesized with suppression of the mutated codon is active. In previous work, such suppression systems that couple growth with an alternative genetic code were introduced into *E. coli* using suppression targets involved in either antibiotic resistance, primary carbon metabolism or the biosynthesis of DNA building blocks (12–14). Here, we propose to use an enzyme that is critical for the biosynthesis of a vitamin. We hypothesize that this approach is advantageous due to the fewer active enzyme molecules required for growth, based on the low number of vitamin molecules sufficient per cell in selective (vitamin-free) medium. Consequently, only a few suppression events per cell would be required for the production of sufficient vitamin molecules to confer prototrophy, rendering a vitamin-based system highly sensitive to suppressor activity.

For the application of the system, the otRNA is replaced by tRNAs with non-canonical folds (Supplementary Figure S1b). We reasoned that if the growth dependency on the alternative genetic code introduced by the system is absolute, cells must adapt during selection to accept the novel tRNA fold in translation in order to grow. A potential drawback of using a vitamin-based target in a suppression system for evolution experiments is that it might be escaped frequently, since only a few vitamin molecules are sufficient for growth. Indeed, the rapid emergence of escapees has been previously reported in strains from which genes involved in vitamin biosynthesis had been deleted (15,16). The lacking catalytic activity could be (poorly) exhibited by an alternative enzyme or the vitamin could be produced by an emerging alternative pathway. For the envisioned suppression system, this would render the selection step and thereby the system itself obsolete. In principle, the higher the sensitivity of a suppression system, the higher the risk of such a metabolic escape. In fact, the required flux through any alternative pathway sufficient for growth should be inversely proportional to the sensitivity of the selection system. To avoid metabolic escape, the choice of the suppression target and the reaction that is catalyzed by its gene product, is of utmost importance. Ideally, there should be no way for the cell (using its existing genetic makeup or by evolution) to substitute the function of the suppression target gene product or to bypass the target by producing a metabolite in the vitamin biosynthesis pathway downstream of the target step.

In this work, we present a thiamine-based suppression system that complies with the aforementioned requirements. The system couples the activity of a novel orthogonal histidine suppressor tRNA and the cognate histidine ARS with the growth of a thiamine auxotrophic *E. coli* strain through the missense suppression-dependent synthesis of active ThiN. ThiN catalyzes the last step in thiamine biosynthesis and was rationally selected to minimize the chance that metabolic escape occurs. The resulting ThiN-based system is highly sensitive to suppressor activity and highly secure against metabolic escape with an escape frequency of <10^−12^. We then exploited the system to explore whether the supposedly immutable fold of bacterial tRNAs can be fundamentally altered. We successfully evolved *E. coli* to utilize a tRNA lacking the entire D-arm in translation. Through genetic experiments, we pinpointed the adaptation responsible for the obtained phenotype to a single point mutation in an RNase gene involved in tRNA degradation. Therefore, the entire required translation machinery is capable, *ab initio*, of accepting the foreign and artificial tRNA fold without any adaptations. We confirmed this result by successfully deploying the purified D-arm-less tRNA for *in vitro* aminoacylation and *in vitro* translation. In conclusion, our study harnesses the power of the vitamin-based suppression system to reveal a hitherto unobserved flexibility of the bacterial translation machinery for the accepted fold of the adaptor of the genetic code.

## Materials and methods

### Media

LB (Miller Broth) medium (10 g l^−1^ tryptone, 5 g l^−1^ yeast extract, 10 g l^−1^ NaCl) was used for strain maintenance, cloning and transformation procedures, strain construction and cultures for RNA or DNA extraction. TB medium (12 g l^−1^ tryptone, 24 g l^−1^ yeast extract, 4 ml l^−1^ glycerol, 0.74 mM K_2_HPO_4_, 0.26 mM KH_2_PO_4_) was used for protein production for protein purification. M9 medium (48 mM Na_2_HPO_4_, 22 mM KH_2_PO_4_, 9 mM NaCl, 19 mM NH_4_Cl, 2 mM MgSO_4_, 0.13 mM CaCl_2_, 7.6 μM MnCl_2_, 6.5 μM ZnSO_4_, 2.3 μM Na_2_EDTA, 4.9 μM H_3_BO_3_, 0.9 μM CuCl_2_, 1 μM Na_2_MoO_4,_ 17.5 μM FeSO_4_, pH 7.4 adjusted with NaOH) supplemented with 0.5 % (w/v) glucose as carbon source served as the initial selective medium and was termed M9(glu). For optimization of the selective medium 2 μM calcium pantothenate (pan), 1 μM pyridoxal hydrochloride, 1 mM shikimic acid, 0.5 mM isoprenol, 0.5 mM valine/leucine/isoleucine (ILV, 0.5 mM each), 1 mM succinic acid (suc) and 10 mM potassium acetate were supplemented individually or in combinations to M9(glu). The final selective medium M9(glu)^sup^ consisted of M9(glu) medium supplemented with 2 μM pan, 0.5 mM ILV and 1 mM suc. For nonselective M9(glu) or M9(glu)^sup^ medium, 10 μM thiamine hydrochloride was added to cultures. Agar plates contained 1.5 % (w/v) agar in the respective medium.

All suitable antibiotics for plasmid retention were always added to M9(glu), M9(glu)^sup^, LB and TB media (agar plates and liquid cultures) if strains harbored one or multiple plasmids, unless otherwise specified. The following concentrations were used depending on the plasmid-conferred resistance: chloramphenicol (cm, 34 μg ml^−1^); kanamycin (kan, 50 μg ml^−1^) and carbenicillin (carb, 100 μg ml^−1^). Additionally, spectinomycin (spec, 50 μg ml^−1^) or apramycin (apr, 50 μg ml^−1^) were added to liquid media or agar plates for cultivation of strains Sel_aux_, Sel^0^_hit_ and Sel_hit_, which contained a genomic resistance cassette, irrespective of whether they harbored a plasmid or not (Supplementary Table S1). The relevant antibiotics, as described above, were always applied unless explicitly stated differently.

### Cultivation and transformation of *E. coli* strains

Cultivation in LB and TB medium or on LB or TB agar plates was performed at 37°C. All cultivations in M9(glu) or M9(glu)^sup^ medium and on M9(glu) or M9(glu)^sup^ agar plates were performed at 30°C. Liquid cultures were agitated at 200 rpm in an ISF1-X shaking incubator (Kuhner), unless otherwise specified. Optical density measurements at 600 nm (OD_600_) were performed using a D30 spectrophotometer (Eppendorf), if not indicated otherwise. Strains were stored as cryo stocks containing 9 % (v/v) DMSO, prepared from an LB overnight culture, or on LB plates at 4°C for short term storage.

Unless noted otherwise, electrocompetent cells were prepared as described in the following. A 3 ml LB preculture was inoculated from an individual clone of the respective *E. coli* strain on a LB agar plate and grown overnight. The next day, a new 100 ml LB culture was inoculated from the preculture by a 1:100 dilution and grown to an OD_600_ between 0.6 and 1. The cells were chilled on ice for 10 min and maintained cooled from here on. An aliquot of 50 ml of cells was centrifuged (4000 *g*, 10 min, 4°C), the supernatant was discarded and the pellet was resuspended in 20 ml of ice-cold water. This process was repeated two times. After a final fourth centrifugation step, the pellet obtained was resuspended in 1 ml ice-cold water containing 9 % (v/v) DMSO. Aliquots of 50 μl of the competent cells were frozen in liquid nitrogen and stored at −80°C.

Transformations were carried out via electroporation. An aliquot of 1 μl of 20 ng μl^−1^ plasmid DNA in water (1 μl per plasmid in a co-transformation) was mixed with one aliquot of competent cells thawed on ice. The electroporation was performed with a 1 mm gap-width cuvette (Cell projects limited) at 1.8 kV using a MicroPulser (BioRad). After that, 1 ml of LB medium without any antibiotic was added immediately. Cells were recovered for 1 h at 37°C at 1000 rpm in a ThermoMixer C (Eppendorf), unless otherwise specified. Cells were then plated on LB agar plates containing appropriate antibiotics and incubated overnight.

### Growth experiments

All experiments to investigate growth on selective medium were performed at 30°C. For experiments on selective agar plates, individual colonies on LB plates were transferred to selective medium plates as follows: An individual colony was touched with an inoculation loop. Cells were then transferred into 400–800 μl of 1× Dulbecco’s Phosphate Buffered Saline (PBS) (Merck). The suspension was vortexed thoroughly and an aliquot of 5 μl was streaked out on the selective plate. Close adherence to this was crucial to consistently avoid false positive results arising from carry-over from nonselective to selective plates.

For growth experiments in selective liquid medium, precultures were prepared in the medium used for the actual experiments afterwards, supplemented with 10 μM thiamine. Precultures were inoculated from individual colonies on LB agar plates and grown overnight. An aliquot of 1 ml of a fully grown preculture was centrifuged (4000 *g *, 4 min) and resuspended in 1 ml of the medium used for the respective growth experiment or in PBS. This washing procedure was repeated three times. Growth curves were recorded in either an Infinite 200 PRO or an Infinite M Nano plate reader (Tecan) with a culture volume of 200 μl per well in Nunc MicroWell 96-well plates (Thermo Fisher Scientific). During experiments, the plates were agitated at 432 rpm with a 1 mm amplitude in orbital mode. The OD_600_ was measured periodically every 30 or 45 min in the device. The initial starting OD_600_ for growth experiments in liquid medium was 7 × 10^−5^. We used this initial OD_600_ to avoid false positive results. The thiamine auxotrophic strain Sel_aux_ is capable to perform ∼9.5 doublings in M9(glu)^sup^ after the aforementioned washing procedure, presumably due to intracellularly stored thiamine (Supplementary Figure S2). Taking these doublings into consideration, the OD_600_ resulting from our initial inoculation, when all thiamine from prior sources has been depleted, is 0.05. A similar behavior was shown in a previous study for other vitamins (17). Growth assays were performed in three replicates, if not indicated differently. For all presented data, the background OD_600_ (medium without cells) was subtracted.

### Adaptive evolution experiments

For adaptive evolution experiments, precultures were prepared and cells washed as described previously for growth experiments. For adaptive evolution experiments in liquid cultures, selective cultures were inoculated with the washed cells for a starting OD_600_ of 0.0007, targeting an OD_600_ of 0.5 after 9.5 initial doublings, unless otherwise specified. The OD_600_ was monitored over the course of the experiment. Alternatively, for adaptive evolution experiments on agar plates, 20 μl of the washed cells with an OD_600_ of 0.1 were streaked on selective agar plates, if not indicated otherwise. This would result in 1.5 × 10^9^ cells after 9.5 initial doublings (on M9(glu)^sup^ agar plates) under the assumption that the same number of doublings take place as in liquid medium. Plates and the OD_600_ of cultures were continuously inspected over the course of long-term adaptive evolution experiments. In experiments performed to investigate the emergence of metabolic escapees and to measure escape frequencies for systems lacking a component, the appearance of a colony or a substantial increase of the OD_600_ was considered an escape event. Escape frequencies on agar were calculated as the number of colonies per total cells present after 9.5 initial doublings of the plated cells. Escape frequencies in liquid media could only be determined as upper limits if no escape was observed during the adaptive evolution experiment. For the suppression system lacking *hisT_TAC_* no metabolic escape was detected. Therefore, we considered colonies observed in adaptive evolution experiments with systems containing non-canonical tRNA designs as potential selection hits.

### Plasmid construction

Plasmids, if constructed for this work, were either ordered from Twist Biosciences or constructed in house (Supplementary Table S2). Selected plasmid maps and sequences are shown in Supplementary Figure S3 and Supplementary sequences.

For plasmids acquired from Twist Biosciences, synthetic DNA fragments were synthesized and cloned by the manufacturer into custom vectors provided by us to the manufacturer (using the ‘clonal gene synthesis’ platform). The obtained plasmids were used to transform a cloning strain (Supplementary Table S1). Plasmid DNA was purified from an overnight LB culture, inoculated from an individual clone, using the QIAprep Spin Miniprep kit (Qiagen) as per the manufacturer’s instructions. The correct sequence of the cloned fragment was verified by us through Sanger sequencing (Microsynth AG).

For plasmids we constructed, Gibson assembly and standard molecular biology workflows were used (18). Briefly, plasmid vectors were amplified and linearized by PCR using the Q5 High Fidelity 2x Master Mix (New England Biolabs) according to the manufacturer’s protocol. Polymerase chain reactions (PCRs) were carried out in a peqSTAR 2x (VWR) or in a nexus GX2 (Eppendorf) thermocycler. Inserts were synthesized as double-stranded DNA fragments (‘gblocks’, Integrated DNA Technologies) and used directly for Gibson assembly. Alternatively, inserts were obtained by amplification of the target sequence via PCR using the Q5 High Fidelity 2x Master Mix (New England Biolabs) according to the manufacturer’s protocol. An aliquot of 1 μl DpnI (20 U/μl, New England Biolabs) was added directly to completed PCR reactions and the samples were incubated for 2–3 h at 37°C. Subsequently, DNA fragments were separated by standard agarose gel electrophoresis. Target gel bands were excised, and the DNA was extracted using the Zymoclean Gel DNA recovery kit (Zymo Research) following the manufacturer’s guidelines. The DNA was further purified using the DNA Clean and Concentrator-5 kit (Zymo Research) according to the manufacturer’s protocol. Gibson assembly was performed using a reported one-step isothermal DNA assembly protocol with an incubation time of 60 min and 20 base pair overhangs (18). The required overhangs were introduced into the linearized vector or the insert by PCR (overhangs sequence present in primer) or were already present in the acquired gblock. An *E. coli* cloning strain was transformed with appropriate amounts of the Gibson assembly reaction (∼5 μl). If necessary, the DNA in the Gibson assembly reaction samples was further purified by using the DNA Clean and Concentrator-5 kit (Zymo Research) as per the manufacturer’s instructions. Plasmid DNA was purified from an LB overnight culture, inoculated from an individual clone, using the QIAprep Spin Miniprep kit (Qiagen) according to the manufacturer’s protocol. The correct sequence of the cloned fragment was verified by Sanger sequencing.

The *thiN* domain of *Thermotoga maritima* MSB8 *thiDN* (UniProt entry Q9WZP7) was defined as bases 586–1194 of *thiDN* (corresponding to amino acids 196–398) (19). For all plasmids constructed for this work containing *thiN* or *thiN* variants, a gene codon optimized for *E. coli* was used. All *thiN* variants, in which the codon for H95 was mutated to differ in all three bases to any histidine codon, were further modified. In these genes all other occurrences of that new codon were replaced by synonymous codons to avoid having multiple suppression codons in the *thiN* variant.

### Plasmid curing

To cure plasmids from a strain, a 3 ml LB culture was inoculated from an individual colony of that strain. Antibiotics, whose resistance genes were encoded on the plasmids targeted for curing, were omitted from this culture. The culture was grown overnight at 42°C. The next day, a new culture using the same medium was inoculated from the initial one (1:2000 dilution). This procedure was repeated for multiple days. From the third day on, an aliquot of 20 μl of each grown culture was plated on LB agar without antibiotics that served as selection marker for the plasmids and the resulting plate was incubated at 37°C overnight. The next day, individual colonies were selected from this plate and resuspended in 100 μl PBS. Aliquots of 20 μl of it were streaked out on LB plates that contained either none or one of the antibiotics that served as selection markers for the plasmids. These plates were incubated at 37°C overnight. The absence of growth on all plates containing the antibiotics was indicative of successful plasmid curing. In this case, a single colony from the LB plate without antibiotics was selected to inoculate an LB overnight culture, from which a cryo stock was prepared.

### Whole genome sequencing

To prepare the samples for whole genome sequencing (WGS), an LB culture was inoculated from an individual colony of a strain and grown overnight. The following day, the DNA was extracted using the GeneElute Bacterial Genomic DNA Kit (Merck) according to the manufacturer’s protocol but with the DNA elution performed with water. The extracted DNA was stored at 4°C.

The genome of Sel_aux_ was sequenced by long-read PacBio sequencing and assembled *de novo* to serve as a reference genome. The library preparation, sequencing and genome assembly were performed by the Functional Genomics Center Zurich as described below. A SMRTbell library was produced using the SMRTbell Express Template Prep Kit 2.0 (Pacific Biosciences). The input concentration was measured using a Qubit Fluorometer dsDNA High Sensitivity assay (Life Technologies). To assess the fragment size distribution, the samples were run on a Femto Pulse Device (Agilent). Approximately 1 μg of gDNA was mechanically sheared to an average size distribution of 10 kb using a g-TUBE (Covaris). Approximately 500 ng of sheared gDNA were DNA damage repaired and end-repaired using polishing enzymes. A ligation was performed to create the SMRTbell template according to the manufacturer’s instructions. The individual samples were barcoded during the library preparation using Barcoded Overhang Adapter Kit - 8A and 8B (Pacific Biosciences). The final libraries were pooled and size-selected around 3 kb using AMPure PB Beads (Pacific Biosciences). Final quality control and quantification were performed using a Femto Pulse Device (Agilent) and a Qubit Fluorometer dsDNA High Sensitivity assay (Life Technologies). A ready to sequence SMRTbell-Polymerase Complex was created using the Sequel II Binding Kit 2.0 and Internal Control 1.0 (Pacific Biosciences) according to the manufacturer’s instructions. The Pacific Biosciences Sequel II instrument was programmed to sequence the libraries on one Sequel II SMRT Cell 8M (Pacific Biosciences) in Continuous Long Reads (CLR) mode, with a movie collection time of 15 h, using the Sequel II Sequencing Kit 2.0 (Pacific Biosciences). The *de novo* assembly of the genome was performed using the ‘Microbial Assembly Application’ in the PacBio software suite SMRT Link v8.0 (Pacific Biosciences) with default parameters. The obtained genome was manually curated to remove PacBio sequencing errors.

For all other strains library preparation and WGS were performed by Novogen Co., Ltd. Samples were paired-end sequenced using the Illumina NovaSeq 6000 platform (insert size 350 bp, read length 150 bp). The obtained reads were mapped to the reference genome in the Geneious Prime 2022 software using the Mauve plugin (20).

### Strain construction

#### Strain Sel_aux_*

To introduce the *pnp* point mutation (resulting in *pnp*:G573S variant) in a targeted manner into Sel_aux_, we followed a two-step procedure. First, genomically integrated antibiotic resistance (spec and apr) cassettes were removed from Sel_aux_ via Flp-catalyzed excision to yield strain Sel_aux_* (21). In second step the desired *pnp* point mutation was introduced into Sel_aux_* by λ-red recombineering yielding strain Sel_aux_pnp-mut_.

Sel_aux_* is identical to Sel_aux_ expect that it lacks the spec and apr resistance cassettes. To obtain Sel_aux_*, strain Sel_aux_ was transformed with the pCP20 plasmid, which contains the *flp* flipase gene. The LB agar plate containing carb with transformed cells was incubated at 30°C overnight. Individual colonies were selected and transferred to another LB plate containing carb, which was incubated at 37°C overnight. This procedure was repeated one more time. On the following day individual colonies were resuspended in 100 μl of PBS, vortexed and 20 μl of each suspension were plated on separate LB plates containing one of the antibiotics spec, apr and carb or none at all. After a 30°C overnight incubation, a strain that only grew on the antibiotic-free plate (indicating loss of the resistance cassettes as well as pCP20) was selected. An LB overnight culture was inoculated from an individual colony and grown at 37°C overnight to prepare a cryo stock. The successful excision of the antibiotic cassettes was confirmed by colony PCRs and Sanger sequencings of the respective loci. For this, a colony of the selected strain was touched with an inoculation loop. The cells collected with the loop were directly transferred into tubes for PCR reactions. Colony PCRs were performed with the Q5 High Fidelity 2x Master Mix (New England Biolabs), with a modified initial denaturation step at 98°C for 5 min, according to the manufacturer’s guidelines using the cells instead of a DNA template. The resulting DNA fragments were separated by standard agarose gel electrophoresis, target bands at the expected height were excised and the DNA was extracted using the Zymoclean Gel DNA recovery kit (Zymo Research) according to the manufacturer’s protocol. The correct genomic edits were verified by Sanger sequencing of the extracted DNA. The resulting strain, devoid of antibiotic resistance cassettes and pCP20, was named Sel_aux_*. WGS of Sel_aux_* confirmed the removal of the antibiotic cassettes. No additional mutations compared to the progenitor and reference strain Sel_aux_ were observed.

#### Strain Sel_aux_pnp-mut_

In a second step, the *pnp* point mutation was introduced into Sel_aux_* by λ-red recombineering (22). Sel_aux_* was co-transformed with pHisS_MiniHisT_TAC_, pThiN(H95V_GTA_) and pKD46-kan. The agar plate with transformed cells was incubated at 30°C overnight. An individual colony was used to inoculate a 20 ml LB culture. The culture was incubated at 30°C overnight without shaking. Approximately 16 h later, when an OD_600_ of 0.6 was reached, arabinose was added to 0.15 % (w/v) to induce expression of the λ-red genes from pKD46-kan. The culture was incubated further for 2 h at 30°C and 1000 rpm. Then, the cells of culture were made electrocompetent as described before but with the volume of the water used for washing adjusted to 8 ml. The final cell pellet was resuspended in 200 μl of water. An aliquot of 50 μl of competent cells was electroporated with 0.5 μl of a 100 μM solution of the oligonucleotide mMPE007 (Supplementary Table S3). The first 5 bases of the 5′-end in mMPE007 were connected via a phosphorothioate linkage to increase recombination efficiency (23). The oligonucleotide was designed using the MODEST web-based design tool (http://modest.biosustain.dtu.dk). Post electroporation, cells were recovered for 2–3 h at 30°C and 1000 rpm, centrifuged (4000 *g*, 4 min) and the resulting pellet was resuspended in 1 ml of M9(glu)^sup^. The washing process was repeated three more times. Afterward, 20 μl of washed cells at an OD_600_ 0.05 were plated on a M9(glu)^sup^ agar plate and the plate was incubated at 30°C to select for colonies that had acquired the desired mutation in *pnp*. After 4 d the first colonies were observed. One was selected and transferred to another M9(glu)^sup^ plate as described for growth experiments. From a single colony obtained on the second M9(glu)^sup^ plate, an LB culture without antibiotics was inoculated and grown overnight at 37°C. A cryo stock was prepared on the next day and the resulting strain was named Sel_eng_pnp-mut_. The presence of the desired *pnp*:G573S variant and by that the success of the genomic edit were confirmed by colony PCR and Sanger sequencing of the respective locus as described in the previous paragraph. Furthermore, WGS of Sel_eng_pnp-mut_ confirmed the introduction of the *pnp* mutation and no additional genomic mutation in comparison to the reference strain Sel_aux_ was observed. Subsequently, all plasmids harbored by Sel_eng_pnp-mut_ were cured as described above, and the obtained plasmid-free strain was termed Sel_aux_pnp-mut_.

#### Strain Sel^0^_hit_*

Genomically integrated antibiotic resistance cassettes were removed from the strain Sel^0^_hit_ by Flp-catalyzed excision using the same protocol as described earlier for Sel_aux_. The success of the removal of the antibiotic cassettes from the genome was verified by colony PCR and Sanger sequencing of the respective loci as described previously. WGS was performed with the resulting strain Sel^0^_hit_*. The removal of the antibiotic cassettes was confirmed and no additional genomic mutations in comparison to the progenitor strain Sel^0^_hit_ were observed.

### Reverse transcription-quantitative PCR

Sel_aux_* (wildtype *pnp* gene) and Sel^0^_hit_* (*pnp*:G573S) were transformed with the plasmid pMiniHisT_TAC__pur. Individual transformants were used to inoculate 2 ml of LB precultures and grown overnight. The next day, a 5 ml LB culture was inoculated from each preculture (1:100 dilution) and grown until an OD_600_ of 1 was reached. Of this, 0.8 ml were used for RNA extraction using the PureLink RNA Mini Kit (Thermo Fisher Scientific) according to the manufacturer’s protocol, with on-column DNA digestion using the PureLink DNase Set (Thermo Fisher Scientific) as per the manufacturer’s instructions. The resulting RNA preparations (stored at −80°C) served as input in the next step.

Reverse transcription-quantitative PCRs (RT-qPCRs) were performed using the Luna Universal One-Step RT-qPCR kit (New England BioLabs) according to the manufacturer’s protocol. The reverse transcription was performed at 60°C to denature tRNAs. RT-qPCRs were conducted with a QuantStudio 3 Real-Time PCR system (Thermo Fisher Scientific). QuantStudio software was used to obtain background corrected fluorescence values. Reactions were performed in MicroAmp optical 96 well reaction plates (Thermo Fisher Scientific) sealed with MicroAmp optical adhesive film (Thermo Fisher Scientific). Concentration of RNA was determined using a Nanodrop 2000 spectrophotometer (Thermo Fisher Scientific). After reverse transcription, two targets, *mini hisT_TAC_* (amplicon length 59 bp) and the housekeeping gene *ihfB* (amplicon length 101 bp) as a reference, were amplified by qPCR to allow for relative quantification of *mini hisT_TAC_* expression levels (Supplementary Table S3) (24,25). Primer pairs and qPCR conditions were validated by qPCR standard curves and melting curves of PCR products. Standard curves for both targets were generated from RT-PCRs performed with one biological sample (in technical triplicates) starting from different amounts of template RNA (1, 3, 10, 30 and 60 ng) by a linear fit generated using OriginPro 2021b (OriginLab Corporation). A fluorescence threshold of 70 000 was used for both targets, which was located in the exponential area of the amplification curves. Samples without any RNA template or with 30 ng of RNA template but without reverse transcription were used as negative controls (both in technical triplicates). The qPCR efficiency = 10^(−1/m)^−1 was calculated from a standard curve with m as the slope of the curve. To measure relative expression levels between two strains, RT-qPCR amplification curves were recorded for each strain in biological triplicates. For each biological sample, *C*_T_ values of the mean amplification curves (mean of technical triplicates) were determined. We calculated Δ*C*_T_ = *C*_T_ (*ihfB*) – *C*_T_ (*mini hisT_TAC_*) for each biological sample and the mean Δ*C*_T_ ± 1 standard deviation (sd) for the two strains. The *p*-value of the difference in expression levels was calculated using an unpaired two-sided *t*-test assuming equal variance (RStudio 4.2.1). The ΔΔ*C*_T_ value was defined as ΔΔ*C*_T_ = Δ*C*_T_(Sel^0^_hit_* harboring plasmid pMiniHisT_TAC__pur) - Δ*C*_T_(Sel_aux_* harboring plasmid pMiniHisT_TAC__pur). The SD of ΔΔ*C*_T_ was determined via propagation of uncertainty. The fold change of expression levels was calculated as 2^−ΔΔCT^.

### Production and purification of HisS

Production of the protein HisS was performed similarly to a previously described method (26). Briefly, BL21(DE3) was transformed with the pHisS_tst_pur plasmid and 1 % (w/v) glucose was added to the LB plate used to select for transformed cells. A 4 ml TB medium preculture containing 1 % (v/v) glucose was inoculated from a single clone, grown overnight, and used to inoculate 1 l TB medium, which was subsequently grown until an OD_600_ of 1. Then, IPTG was added to 1 mM to induce protein production, and the culture was further incubated for 5 h under the same conditions. Cells were harvested by centrifugation (5000 *g*, 30 min, 4°C), the supernatant was discarded, and the pellet was frozen in liquid nitrogen and stored at −80°C.

For lysis, the pellet was resuspended in 30 ml buffer 1 (50 mM Tris-HCl, 300 mM NaCl, 1 mM DTT, pH 8.0 adjusted with HCl) and subjected to three cycles of homogenization using an Emulsiflex-C3 (Avestin) at 1000–1500 bar, while cooling the cells between cycles. The obtained lysate was centrifuged (20 000 *g*, 4°C, 30 min) and the supernatant filtered (0.45 μm pore size). HisS was purified by affinity chromatography performed at 4°C, utilizing the C-terminal twin-strep-tag. The clarified lysate was applied to a self-packed and pre-equilibrated (with buffer 1) gravity flow column containing Strep-Tactin Sepharose chromatography resin (IBA Lifesciences) with a column volume (CV) of 5 ml. The column was washed with 6 CVs of buffer 1, followed by an elution with 3 CVs of buffer 1 supplemented with 5 mM desthiobiotin. Elution fractions were pooled and concentrated at 4°C using an Amicon centrifugal concentrator (3 kDa molecular weight cutoff, Merck) according to the manufacturer’s protocol. Subsequently, size exclusion chromatography (SEC) was performed with an Äkta Go protein purification system (Cytiva) at 4°C, utilizing a Hiload 16/60 superdex 200 column (Cytiva) pre-equilibrated with buffer 2 (50 mM HEPES-KOH, 30 mM KCl, 3 mM MgCl_2_, 1 mM DTT, pH 7.5 adjusted with KOH) at a flow rate of 0.5 ml min^−1^. The absorbance at 280 nm (A280) was measured during the run, fractions of interest (SEC peak S2) were collected, frozen in liquid nitrogen and stored at −80°C.

Proteins contained in lysate, in fractions from affinity chromatography and from size-exclusion-chromatography were separated by standard sodium dodecyl sulfate–polyacrylamide gel electrophoresis (SDS-PAGE). In brief, samples were mixed with Lämmli buffer, incubated at 98°C for 5 min and then applied to the SDS-PAGE gel. Precast SurePage Bis-Tris 4–20 % gradient gels (Genscript) were run for 40 min at 180 V in the provided Tris-MOPS-SDS running buffer (Genscript). A PageRuler Plus Prestained Protein Ladder 10–250 kDa (Thermo Fisher Scientific) served as a molecular weight marker. Gels were stained using the QuickBlue Protein Stain (LubioScience GmbH) according to the manufacturer’s protocol.

### tRNA production and purification of specific tRNAs

For tRNA production, Sel_aux_ or Sel^0^_hit_ were transformed with one of the tRNA expression plasmids pHisT_TAC__pur or pMiniHisT_TAC__pur. Precultures of 4 ml LB medium were inoculated from individual colonies obtained from the transformations and grown overnight. The next day, these precultures were used to inoculate 400 ml of LB medium (1:100 dilution). The cultures grew to an OD_600_ of 0.6–0.8. Cells were centrifuged (4000 *g*, 10 min, 4°C) and the supernatant discarded.

Total RNA extraction and purification of specific tRNAs was performed similarly to a previously reported method (27). For total RNA extraction, the pellet was resuspended in 5 ml of buffer 3 (50 mM sodium acetate (NaOAc), 10 mM Mg(OAc)_2_, pH 5.2 adjusted with AcOH) and 5 ml of water-saturated phenol. The sample was incubated at RT at 1000 rpm in a ThermoMixer C (Eppendorf) for 1 h and subsequently centrifuged (4000 *g*, 10 min). The same shaking incubator was used in the following steps for RNA extraction and tRNA purification. The aqueous phase was transferred to a new tube, mixed with 5 ml of chloroform, thoroughly vortexed and then centrifuged (4000 *g*, 5 min). The aqueous phase was transferred into another tube. For an isopropanol precipitation, 0.1 volume of 3M NaOAc pH 5.0 and 0.7 volume isopropanol were added to the sample. The sample was incubated for 30 min at 4°C and then centrifuged (15 000 *g*, 15 min, 4°C). After removal of the supernatant, the pellet was washed with 1 ml ice of cold 70 % isopropanol and centrifuged again (15 000 *g*, 10 min, 4°C). The supernatant was removed, the pellet was air dried at RT for 15 min and stored at −80°C. The pellet was resuspended in 0.8 ml of TRIzol reagent (Thermo Fisher Scientific) and incubated for 5 min at RT. The sample was mixed with 0.16 ml chloroform and vortexed until no phase separation was visible any longer. Then, the sample was incubated for 2 min at RT and centrifuged (12 000 *g*, 15 min, 4°C). For a second isopropanol precipitation, the aqueous phase was transferred into a new tube containing 0.4 ml of isopropanol. The sample was incubated for 10 min at RT and centrifuged (12 000 *g*, 10 min, 4°C). The supernatant was discarded, the pellet was washed with 0.8 ml of ice cold 75 % of ethanol and the sample was centrifuged (7500 *g*, 5 min, 4°C). After removal of the supernatant, the pellet was air dried at RT for 10 min and resuspended in 0.5 ml of buffer 4 (30 mM HEPES-KOH, 1.2 M NaCl, 15 mM EDTA, pH 7.0 adjusted with KOH). The sample was incubated for 10 min at 55°C. The concentration of the extracted total RNA was determined by the absorbance at 260 nm (A260).

For the purification of a specific tRNA from the extracted total RNA, 200 μl of Streptavidin high performance resin (Cytiva) was centrifuged (10 000 *g*, 1 min). The supernatant was discarded, and the beads were resuspended in 1 ml buffer 5 (10 mM HEPES-KOH, 0.4 M NaCl, 5 mM EDTA, pH 7.0 adjusted with KOH). The washing step was repeated four more times. Afterwards, the beads were centrifuged again, and the supernatant discarded. The beads were resuspended in 1 ml of a 3′ biotinylated probe probeMPE2 (3 μM) (Supplementary Table S3) in buffer 5. The beads were incubated with the probe for 1 h at 1400 rpm. Then, the beads were washed three times as described before with 1 ml of buffer 5. The beads were centrifuged again, and the supernatant discarded. The beads were resuspended in 0.5 ml of buffer 4 and 200 μl of the extracted total RNA were added. The sample was incubated for 30 min at 68°C at 1400 rpm. The sample was cooled down on ice for 1 min and washed three times as described previously with 0.5 ml of buffer 6 (15 mM HEPES-KOH, 0.6 M NaCl, 7.5 mM EDTA, pH 7.0 adjusted with KOH) and two times with 0.5 ml of buffer 7 (0.5 mM HEPES-KOH, 20 mM NaCl, 0.25 mM EDTA, pH 7.0 adjusted with KOH). For elution, the beads were centrifuged and resuspended in 500 μl of buffer 7 for the purification of HisT_TAC_ and in 100 μl of buffer 7 for the purification of mini HisT. After an incubation for 10 min at 80°C and 1400 rpm, the beads were centrifuged, and the supernatant was collected to obtain the first elution fraction. For the purification of HisT_TAC_ this procedure was repeated with 200 μl of buffer 7 and for mini HisT with 100 μl until no RNA was detected in the elution fractions, as determined by A260 measurements.

The RNA in the obtained sample from the purification process was separated by denaturing PAGE performed with Novex 10 % TBE 7 M urea gels (Thermo Fisher Scientific) as per the manufacturer’s protocol (80 min at 180 V). To prepare the samples for the denaturing PAGE, a sample aliquot of 10 μl was mixed with 10 μl of Novex TBE urea sample buffer (Thermo Fisher Scientific). A low range ssRNA ladder (New England Biolabs) was used as a size marker, prepared according to the manufacturer’s protocol. The gels were stained for 25 min with 50 ml of SYBR gold solution (Thermo Fisher Scientific) according to the manufacturer’s guidelines.

### Aminoacylation assay

Purified tRNAs were first subjected to a deacylation step prior to their use in the assay, ensuring they were not charged with an amino acid. To achieve this, buffer 8 (15 mM Tris-HCl, pH 9.0 adjusted with HCl) from a 100× stock was diluted to 1× in pooled tRNA elution fractions (obtained from bead elution steps). The tRNAs were incubated for 45 min at 37°C in the alkaline buffer to achieve deacylation. Afterward, an ethanol precipitation was performed by adding 0.1 volume of 3 M sodium acetate (NaAc), 2.5 volumes of ethanol and 0.08 mg ml^−1^ UltraPure Glycogen (Thermo Fisher Scientific) to the sample. The sample was incubated for 1 h at −20°C and then centrifuged (12 000 *g*, 15 min, 4°C). The supernatant was discarded, the pellet washed with 500 μl of 75 % ethanol and centrifuged (12 000 *g*, 15 min, 4°C). The supernatant was discarded, the pellet was air dried at RT for 10 min, and then resuspended in 50 μl of water. The deacylated tRNA was stored at −80°C.

The assay to detect aminoacylation of a tRNA was performed similarly to a previously reported method (28). An aliquot of 1 μl of buffer 9 (10 mM Tris-HCl, 10 mM MgCl_2_, pH 7.5 adjusted with HCl) was added to 9 μl of the tRNA sample stemming from the ethanol precipitation. The sample was incubated for 3 min at 80°C, cooled to RT and then used for aminoacylation. The 15 μl aminoacylation reaction, conducted in buffer 10 (20 mM Tris-HCl, 20 mM KCl, 10 mM MgCl_2_, pH 7.5 adjusted with HCl), contained 1 μM tRNA, 10 mM DTT, 0.35 mM histidine, 0.1 mg ml^−1^ BSA, 20 nM purified HisS (sample from SEC peak S2 used) and 6 mM ATP. Aminoacylation was performed at 37°C for 1 h and stopped with addition of 22.5 μl quenching solution (0.25 M NaAc pH 5.0, 0.44 mg ml^−1^ glycogen, 10 mM EDTA). For ethanol precipitation, an aliquot of 87.5 μl of ethanol was added and the sample was centrifuged (15 000 *g*, 15 min, 4°C). The supernatant was discarded and the pellet was washed with 500 μl 70% of ethanol containing 0.1 M NaAc pH 5.0 to maintain an acidic pH to prevent ester hydrolysis. The sample was centrifuged again (15 000 *g*, 15 min, 4°C), the supernatant was discarded and the pellet air dried at RT until no liquid remained in the sample. The pellet was resuspended in 15 μl of buffer 11 (60 mM HEPES-KOH, pH 8.0). For the biotinylation of amino acid tRNA conjugates, 60 μl of a freshly prepared 15 mM solution of Sulfo-NHS-Biotin (Thermo Fisher Scientific) in buffer 11 were added and the sample was incubated for 1 h at 4°C. Afterward, 7.5 μl of 3 M NaAc pH 5.0, 5.4 μl of 5 mg ml^−1^ glycogen and 210 μl of ethanol were added for an ethanol precipitation. The remainder of the ethanol precipitation was performed as described above. The final pellet was resuspended in 7.3 μl of buffer 12 (10 mM HEPES-KOH pH 7.0). For streptavidin conjugation, 2.7 μl of 50 μM streptavidin in PBS was added to the sample (for 9-fold molar excess of streptavidin in relation to the tRNA). The sample was incubated for 20 min at RT. Aminoacylation was detected by a gel shift on a denaturing PAGE, performed as described for the purification of specific tRNAs.

### *In vitro* protein synthesis

*I* *n vitro* protein synthesis was performed with the PUREexpress Δ(aa, tRNA) kit (New England Biolabs). For control PURE reactions to which neither mini HisT nor HisS were added, the amino acid mixture of the kit was replaced with a self-made amino acid mix containing all 20 amino acids (300 μM each) in buffer 12. Otherwise, the *in vitro* protein synthesis for these samples was performed according to the manufacturer’s protocol (250 ng of template plasmid pDHFRvar, 25 μl reaction, incubation for 2 h at 37°C) with the recommended addition of 25 U of RNase Inhibitor Murine (New England Biolabs).

Mini HisT was pre-aminoacylated before using it for *in vitro* protein synthesis as described above. After the ethanol precipitation before biotinylation, the obtained pellet was resuspended in 5 μl of buffer 11 and sample was used for *in vitro* protein synthesis. For PURE reactions in which HisS and mini HisT or only HisS were added, an amino acid mix without valine containing only 19 amino acids (300 μM each) in buffer 12 was used instead of the amino acid mixture of the kit. By omitting valine from the mix, we aimed to lower the valine content in the PURE reaction to avoid that the *E. coli* valine tRNA outcompetes mini HisT in decoding the suppression target codon. To the PURE protein synthesis mix, 20 nM purified HisS (sample from SEC peak S2 used) and 100 nM aminoacylated mini HisT were added. Otherwise, the *in vitro* protein synthesis for these samples was performed as described for control samples above. All PURE reactions were performed at 37°C, the standard temperature of the kit. The effect of a lower temperature was not tested here.

Proteins in the PURE reaction were separated by SDS-PAGE as described above for the purification of HisS. A Precision Plus Protein Dual Xtra Standard (BioRad) served as a molecular weight marker.

### Peptide mass fingerprinting

Sample digestion, liquid chromatography-mass spectrometry/mass spectrometry (LC-MS/MS) and the peptide identification were performed by the Functional Genomics Center Zurich as described below. Gel bands of DHFR variants were cut into small pieces, washed twice in 100 μl wash solution (100 mM NH_4_HCO_3_, 50 % (v/v) acetonitrile) and once in acetonitrile. The supernatant was discarded after each wash step. The proteins were digested using 200 ng Endoproteinase Asp-N Sequencing Grade (Roche) in 30 μl of digestion buffer (10 mM Tris, 2 mM CaCl_2_, pH 8.2). The digestion was carried out overnight at 37°C. The supernatants were collected, and the peptides were extracted from the remaining gel pieces by resuspending them in 150 μl of 50% acetonitrile with 0.1% trifluoroacetic acid for 15 min in an ultrasonic bath. The supernatants were combined, and the samples were dried to completeness in air and resolubilized in 20 μl of MS sample buffer (3% acetonitrile, 0.1% formic acid).

MS analysis was performed on an Orbitrap Exploris 480 mass spectrometer (Thermo Fisher Scientific) equipped with a Nanospray Flex Ion Source (Thermo Fisher Scientific) and coupled to an M-Class UPLC (Waters). Solvent composition at the two channels was 0.1 % formic acid for channel A and 99.9 % acetonitrile with 0.1 % formic acid, for channel B. Column temperature was 50°C. For each sample, 3 μl of peptide solution was loaded on a commercial nanoEase MZ Symmetry C18 Trap Column (100 Å, 5 μm, 180 μm × 20 mm, Waters) followed by a nanoEase MZ C18 HSS T3 Column (100 Å, 1.8 μm, 75 μm × 250 mm, Waters). The peptides were eluted at a flow rate of 300 nl min^−1^. After a 3 min initial hold at 5% B, a gradient from 5 to 22% B in 40 min and 22 to 32% B in an additional 5 min was applied. The column was cleaned after the run by increasing to 95% B and holding 95% B for 10 min prior to re-establishing loading condition at 5% B for another 10 min.

The mass spectrometer was operated in data-dependent mode (DDA) combined with an inclusion list for the peptide precursors from the protein of interest. The spray voltage was set to 2.3 kV, funnel RF level at 40 %, heated capillary temperature at 275°C, and Advanced Peak Determination (APD) on. Full-scan MS spectra (350−1200 *m/z*) were acquired at a resolution of 120 000 at 200 *m/z* after accumulation to a target value of 3 000 000 or for a maximum injection time of 45 ms. Precursors with an intensity above 5000 were selected for MS/MS. Ions were isolated using a quadrupole mass filter, with a 1.2 *m/z* isolation window and fragmented by higher-energy collisional dissociation (HCD) using a normalized collision energy of 30%. HCD spectra were acquired at a resolution of 60 000 and maximum injection time was set to 200 ms. The automatic gain control (AGC) was set to 100 000 ions. Charge state screening was enabled such that single, unassigned and charge states higher than six were rejected. Precursor masses previously selected for MS/MS measurement were excluded from further selection for 20 s, and the exclusion window was set at 10 ppm. The samples were acquired using internal lock mass calibration on *m/z* 371.1012 and 445.1200. The mass spectrometry proteomics data were handled using the local laboratory information management system (LIMS) (29). For peptide identification, the acquired raw MS data were identified using the PEAKS search engine (Bioinformatics Solutions inc., version 10.5). Spectra were searched against the Uniprot *E. coli* proteome database (taxonomy 83 333, version from 12 July 2021) and sequences of the DHFR variants. Methionine oxidation was set as variable modification, and enzyme semi-specificity was set to AspN, allowing a maximum of two missed cleavages. A fragment ion mass tolerance of 0.02 Da and a parent ion tolerance of 10 ppm were set. Scaffold (Proteome Software Inc., version 5.10) was used to validate MS/MS based peptide and protein identifications. Peptide identifications were accepted if they achieved a false discovery rate (FDR) of <0.1 % by the Scaffold Local FDR algorithm.

### RNA structure modeling

De novo models of mini HisT were calculated via FARFAR2, a Rosetta-based modeling tool for the prediction of the lowest energy 3D structure for a provided RNA sequence (30,31). The nucleotide sequence of mini HisT and the hydrogen bonds in the remaining stems were used as inputs for the prediction on the web-server (https://rosie.rosettacommons.org/farfar2). A total of 2000 models were obtained and the best scoring one according to the internal FARFAR2 scoring criterion was selected for representation.

## Results

### Identification of a suitable suppression target

We began crafting a vitamin-based genetic suppression system that couples the activity of a suppressor tRNA to the growth of an *E. coli* strain by selecting an essential target gene for suppression. The gene encoding the enzyme that catalyzes the last step in the thiamine phosphate biosynthesis in *E. coli* was deemed a suitable target. The enzyme condenses the pyrimidine and the thiazole moieties of thiamine phosphate (32). No other biosynthesis pathway containing molecules similar in chemical structure to thiamine is known in *E. coli*, which should minimize the likelihood that an alternative biosynthesis pathway could emerge to produce thiamine in the absence of the enzyme. The native *E. coli* enzyme, ThiE, does not contain a suitable target residue, whose codon might function as the suppression target site. As such, we looked for alternative enzymes in the thiamine biosynthesis pathways of other organisms. The ThiN domain of the two-domain protein ThiDN from *Thermotoga maritima* MSD8 catalyzes the same reaction as ThiE and the two proteins are nonhomologous. The expression of *thiDN* successfully complemented the deletion of *thiE* in *E. coli* (33). Importantly, a histidine residue in ThiN from *Pyrococcus furiosus* was reported to be essential for enzyme function and suggested to be involved in catalysis (19). Taken together, we reasoned that a mutated *T. maritima thiN*, altered in the codon for the respective H95, could serve as a suitable suppression target in an *E. coli thiE* deletion strain. However, overexpression of *yjbQ* compensated for the absence of *thiE* (34). Hence, we constructed a strain lacking *yjbQ* and *thiE* (Sel_aux_) to set up the suppression system.

Next, we assessed the effect of all 20 amino acid residues at position 95 in ThiN in the thiamine auxotrophic strain Sel_aux_ on selective M9 minimal medium plates supplemented with glucose as carbon source (M9(glu)). As expected, the expression of wildtype (wt) *thiN* conferred growth (Supplementary Figure S4). In contrast, no growth was observed with any other of the 19 ThiN_H95X variants, demonstrating that H95 is indeed essential (Supplementary Table S4). The codon for H95 in ThiN was thus deemed suitable as a suppression target site.

To choose the most suitable codon to replace the H95 codon, we considered the chance for reversion to a histidine codon, which would render the genetic system obsolete. We considered all 18 possible codons that differ in all three bases from any histidine codon, constructed the corresponding *thiN* gene variants and individually expressed them in Sel_aux_. The resulting 18 strains were tested in an adaptive evolution experiment by incubating them on selective M9(glu) plates for 14 d and in this way selecting for growing strains (Supplementary Table S5). Individual colonies indicating escape events emerged for all four *thiN* variants with serine and arginine codons at the target site. Of the 14 remaining *thiN* variants, which did not yield colonies in the experiment, some can in principle be reverted more easily to a serine or arginine codon than others. We concluded that the ideal target codons for suppression are the GTG and GTA valine codons: they differ in all three bases from any histidine codon, in two bases from any arginine and serine codon, and they did not yield escapees in the adaptive evolution experiment. Consequently, a specific *thiN* variant (termed thiN:H95V), in which the histidine codon encoding amino acid 95 was changed to a GTA valine codon, was selected as our final suppression target gene for the system.

### Crafting and testing of the complete suppression system

**Figure 1.**
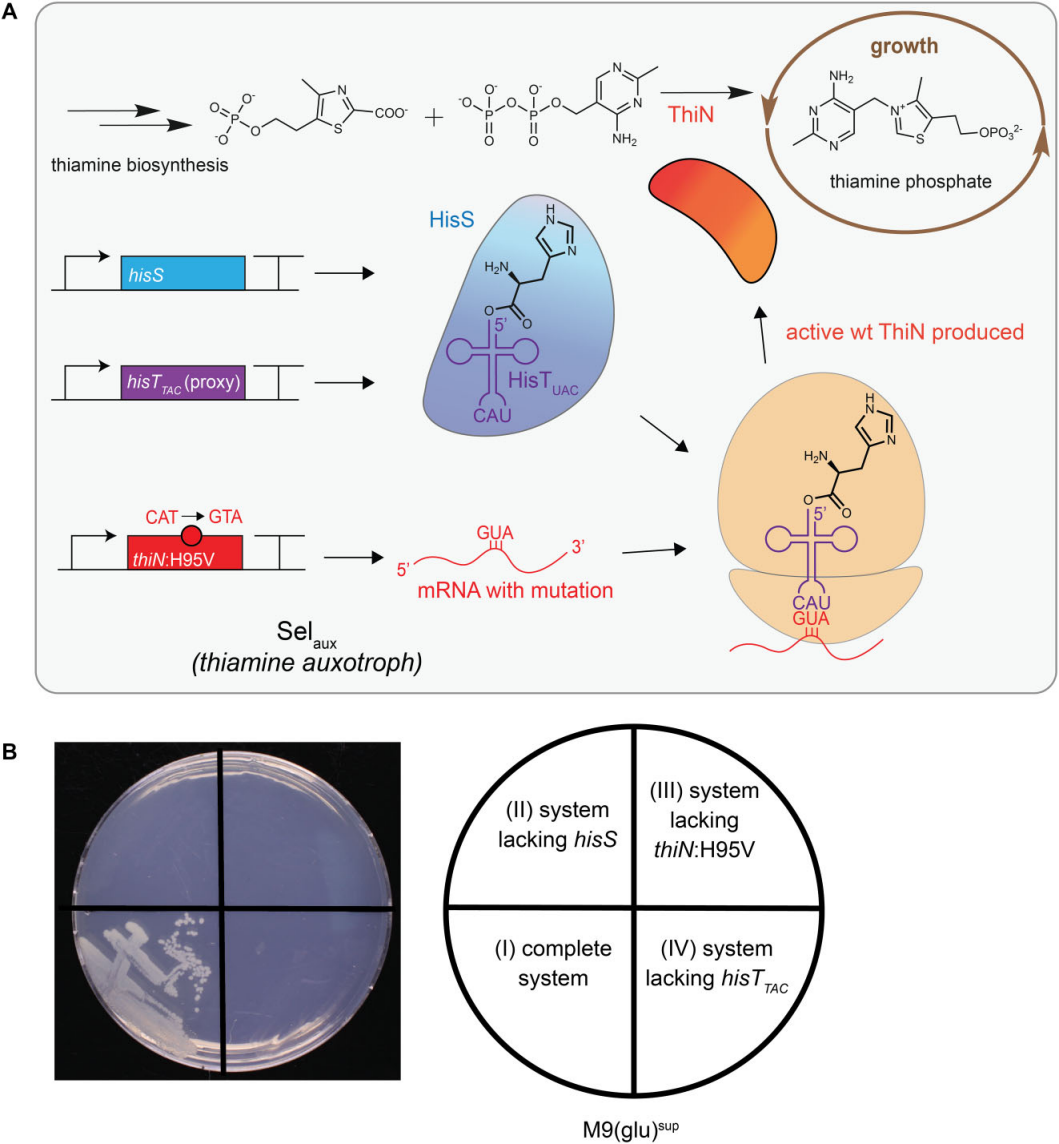
ThiN-based suppression system. (**A**) Schematic representation of the suppression system. The growth of the host strain Sel_aux_ is coupled to the activity of the otRNA HisT_UAC_. The thiamine auxotrophy of strain Sel_aux_ can be complemented by ThiN. A *thiN* variant in which the target codon of the essential amino acid residue H95 is mutated to a GTA valine codon is expressed. The ThiN H95V variant, produced according to the classical genetic code, is inactive. HisT_UAC_ is charged with histidine by HisS and can suppress the mutated codon. This leads to the production of active ThiN (containing H95) from the *thiN*:H95V gene, cures the auxotrophy and makes HisT_UAC_ essential to the strain. For adaptive evolution experiments, HisT_UAC_ can be substituted by non-canonical tRNA folds that are not active in translation initially. For the strain to grow, it must evolve to accept the non-canonical fold in translation. (**B**) The complete suppression system is required to confer thiamine prototrophy to Sel_aux_. The thiamine auxotrophic strain Sel_aux_ was transformed with plasmids encoding the complete or incomplete (lacking one component) versions of the suppression system. The strains harbored the following plasmids: (I) complete system: pHisS_HisT_TAC_ and pThiN(H95V_GTA_); (II) pHisT_TAC_ and pThiN(H95V_GTA_); (III) pHisS_HisT_TAC_ and pSEVA191(empty backbone); (IV) pHisS and pThiN(H95V_GTA_). Strains were incubated on a M9(glu)^sup^ agar plate for 3 d. This experiment was repeated three independent times obtaining the same result each time.

Next, we turned to the selection of a suitable orthogonal histidine tRNA and the construction of a suppressor variant that reads out the GUA valine codon as histidine. In the sole *E. coli* histidine tRNA, the base pairing of G-1 and C73 is a major recognition element for the respective ARS (35). The histidine tRNA/ARS pairs of certain alphaproteobacteria were considered to be orthogonal in *E. coli* (26). These tRNAs lack the G-1 and the described interaction, relying on other recognition elements for their cognate ARS. This includes the tRNA HisT from the alphaproteobacterium *Paracoccus denitrificans*. We selected HisT and the cognate synthetase HisS as a novel orthogonal pair for our suppression system and in the remaining article, *hisS*/*hisT* and HisS/HisT refer to the genes and their expression products from *Paracoccus denitrificans* PD1222. The anticodon of HisT was adapted to recode the chosen GUA valine codon in *thiN*:H95V mRNA as histidine and we integrated the resulting missense suppressor gene *hisT_TAC_* and *hisS* into the system (Figure 1A). In summary, HisT_UAC_, after being charged by HisS, is utilized to read out the GUA codon in the *thiN*:H95V mRNA and this should result in a sufficient number of instances in the incorporation of histidine at position 95 in ThiN curing the thiamine auxotrophy of Sel_aux_.

In order to further increase the sensitivity of the suppression system, we developed a new selective medium termed M9(glu)^sup^. It contains metabolites for whose production otherwise thiamine-containing enzymes would be required and thus lowering the thiamine requirement for growth of Sel_aux_ (Supplementary Text S1, Supplementary Figures S5-S7 and Supplementary Table S6). The addition of ∼1000 thiamine molecules per cell was sufficient to achieve full non-thiamine limited growth in the optimized selective medium (Supplementary Figure S8). M9(glu)^sup^ was adopted as the selective medium for all subsequent experiments.

Sel_aux_ cells containing the complete plasmid-based suppression system grew on M9(glu)^sup^ agar plates (Figure 1B). Conversely, when Sel_aux_ cells harboring incomplete versions of the system, lacking either *hisS* or *thiN*:H95V or *hisT_TAC_*_,_ were plated on M9(glu)^sup^ agar, no colonies were observed. This growth pattern could also be observed in liquid M9(glu)^sup^ medium (Supplementary Figure S9). We also observed that the strain harboring the complete suppression system grew in M9(glu)^sup^ medium (i.e. under thiamine-less selective conditions) as fast as under nonselective conditions (supplementation of thiamine in excess). This indicated that the thiamine production in the cell due to the system was not growth limiting in the optimized medium. Under nonselective conditions, Sel_aux_ containing the complete suppression system grew as fast as Sel_aux_ harboring an incomplete system lacking the suppressor tRNA. Thus, no toxicity from histidine incorporation at valine positions across the proteome by the suppression system was detected. To summarize, our results demonstrate that all parts that we consider components of the system (*hisS*, *thiN*:H95V and *hisT_TAC_*) are essential under selective conditions as we planned when designing the system. Additionally, the absence of growth for a system lacking *hisS* verified that HisT_TAC_ is not charged by the endogenous *E. coli* histidine ARS to a degree that would result in thiamine prototrophy confirming it to be sufficiently orthogonal in *E. coli* to utilize it for the following evolution experiments.

Finally, we assessed whether a strain containing an incomplete suppression system could escape the selection. To this end, we performed adaptive evolution experiments with Sel_aux_ harboring systems lacking either *hisT_TAC_* or *hisS* to detect potential escapees. The strains were incubated in liquid medium and on agar plates to test whether an escape event occurred. Specifically, approximately 5 × 10^11^ cells were incubated in M9(glu)^sup^ medium for 30 d and approximately 1.5 × 10^9^ cells on M9(glu)^sup^ agar plates for 26 d (Supplementary Table S7). No escape was observed resulting in an escape frequency (for the given time periods) of <2 × 10^−12^ and <7 × 10^−10^, respectively. We conclude that the dependence on the suppression system in the Sel_aux_ host strain under selective conditions appears to be absolute. Therefore, we considered the ThiN-based suppression system suitable for long-term adaptive evolution experiments to select for strains utilizing non-canonical adaptors of the genetic code.

### Evolution of *E. coli* to utilize non-canonical tRNA folds in translation

**Figure 2.**
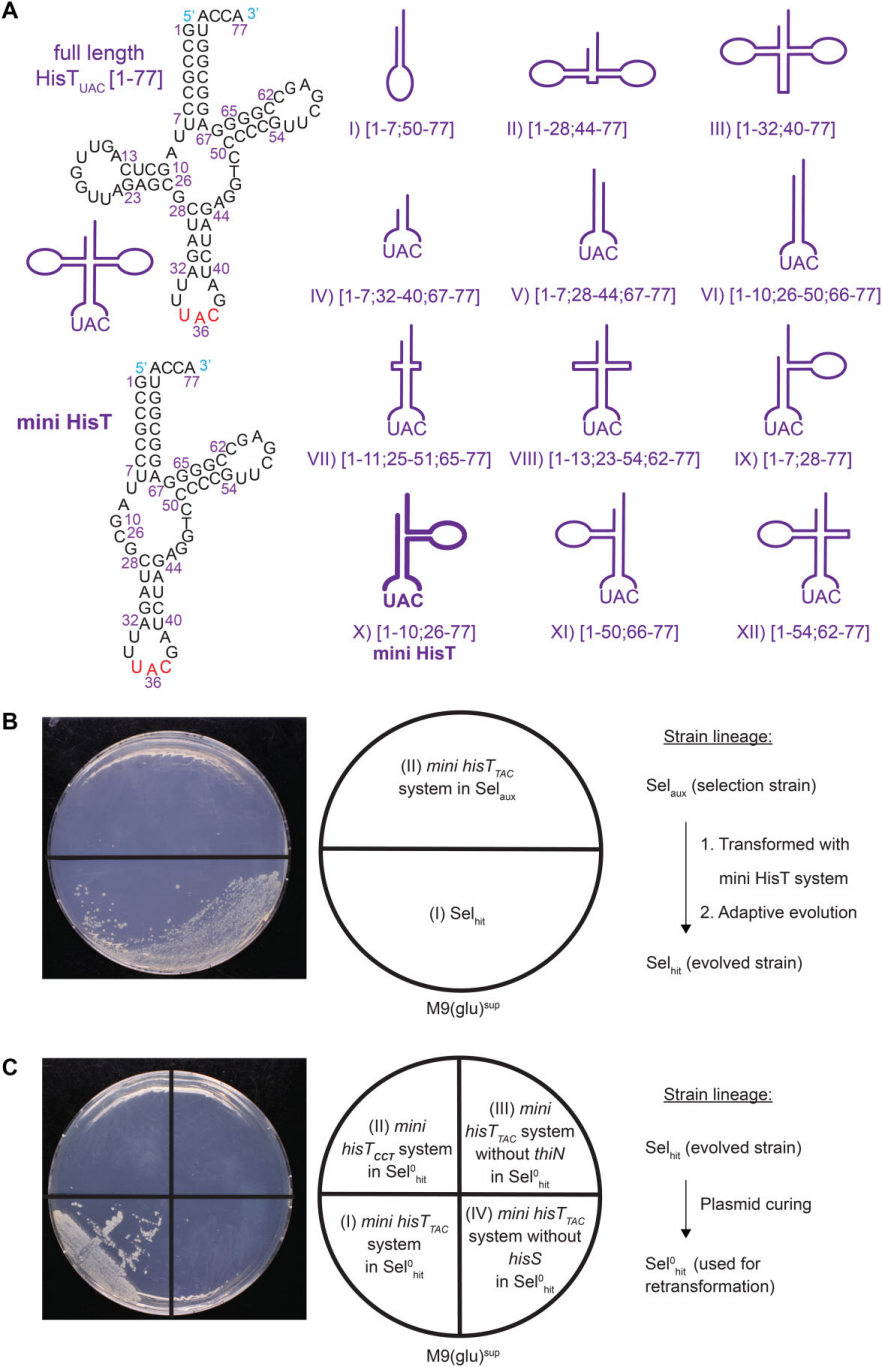
Non-canonical tRNA folds and utilization of mini HisT in translation of living *E. coli*. (**A**) HisT and HisT-derived non-canonical tRNA designs. At the top of the left side, full-length HisT_UAC_ with base numbering (anticodon in red) and its schematic depiction are shown. On the right side, the tested non-canonical tRNA designs I-XII are displayed schematically, with the maintained nucleotides in brackets (for full sequences of all designs see Supplementary Figure S14). These were employed in a long-term adaptive evolution experiment attempting to evolve the respective strains to utilize the novel tRNA designs in translation. Henceforth, design X was named mini HisT. At the bottom of the left side, the sequence of mini HisT is shown with base numbering according to the full-length HisT_UAC_ sequence. (**B**) Growth of the evolved and the progenitor strain under selective conditions. A M9(glu)^sup^ agar plate was incubated for 5 d with the following strains: (I) Sel_hit_ isolated from adaptive evolution experiment in which a suppression system containing *mini hisT_TAC_* was used; (II) Sel_aux_ transformed with pHisS_MiniHisT_TAC_ and pThiN(H95V_GTA_) representing the starting point of the evolution experiment that yielded strain in (I). This experiment was repeated three independent times obtaining the same result each time. The lineage of the strains used here is depicted on the right. (**C**) Growth of the evolved strain under selective conditions depends on all components of the suppression system. Sel^0^_hit_ (Sel_hit_ cured of all plasmids) was transformed with the complete or defective *mini hisT_TAC_* containing suppression systems. The strains carried the following plasmids: (I) pHisS_MiniHisT_TAC_ and pThiN(H95V_GTA_) (complete system); (II) pHisS_MiniHisT**_CCT_** and pThiN(H95V_GTA_) (anticodon in *mini hisT* changed); (III) pHisS_ MiniHisT_TAC_ and pSEVA191 (empty backbone without *thiN*:H95V); (IV) pMiniHisT_TAC_ and pThiN(H95V_GTA_) (no *hisS*). Strains were incubated on a M9(glu)^sup^ agar plate for 5 d. This experiment was repeated three independent times obtaining the same result each time. The lineage of the strain used here is depicted on the right.

To the best of our knowledge, no tRNA which lacks a loop or an entire stem-loop and, by that, adopts a fold divergent from the canonical cloverleaf secondary structure and the L-shaped tertiary structure is known to be used in bacterial translation so far. Lacking starting points in nature, we constructed such folds by a naive and drastic design approach starting from the native secondary structure of HisT_UAC_. Loops or entire stem-loops (arms) were removed from the cloverleaf structure of the suppressor tRNA HisT_UAC_ (Figure 2A). The resulting non-canonical tRNA designs missing one or multiple regions of a canonical tRNA were used in an attempt to substitute the suppressor function of HisT_UAC_ in the designed selection system. As expected, Sel_aux_ containing the resulting systems showed no direct growth on M9(glu)^sup^ agar plates for any of the designs (Supplementary Figure S10). Subsequently, adaptive evolution experiments were conducted using the same strains. For this, cells were long-term incubated on M9(glu)^sup^ agar plates to select for growing strains (Supplementary Figure S11 for workflow).

After incubating the strains for 10 d on selective agar plates, growth was observed for one of the designs (Supplementary Table S8). No growth was ever observed for any other design by the end of the experiment (21 d). The strain obtained from the selection was named Sel_hit_ and originated from Sel_aux_ containing a suppression system with the tRNA design X that lacked the entire D-arm (bases 11–25 removed). Sel_hit_ was a thiamine prototroph and grew reproducibly on selective M9(glu)^sup^ medium in contrast to its unevolved progenitor strain (Figure 2B). The D-arm-less tRNA was named mini HisT and the respective gene *mini hisT_TAC_*. Mini HisT comprised 62 nucleotides, whereas the full-length HisT contains 77. Due to the absence of the D-arm, mini HisT cannot adopt the classical cloverleaf secondary fold. Furthermore, it is expected to adopt a non-canonical tertiary fold, since the canonical L-shape results from interactions between the T- and the D-arm missing in mini HisT.

Next, we purified the plasmids bearing the suppression system genes from Sel_hit_. No mutation was observed in the entire plasmids in Sanger sequencing results. We reasoned that Sel_hit_ must have acquired genomic mutation(s) during the adaptation. To confirm this, we cured Sel_hit_ from any plasmid, which resulted in strain Sel^0^_hit_. Then, Sel^0^_hit_ was retransformed with the original plasmid stocks harboring the *mini hisT_TAC_* containing suppression system. This resulted in direct growth on M9(glu)^sup^ agar plates, indicating that Sel_hit_ had indeed acquired one or several genomic adaptations that enabled the utilization of a D-arm-lacking tRNA in translation (Figure 2C). In contrast, Sel^0^_hit_ retransformed with a combination of selection system plasmids that contained defective suppression systems did not show growth. This verified that all components of the *mini hisT_TAC_* containing system are still required to confer thiamine prototrophy in Sel^0^_hit_. The same results were obtained in liquid medium (Supplementary Figure S12). Strikingly, growth under thiamine-less selective conditions (μ_max_ = 0.11 h^−1^) was slower than under nonselective conditions (μ_max_ = 0.44 h^−1^) for the evolved strain Sel^0^_hit_ retransformed with the functional *mini hisT_TAC_* containing suppression system (Supplementary Figure S13). This discrepancy suggests that the growth of the strain was limited by thiamine production, and that suppression allows for the production of sufficient thiamine to support growth, but not for unlimited growth with respect to thiamine. Note that all presented growth experiments were performed at 30°C. Interestingly, Sel^0^_hit_ retransformed with the complete *mini hisT_TAC_* containing suppression system did not show any growth at 37°C in M9(glu)^sup^ medium (data not shown).

### Identification of the adaptation required to utilize mini HisT *in vivo*

The unevolved Sel_aux_ and the evolved Sel_hit_ were subjected to WGS and two mutations were identified in the genome of Sel_hit_ compared to the progenitor strain. A G1717A nucleotide exchange was found in the *pnp* gene that results in a G573S amino acid exchange in polynucleotide phosphorylase (PNPase). The mutated gene was termed *pnp*:G573S. PNPase is an RNA-specific exonuclease and part of the degradosome, a protein complex responsible for RNA degradation (36). PNPase is involved in tRNA maturation and quality control and is responsible for the degradation of unstable tRNA molecules (37). A second mutation was a T48G nucleotide exchange in *aspV*, which encodes an aspartate tRNA, with the mutation situated in the variable loop.

### Role of *pnp*:G573S

**Figure 3.**
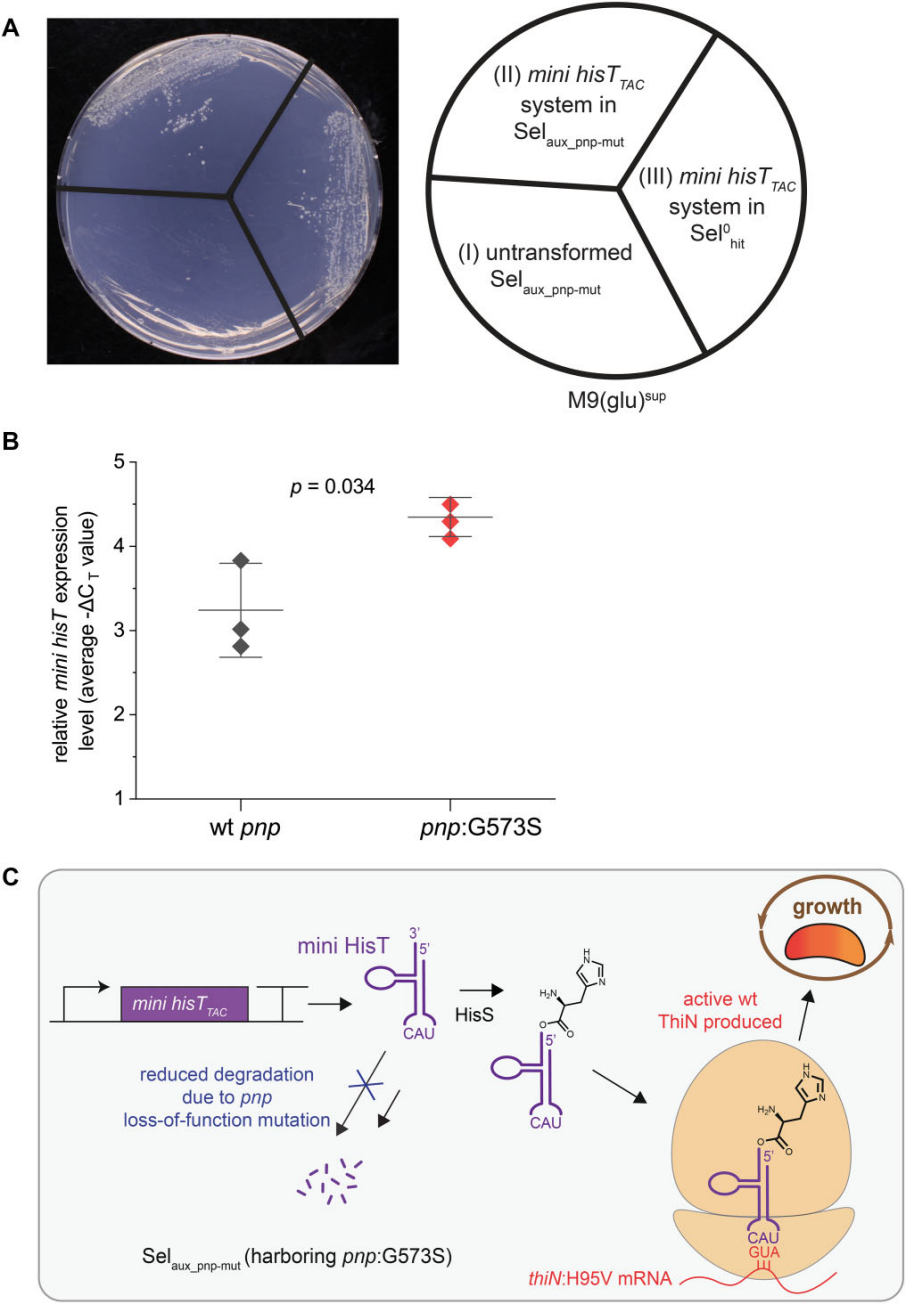
Effect of *pnp*:G573S variant on the utilization of mini HisT. (**A**) *mini hisT_TAC_* confers growth after incorporation of *pnp*:G573S. An M9(glu)^sup^ agar plate without antibiotics was incubated for 6 d with the following strains: (I) Untransformed strain Sel_aux_pnp-mut_ (containing *pnp*:G573S introduced in a targeted manner); (II) Sel_aux_pnp-mut_ transformed with the complete *mini hisT_TAC_*system: pHisS_MiniHisT_TAC_ and pThiN(H95V_GTA_); (III) Sel^0^_hit_ transformed with complete *mini hisT_TAC_*system: pHisS_MiniHisT_TAC_ and pThiN(H95V_GTA_). This experiment was repeated three independent times obtaining the same result each time. (**B**) Relative *mini hisT_TAC_* expression level in strains Sel_aux_* (wt *pnp*) and Sel^0^_hit_* (*pnp*:G573S) measured by qPCR. For both strains, three biological triplicates with the mean ± 1 standard deviation are displayed. The −Δ*C*_T_ value of each biological sample is the average of three technical triplicates. (**C**) Model of mini HisT utilized in translation in Sel_aux_pnp-mut_ (harboring *pnp*:G573S). The G573S amino acid exchange in PNPase reduces the degradation rate of mini HisT by the degradosome, leading to increased intracellular levels. Mini HisT is charged by HisS and accepted as an adaptor of the genetic code in *E. coli*. The suppression of the target codon leads to the production of ThiN containing a histidine as residue 95, which confers thiamine prototrophy.

We hypothesized that *pnp*:G573S caused the acquired thiamine prototrophic phenotype of the evolved strain due to reduced mini HisT degradation. To confirm the hypothesis, we introduced the mutation into Sel_aux_ resulting in strain Sel_aux_pnp-mut_ and verified via WGS that the only mutation in the genome of Sel_aux_pnp-mut_, compared to the progenitor strain Sel_aux,_ was indeed the desired *pnp* mutation resulting in *pnp*:G573S. When Sel_aux_pnp-mut_ was transformed with the *mini hisT_TAC_* system, it showed direct growth (thiamine prototrophy) on selective M9(glu)^sup^ agar plates, in contrast to the untransformed strain (Figure 3A). This was consistent with the thiamine prototrophy observed for the evolved strain Sel^0^_hit_ retransformed with the *mini hisT_TAC_* system. Our results validate the hypothesis that the *pnp* mutation is the only adaptation required for the cell to confer thiamine prototrophy.

To quantify the effect of the mutation, we measured the relative expression levels of *mini hisT_TAC_* (normalized to a reference gene) in strains harboring the wt *pnp* or the *pnp*:G573S gene by RT-qPCR (Supplementary Figures S15-S19). It should be noted that qPCRs were performed with RNA extracted from cells cultivated under nonselective conditions since the unevolved wt *pnp* strain equipped with the *mini hisT_TAC_* containing suppression system does not grow in selective medium. Nevertheless, the difference in Δ*C*_T_ values for the two strains was statistically significant (Figure 3B; Supplementary Figures S20 and S21). A ΔΔ*C*_T_ value of −1.11 ± 0.60 was calculated with a 95% CI of (−2.07, −0.14). This translates to a 2.15-fold increased *mini hisT_TAC_* expression level for the strain containing the *pnp*:G573S variant confirming that the *pnp* point mutation increased the available level of mini HisT in the cell.

Our results confirm that mini HisT is indeed degraded by the degradosome likely due to its non-canonical fold. Furthermore, the G573S amino acid exchange in PNPase impairs the ability of the degradosome to degrade mini HisT. Endogenous valine tRNAs compete with mini HisT for the decoding of the target codon. The *pnp* mutation elevates the levels of HisT beyond a critical threshold enabling it to sufficiently outcompete endogenous tRNAs for the decoding of the GUA codon in the *thiN*:H95V mRNA to produce the required amount of active ThiN molecules for growth (Figure 3C). In conclusion, we demonstrate that a solitary *pnp* mutation is sufficient for the use of mini HisT in translation to a degree that conferred thiamine prototrophy to the previously auxotrophic strain. This indicates that mini HisT is accepted *ab initio* for all required steps of translation in *E. coli*. Mini HisT, a D-arm-less tRNA, is aminoacylated by the cognate synthetase HisS from *P. denitrificans* and used by the involved parts of the *E. coli* translation machinery, namely EF-Tu and the ribosome.

### Aminoacylation of mini HisT by HisS *in vitro*

To further validate our *in vivo* findings that mini HisT is accepted by HisS and by the *E. coli* translation machinery, we reconstituted all involved steps *in vitro*. For this, we isolated mini HisT and HisT_UAC_ from *E. coli* cells that contained the same genetic background as used for suppression experiments (Supplementary Figures S22 and S23). This approach was chosen to obtain molecules that resemble the *in vivo* state as close as possible. Furthermore, HisS containing a C-terminal twin-strep-tag was isolated from *E. coli* (Supplementary Figures S24 and S25).

**Figure 4.**
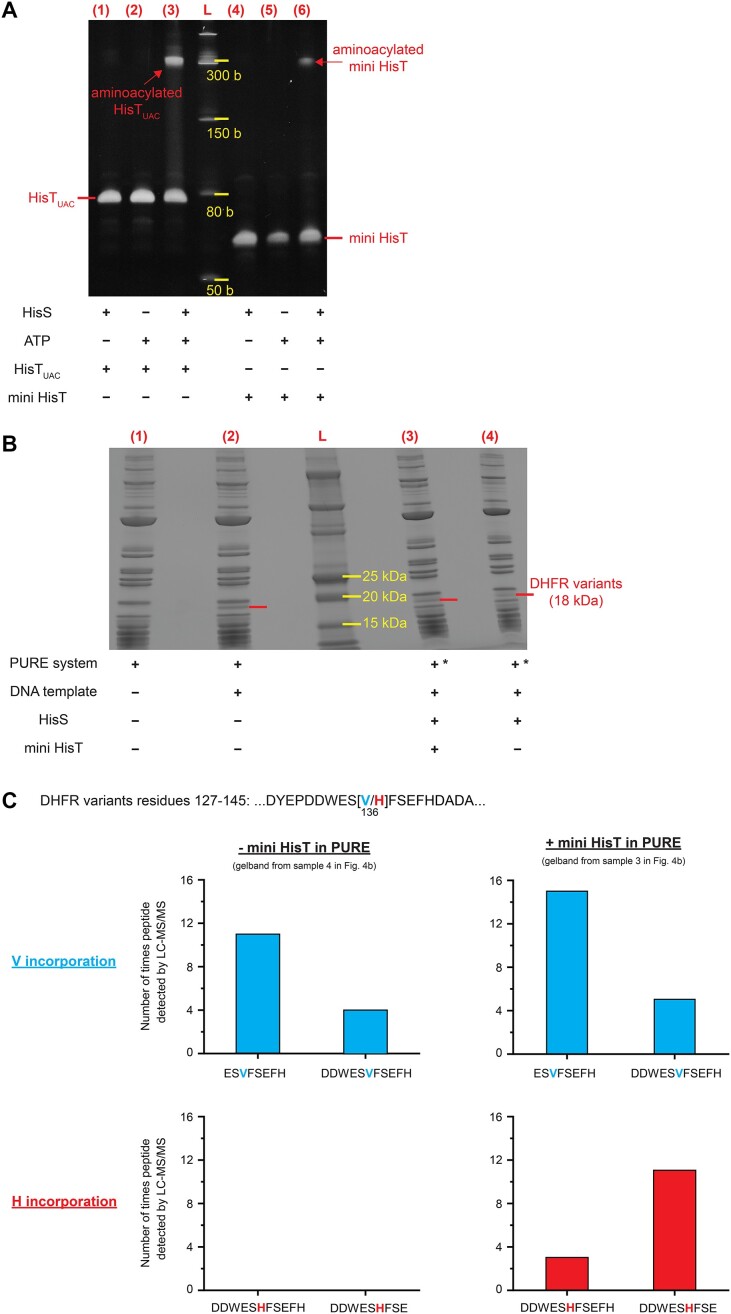
Mini HisT is charged by HisS and accepted by the *E. coli* translation machinery *in vitro*. (**A**) Gel shift assay to confirm aminoacylation by HisS. Purified HisT_UAC_ (lane 3) or mini HisT (lane 6) was incubated with purified HisS, ATP, and histidine. Controls were included in which either HisS (lanes 2 and 5) or ATP (lanes 1 and 4) were omitted. After the aminoacylation reaction, charged tRNA molecules are biotinylated at their α-amino group. Charged and biotinylated tRNAs can be detected upon streptavidin binding as a shifted gel band (red arrow) on the denaturing polyacrylamide gel. (**B**) *In vitro* synthesis of DHFR variants via the PURE system utilizing mini HisT. Expression of a *folA* variant (encoding dihydrofolate reductase, DHFR) with only one valine codon (V136) in the *E. coli* derived PURE cell-free transcription-translation system. SDS-PAGE gel lane 1: standard PURE reaction without DNA template; lane 2: standard PURE reaction for synthesis of DHFR variant; lane 3: PURE reaction for synthesis of DHFR variants in the presence of HisS and pre-histidinylated mini HisT; lane 4: PURE reaction for synthesis of DHFR variants in the presence of HisS. *) For PURE reactions in lanes 3 and 4, a modified amino acid mixture was used to reduce the valine concentration (see ‘Materials and Methods’ section). (**C**) *In vitro* synthesized DHFR variant contains histidine at position 136 due to suppression by mini HisT. DHFR variants were produced in the presence (+ mini HisT) or absence (- mini HisT) of mini HisT in the PURE mix (gel bands from lanes 3 and 4 in Figure 4B). For peptide mass fingerprinting, samples from the proteolytic digest of the DHFR variants were analyzed by LC-MS/MS. Obtained peptide spectra were mapped against the predicted DHFR variants. All identified peptides that contained amino acid residue 136 with their counts are presented in the graphs (top graphs: detected peptides containing valine, bottom graphs: peptides containing histidine). Two independent gel bands corresponding to the size of the DHFR variants were analyzed for each sample, and the aggregated results are presented here (Supplementary Table S9 for individual results and Supplementary Text S2 for discussion of peptide counts and reliability of data).

A radioactivity-free assay was used to confirm that mini HisT is aminoacylated by HisS *in vitro*, while using HisT as positive control (28). In the assay, only aminoacylated tRNAs are biotinylated at the α-amino group of the bound amino acid, which can be visualized by a gel shift upon streptavidin binding. As expected, for HisT_UAC_ a shifted band was only observed if it was incubated with HisS, ATP, and histidine for the aminoacylation reaction (Figure 4A). No shift was observed if either ATP or HisS was omitted in the aminoacylation reaction. The same results were obtained when mini HisT was used as the tRNA substrate. This demonstrates that the D-arm-less mini HisT is indeed aminoacylated by HisS *in vitro*.

### Using mini HisT for *in vitro* translation

To prove that mini HisT is accepted at all steps of translation, we performed *in vitro* protein synthesis assays. The commercially available PURE system is an *in vitro* transcription-translation reaction mix that contains the purified and reconstituted components of the *E. coli* translation apparatus (38). A mutated version of the *E. coli folA* gene was used as a translation reporter. It contains only a single GTA valine codon (coding for V136) as the suppression target site, with all other valine codons mutated to alanine codons. We first expressed the *folA* variant with the PURE system. Subsequent separation of the *in vitro* protein synthesis reaction by SDS-PAGE revealed a gel band corresponding to the expected molecular weight of the dihydrofolate reductase (DHFR) variant (18 kDa), the gene product of *folA* (Figure 4B). The band was absent if no DNA template was included in the PURE reaction. Next, we expressed the *folA* variant with the PURE system in the presence of either HisS alone or HisS with pre-aminoacylated mini HisT. In both conditions, gel bands indicative of DHFR variants were observed.

These gel bands were excised and used for peptide mass fingerprinting to detect if histidine was incorporated at the target position 136 of the DHFR variant (Figure 4C; Supplementary Figures S26-S29 and Supplementary Table S9). In the absence of mini HisT (but in the presence of HisS) during the PURE reaction, only valine was detected in peptides containing DHFR residue 136. However, in the presence of both mini HisT and HisS, both valine and histidine were detected as residue 136 in DHFR. The target residue 136 was encoded as a GTA codon and therefore decoded as valine according to the classical genetic code as observed in the control without mini HisT. When mini HisT was included in the PURE reaction, we observed that the GTA codon was also read out as histidine and thereby divergent to the classical genetic code. This demonstrates that mini HisT is active as a suppressor tRNA in an *in vitro* translation system. Given that the PURE system encompasses the *E. coli* translation apparatus, which has not been adapted or evolved to accommodate a D-arm-less tRNA fold, the result confirmed our *in vivo* observations: an artificially non-canonical tRNA fold, which lacks the entire D-arm, can be accepted *ab initio* by the involved parts of the *E. coli* translation machinery without any adaptation.

## Discussion

In this study, we explored whether structurally non-canonical tRNAs divergent from all known cytosolic tRNAs could be introduced in bacterial translation. We assumed that adaptations in the host cells are necessary to use foreign adaptors of the genetic code. Therefore, we crafted a genetic suppression system for the evolution of *E. coli* to utilize non-canonical tRNAs. The system couples the suppressor activity of HisT_UAC_ and the aminoacylation activity of HisS to the growth of a thiamine auxotrophic Sel_aux_ strain. The coupling was established by the suppression of an engineered target codon for V95 in the mRNA of *thiN*:H95V and results in the incorporation of an essential histidine in ThiN, which is required to complement the auxotrophy. The novelty of our vitamin-based system, compared to previously reported systems utilizing alternative metabolic suppression targets, lies in the low requirement for productive suppression events needed to confer growth. The addition of ∼1000 thiamine molecules per cell was sufficient to achieve full non-thiamine limited growth in selective medium. Consequently, we reasoned that fewer ThiN molecules in a cell are required to confer thiamine prototrophy compared to using an enzyme involved in, for example primary carbon metabolism, to complement an auxotrophy. This should allow for growth already at lower activity levels of the HisS/HisT pair. In addition, no metabolic escapees were observed in long-term incubations of strains containing incomplete systems. This reliability is especially remarkable considering that the thiamine auxotrophic phenotype is based on the change of a single codon and how little thiamine a potential escape pathway would have to produce for growth.

The developed ThiN-based suppression system was then exploited for the adaptation of *E. coli* to utilize structurally non-canonical tRNAs in translation. In principle, this is done by replacing a canonical component of the system (full-length HisT_UAC_) with a non-canonical component (HisT_UAC_-derived novel tRNA fold) and subsequently selecting for growth in long-term evolution experiments. Of all the non-canonical tRNA folds tested here, we succeeded in obtaining a growing strain for the mini HisT design lacking the entire D-arm. The lack of success for all other tested designs could have resulted from an intrinsic instability of the tRNA folds, a lack of acceptance by the translation machinery, or in general the necessity for multiple adaptations beyond those acquired during the experiment (e.g. mutations in multiple RNases). The suppression system operates under an all-or-nothing principle, meaning that all different reasons lead to the same negative result in the experiment. No additional information as to why no growth was achieved can be collected, which is a drawback of the adaptive evolution approach.

In this work, we developed a tight selection system in order to mobilize 12 non-canonical tRNA designs in *E. coli*. These correspond to removing one or multiple loops or entire arms from the tRNA cloverleaf. The same selection system could also be applied to screen large non-canonical tRNA libraries for discovering unprecedented folds active in translation. For this, regions of a histidine tRNA or an entire tRNA sequence could be randomized and the resulting libraries could be subjected to thiamine selection. The system is not limited to tRNAs but could be used further to adapt *E. coli* to utilize other non-canonical components involved in translation. It is especially suitable for components without any known initial cellular activity as demonstrated for mini HisT. Currently, our efforts are focused on evolving *E. coli* to utilize an artificial ribozyme (flexizyme), which so far is exclusively used *in vitro* for the aminoacylation of tRNAs, by replacing HisS with it in the system (39).

**Figure 5.**
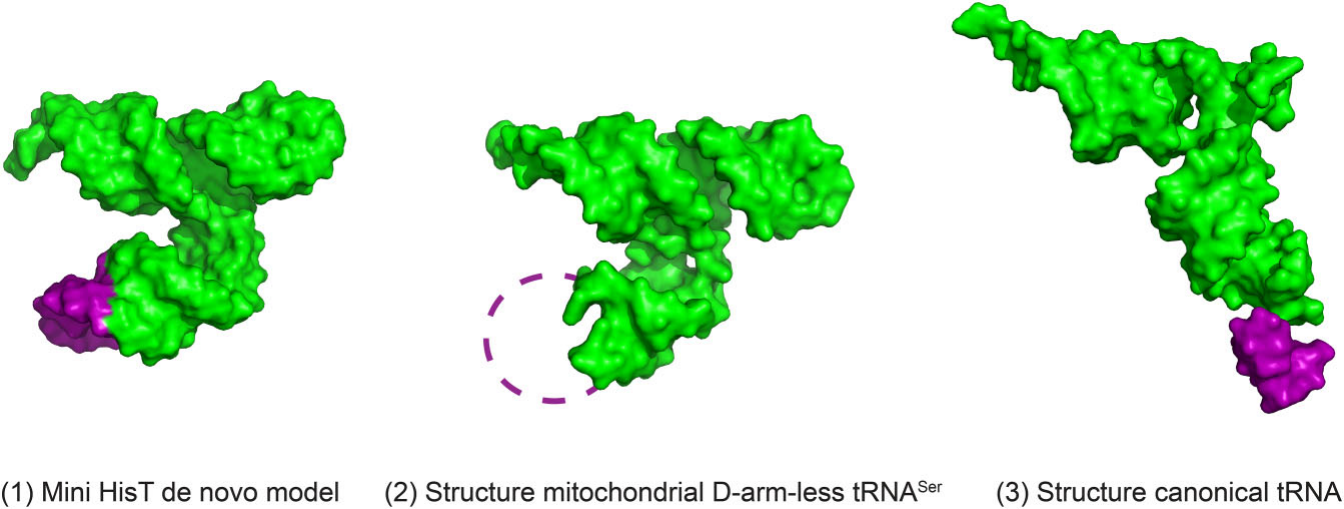
Model of mini HisT in comparison to experimentally determined tRNA structures. (1) Y-shaped *de novo* FARFAR2 model of mini HisT with anticodon loop in purple. (2) Cryo-EM structure of the Y-shaped human mitochondrial D-arm-less tRNA^Ser^ variant (PDB ID: 7U2B). The region of the nonresolved anticodon loop is illustrated by the purple dashed line. (3) Structure of a canonical L-shaped *E. coli* phenylalanyl tRNA (PDB ID: 6Y3G) with anticodon loop in purple.

All previously known cytosolic tRNAs fold into a cloverleaf secondary structure with the accompanying L-shaped tertiary structure, which cannot be adopted by mini HisT. To the best of our knowledge, mini HisT thus represents the first time a tRNA with an entirely different fold was shown to be utilized in the translation process of a living bacterium and in cytosolic translation in general. Natural arm-less tRNAs exist exclusively in mitochondria. Recently, the first cryo-EM structure of the human mitochondrial D-arm-less tRNA^Ser^ variant bound to the cognate SerRS was reported (40). In this natural tRNA and in mini HisT, the acceptor stem and the anticodon arm are connected by a stretch of 5 or 4 nucleotides, respectively. A 3D model of mini HisT was predicted de novo using FARFAR2 (Figure 5) (30,31). In comparison to the cryo-EM structure of the human mitochondrial D-arm-less tRNA^Ser^, the overall shape of the mini HisT model is strikingly similar. Both fold into a Y-shape, in which the T-arm is strongly reshaped due to the missing interaction with the D-arm. This indicates a high similarity between a natural D-arm-less tRNA, which originated from a natural drift of the fold, and the synthetic mini HisT.

To our surprise, the only adaptation required to utilize mini HisT in *E. coli* is a point mutation in the *pnp* gene coding for an RNase, which is most likely involved in the degradation of mini HisT. This indicates that HisS and the involved parts of the *E. coli* translation machinery (EF-Tu and the ribosome) accept mini HisT *ab initio* and we verified this hypothesis genetically and biochemically. Our work therefore demonstrates that the bacterial translation machinery is flexible to accommodate drastic alterations of the tRNA fold. The mobilization of a non-canonical tRNA lacking the D-arm could now be the basis for generating an oARS specific for the simplified fold by *in vivo* selection. By this, a novel kind of otRNA/ARS pairs could be obtained, whose orthogonality stems from the specificity of the oARS toward the alternative tRNA fold, rejecting L-shaped tRNAs.

In mitochondria, the translation machinery could co-evolve to accommodate non-canonical tRNA folds, but the fold of mini HisT is foreign to the translation machinery of the *E. coli* host cell and to the cognate HisS. Nevertheless, our results show that mini HisT is aminoacylated by HisS. The human mitochondrial SerRS evolved to recognize a non-canonical Y-shaped tRNA and a canonical L-shaped tRNA using two divergent binding modes, which could also be feasible for HisS (41). Interestingly, the ability of cytosolic ARS to accept tRNAs lacking the D-arm seems to be somewhat prevalent since it was previously reported that the yeast AspRS is active on an artificial D-arm-less tRNA (42). Furthermore, mini HisT acted as a functional suppressor tRNA during *in vitro* translation without any required adaptation in the translation machinery. Therefore, mini HisT is accepted by EF-Tu and the ribosome, which is especially interesting since the geometry of the Y-shape differs strongly from the canonical L-shaped tRNA, resulting in an altered distance between the attached amino acid and the anticodon (roughly half of the 7.5 nm in a canonical tRNA). It should be noted that mini HisT does not necessarily adopt the presumed Y-shape while bound to EF-Tu, HisS, or the ribosome. Due to the missing interaction between the D-arm and the T-arm, mini HisT is likely more flexible than a canonical tRNA. The binding to an interaction partner could induce a conformational change in mini HisT and, by that, increase the distance between the amino acid attachment site and the anticodon.

We observed here that mini HisT-dependent growth is slower under nonselective conditions compared to selective conditions, which is not the case for full length HisT. This indicates that mini HisT can either be inefficiently used for aminoacylation/translation or is intrinsically unstable, which was previously reported for mitochondrial D-arm-less tRNAs (40,43). We also observed that strains, which rely on a *mini hisT_TAC_* containing suppression system, do not grow at 37°C consistent with a low stability of mini HisT. Therefore, it would be reasonable to first stabilize mini HisT through a directed evolution campaign toward improved stability using selection at, for example, higher temperature or other methodologies. In future endeavors, (an improved) mini HisT could then be deployed in genetic takeover experiments. In these, one could attempt to completely replace the endogenous tRNA^His^ to explore if a non-canonical tRNA fold cannot only be productively used *in vivo* but also take over the cellular function of a canonical tRNA entirely.

Taken together, we developed a reliable and highly sensitive vitamin-based suppression system. It was successfully exploited to evolve *E. coli* to utilize the D-arm-less tRNA mini HisT in translation, which requires a single point mutation in *pnp*. At the beginning of the project, no information on PNPase-based degradation preventing the utilization of a D-arm-less tRNA was available and we had no clear indication to target the *pnp* gene in a rational approach to deploy mini HisT. The success of the evolution experiment thus illustrates the power of the vitamin-based selection approach and the ThiN-based suppression system to achieve cellular activity for a non-canonical adaptor of the genetic code. Our study demonstrates that the canonical structure of tRNA, which is invariably conserved in tRNA molecules across all known bacteria, can be drastically reshaped and still remain functional in translation. This reveals a previously unknown flexibility of the bacterial translation machinery and suggests that what we are accustomed to recognizing as immutably fixed in living organisms corresponds to only a fraction of what is possible to assemble and operate biomolecular machines.

## Supplementary Material

gkae806_Supplemental_File

## Data Availability

Illumina reads have been deposited to the Sequence Read Archive with the BioProject (https://www.ncbi.nlm.nih.gov/bioproject) accession number PRJNA1099589. The mini HisT model is available in ModelArchive: https://www.modelarchive.org/doi/10.5452/ma-41wpu. The mass spectrometry proteomics data have been deposited to the ProteomeXchange Consortium via the PRIDE (http://www.ebi.ac.uk/pride) partner repository with the data set identifier PXD051338. Assembled genomes have been deposited to the Sequence Read Archive with the BioProject accession number PRJNA1099634: Sel_aux_https://www.ncbi.nlm.nih.gov/nuccore/CP152469 Sel_aux_* https://www.ncbi.nlm.nih.gov/nuccore/CP152467 Sel(hit) https://www.ncbi.nlm.nih.gov/nuccore/CP152468 Sel^0^_hit_* https://www.ncbi.nlm.nih.gov/nuccore/CP152465 Sel_eng_pnp-mut_https://www.ncbi.nlm.nih.gov/nuccore/CP152466
