# Supplementary material for: Evolving Escherichia coli to use a tRNA with a non-canonical fold as an adaptor of the genetic code
Source: Nucleic Acids Res. 2024 Sep 24;52(20):12650–68. doi: 10.1093/nar/gkae806 (PMC11551756; doi:10.1093/nar/gkae806)
Supplement: gkae806_Supplemental_File [file gkae806_supplemental_file.pdf]

Supplementary information

Evolving *Escherichia coli* to use a tRNA with a non-canonical fold as an adaptor of the genetic code

Martin P. Edelmann, Sietse Couperus, Emilio Rodríguez-Robles, Julie Rivollier, Tania M. Roberts, Sven Panke*, Philippe Marlière*

* Correspondence should be addressed to Sven Panke. Tel: +41613873209; Email: sven.panke@bsse.ethz.ch. Correspondence may also be addressed to Philippe Marlière. Email: phmarliere@tesssi.eu.

This file includes:

Supplementary text 1-2

Supplementary figures 1-29

Supplementary tables 1-9

Supplementary sequences of selected plasmids

Supplementary references

Supplementary text

Supplementary text 1, Optimization of selective media conditions

The thiamine auxotrophic Sel_{aux} transformed with the complete suppression system grew in selective M9(glu) liquid medium (Supplementary Fig. 5). However, on M9(glu) agar plates most streaked cells of this strain did not show growth and for the observed colonies an impaired phenotype was detected. On the one hand, these results indicated that the suppression system worked as planned, and thiamine was produced intracellularly by the suppression system. On the other hand, the growth impairment indicated insufficient thiamine production even in the presence of the full suppression system. To resolve this issue, we looked for metabolic pathways that involve thiamine-containing enzymes in *E. coli*. In the case of a thiamine shortage in a cell, some thiamine-dependent enzymes will lack their cofactor (depending on expression levels and enzyme-cofactor binding affinities). This would result in an insufficient supply of the products of the reactions catalyzed by these enzymes. Intermediates downstream of the thiamine-dependent step in the respective pathways were supplemented to M9(glu) plates to identify the limiting metabolite(s) (Supplementary table 6) and the supplementation of pantothenate (pan) to M9(glu) indeed resulted in a normal growth phenotype for the observed colonies (Supplementary Fig. 6).

We reasoned that a lower thiamine requirement for Sel_{aux} would be beneficial to increase the sensitivity of the suppression system and performed a second optimization step to achieve this. If the strain needs less extracellular thiamine to grow in the absence of the system, it should require fewer active ThiN molecules per cell for growth in the presence of the system (in the absence of extracellular thiamine). This would increase the sensitivity of the system since fewer suppression events would be sufficient for growth. Using the rationale explained in the last paragraph, we changed the selective medium composition by supplementing the metabolites identified there (Supplementary table 6). We supplemented all metabolites together to the medium under thiamine-limited conditions and indeed obtained an increased growth yield for Sel_{aux} (Supplementary Fig. 7). The same effect was achieved when only pan, leucine/valine/isoleucine (ILV) and succinate (suc) were supplemented together under thiamine-limited conditions. The resulting medium without thiamine was termed $M(glu)^{sup}$. To test the success of the medium optimization, growth curves of Sel_{aux} in M9(glu) medium supplemented with only 2 µM pan and in $M9(glu)^{sup}$ medium with the addition of different concentrations of thiamine were recorded (Supplementary Fig. 8). In $M9(glu)^{sup}$ higher growth yields were measured compared to M9(glu) medium with 2 µM pan for the same concentrations of thiamine.

Supplementary text 2, Technical reasons for low peptide counts and reliability of results

The number of counts per peptide in the peptide mass fingerprinting experiment is lower than observed in some similar experiments and there are a number of technical reasons for this. We used Asp-N instead of standard trypsin for digestion. Given the specific primary sequence of the modified DHFR, this leads to a beneficial predicted digestion pattern, in particular around the critical amino acid position 136, in comparison to trypsin: One can expect peptide fragments in the proper size range for MS/MS. The downside of using Asp-N is that many obtained peptides lack a basic amino acid residue, which makes the spectral assignment more difficult in positive-mode MS. For trypsin, positively charged residues are always present at the C-termini due to its cleavage pattern. This has an advantage for the assignment since MS/MS ions from both ends can be detected. Hence, the counts for high quality peptide spectral matches (PSMs), which can be assigned unambiguously, tend to be lower in our Asp-N-based experiments despite the latest MS instrumentation. Furthermore, the work-up (extraction of peptides from excised gel band) can lead to the loss of peptides, and the complex sample leads to a high background (peptides stemming from multiple proteins of the PURE mix were detected) compared to a sample of a purified protein.

Depending on the sample, we were able to cover 55-84% of the entire DHFR variant sequence via peptide mass fingerprinting. It should be noted that closer inspection of the raw data shows that a reduced number of counts applies consistently to the peptides originating from DHFR after Asp-N digest, not only to the PSMs in question containing residue 136. This indicates that these peptide counts are not outliers in comparison to the PSM detected for the residual sequence. The peptide-fingerprinting-experiment was performed twice here to ensure the observed results are reproducible. In total, two replicates for the suppression sample and two replicates for the negative control were independently analyzed (pooled and individual results in Supplementary table 9). Not only did we reproduce the same results - only peptides with V136 for negative control, mix of peptides with V136 and H136 for suppression case - but we also measured similar amounts of counts throughout all replicates. This shows that there is no problem for any single sample, indicating that the general experimental reasons discussed above are responsible for the lower number of counts. Nevertheless, we identified multiple high quality PSMs containing residue 136 for all samples. These peptides showed an excellent identification probability (higher than 99%, in some cases even 100%, see Supplementary figures 26-29) and were only accepted for Fig. 4c using a very stringent false discovery rate (lower than 0.1%). This demonstrates that the PSMs found are not artifacts but are indicative of the functioning of mini-HisT in vitro and by that confirm our in vivo results.

Supplementary figures

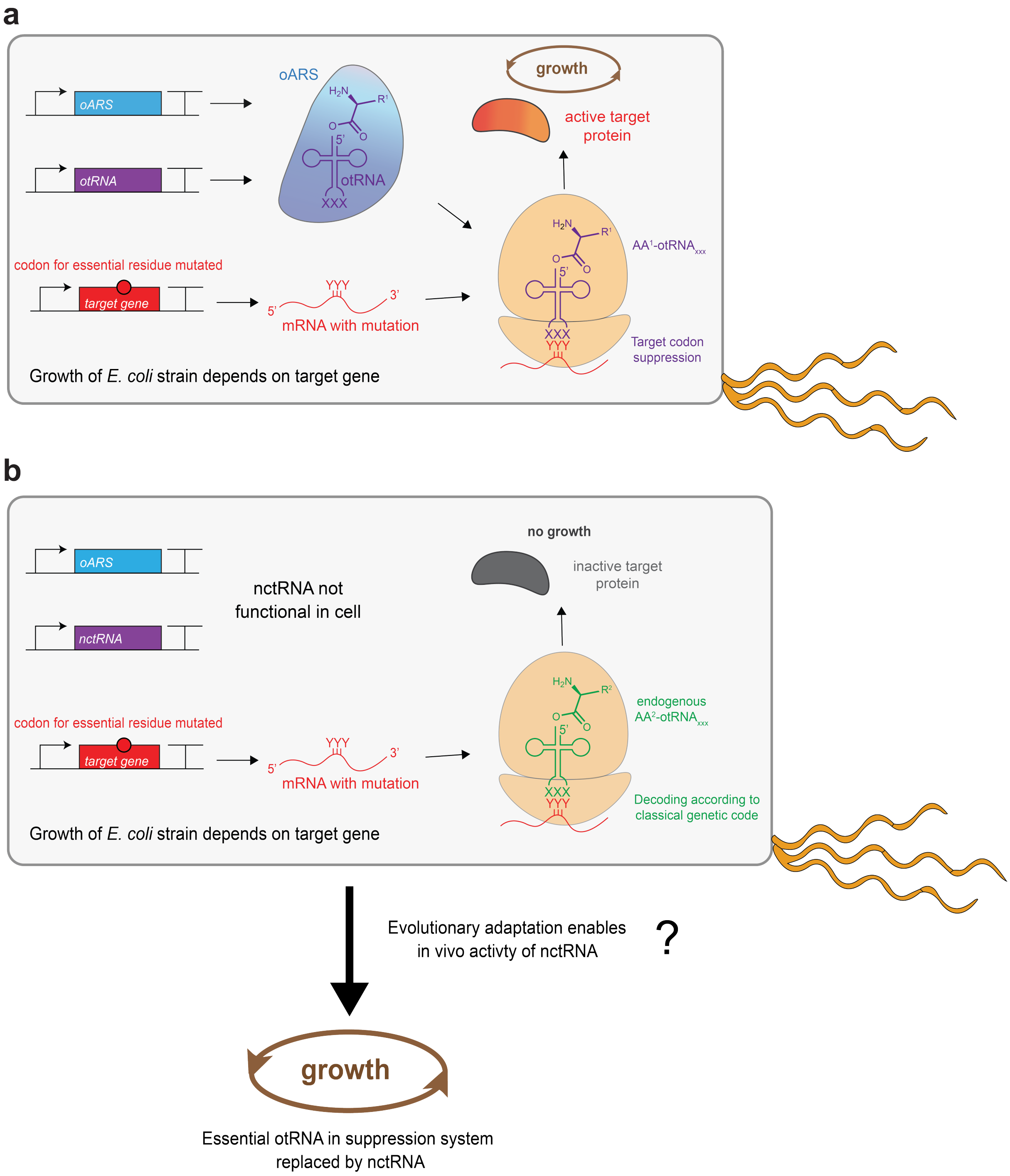

Supplementary fig. 1, a. A suppression system and the envisioned application. a. General concept of an essential suppression system. The genetic system metabolically couples the activity of an orthogonal tRNA (otRNA) and a cognate orthogonal aminoacyl tRNA synthetase (oARS) to the growth of an *E. coli* strain. The codon of an essential residue in a target gene is mutated to render the gene product inactive. An otRNA/oARS pair is selected to incorporate the original required residue for activity and the anticodon of the otRNA is changed to read out the mutated codon. Suppression of the mutated codon leads to the production of the active target protein that complements an essential pathway, which results in the growth of

the strain. **b. Using the essential suppression system for the evolution of *E. coli* towards activity of non-canonical tRNAs.** The essential otRNA in the system is replaced by a non-canonical tRNA (nctRNA) that is initially not functional in the host cell. Therefore, the mutated codon of the essential residue in the suppression target gene is exclusively decoded according to the classical genetic code. This results in the incorporation of an amino acid in the target protein that does not allow for activity and the host cell cannot grow. For an ideal system that cannot be bypassed, the cell is forced to evolve towards activity of the nctRNA to achieve suppression replacing the canonical otRNA and thus restoring growth.

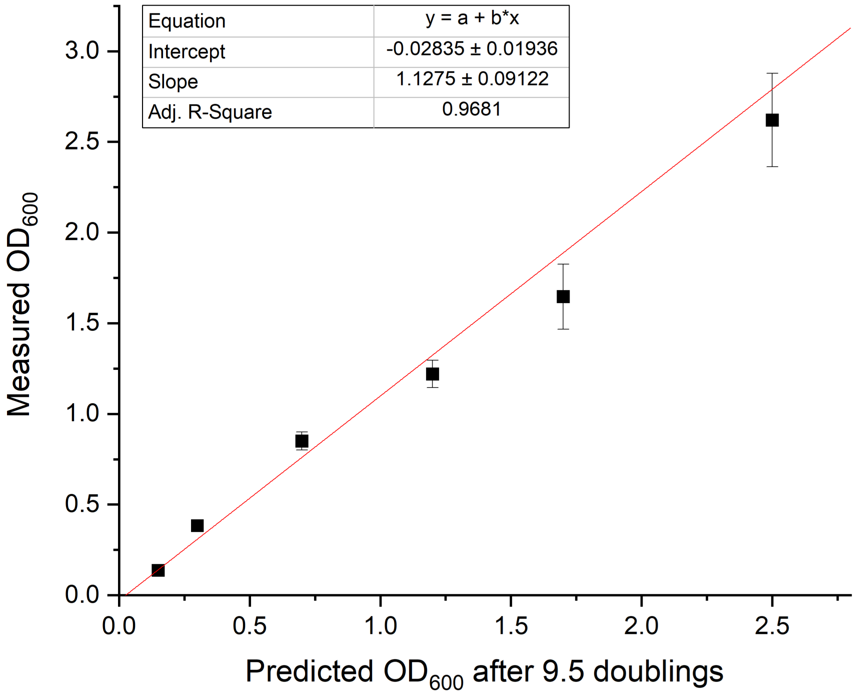

Supplementary fig. 2, Thiamine auxotrophic strain Sel_{aux} can perform ~ 9.5 doublings in selective M9(glu)^{sup} medium due to intracellularly stored thiamine. M9(glu)^{sup} cultures were inoculated using Sel_{aux} cells from an overnight preculture (in M9(glu)^{sup} medium + 10 µM thiamine) that were washed four times using M9(glu)^{sup} medium as described in Materials and Methods. The initial starting OD_{600} of the cultures was adjusted to obtain an OD_{600} of 0.15, 0.3, 0.7, 1.2, 1.7, or 2.5 after 9.5 doublings. After 36 h, the OD_{600} of each culture was measured and plotted against the predicted OD_{600}. The experiment was performed in biological triplicates. The means ± 1 standard deviations are shown and a linear fit was performed using OriginPro 2021b (OriginLab Corporation).

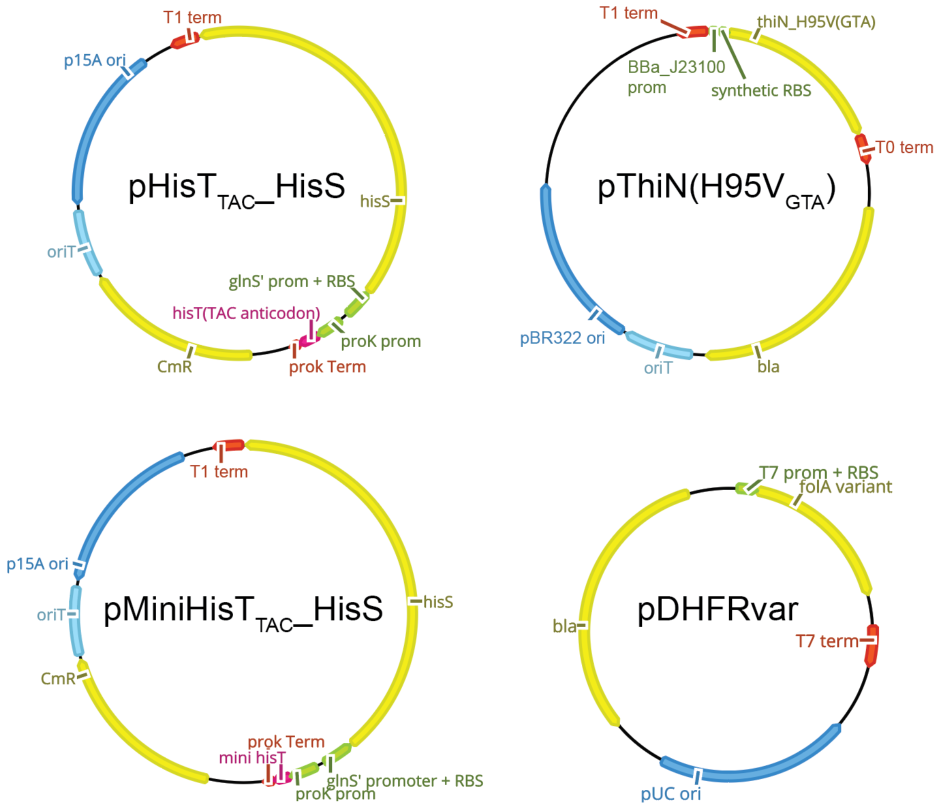

Supplementary fig. 3, Plasmid maps of selected plasmids. Abbreviations: T1 term = terminator of *rrnB* operon in *E. coli*, prom = promoter, RBS = ribosome binding site, CmR = chloramphenicol resistance gene, bla = carbenicillin resistance gene (beta-lactamase), T0 term = lambda phage terminator, T7 term = terminator T7 polymerase, ori = origin of replication and oriT = origin of transfer. Complete sequences see **Supplementary sequences**. Plasmid maps created with Geneious Prime 2022.

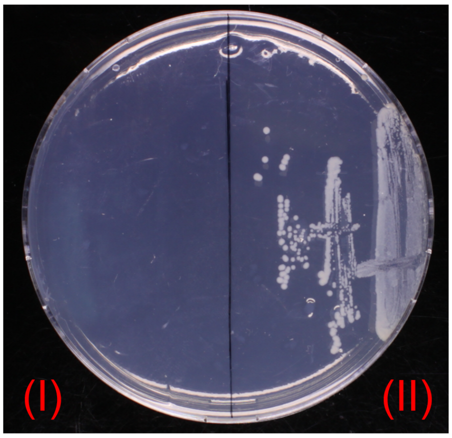

Supplementary fig. 4, Expression of wt *thiN* confers thiamine prototrophy to Sel_{aux}. The thiamine auxotrophic *E. coli* strain Sel_{aux} transformed with the following plasmids on M9(glu) agar plate after 3 d: (I) pSEVA191 (empty plasmid backbone); (II) pThiN. Cultivation of these strains under identical conditions was repeated three independent times obtaining the same result each time.

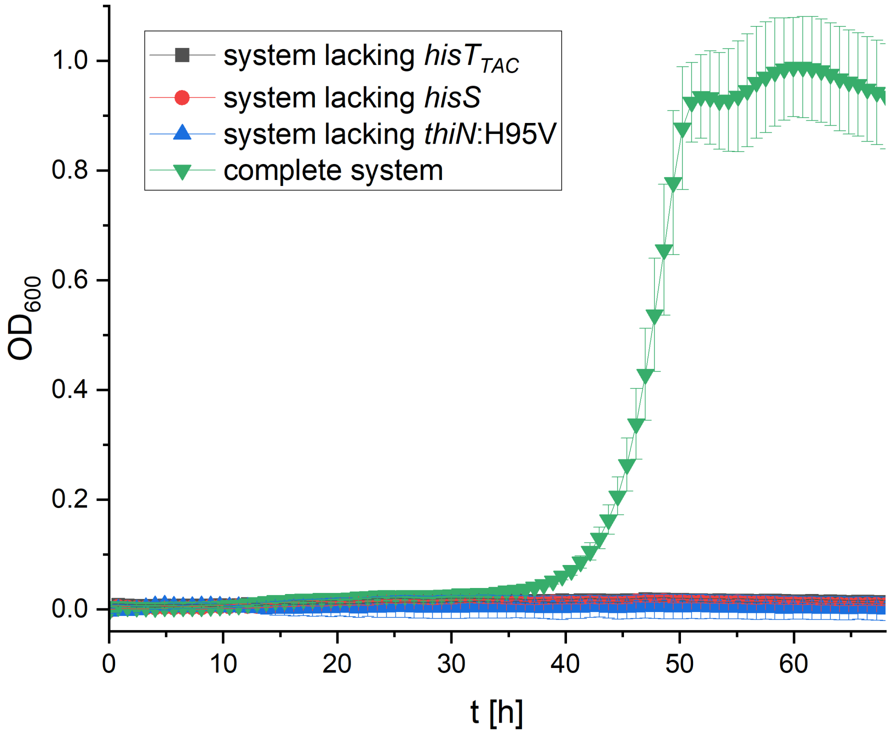

Supplementary fig. 5, All components of the ThiN-based suppression system are required to confer thiamine prototrophy to Sel_{aux}. The complete suppression system consists of the genes *hisS*, *thiN*:H95V and *hisT*$_{TAC}$ expressed in Sel_{aux}. Growth curves of Sel_{aux} with the complete or incomplete suppression systems, each of which lacked one component (see inset), in M9(glu) medium. The strains carried the following plasmids: (■) pHisS and pThiN(H95V$_{GTA}$); (●) pHisT$_{TAC}$ and pThiN(H95V$_{GTA}$); (▲) pHisS_HisT$_{TAC}$ and pSEVA191 (empty plasmid backbone); (▼) pHisS_HisT$_{TAC}$ and pThiN(H95V$_{GTA}$). The growth assay was performed in triplicates and the mean ± 1 standard deviation are shown.

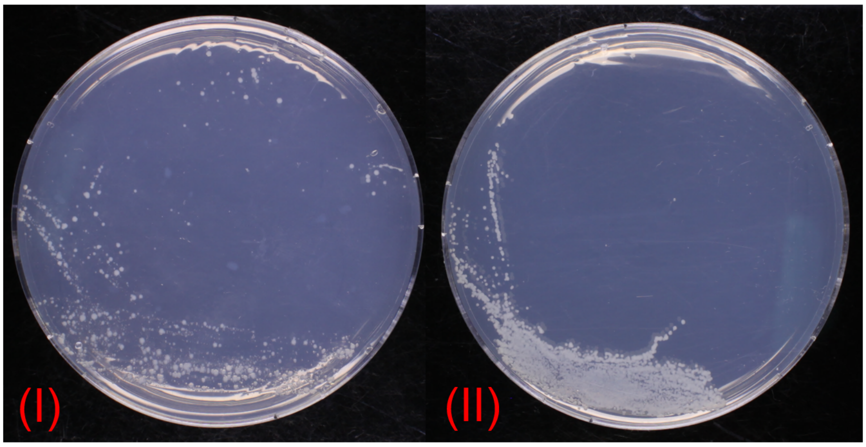

Supplementary fig. 6, Supplementation of pan results in improved phenotype for Sel$_{aux}$ containing the complete suppression system under selective conditions. Sel$_{aux}$ was transformed with the plasmid pHisS_HisT$_{TAC}$ and pThiN(H95V$_{GTA}$) to express *hisS*, *thiN*:H95V and *hisT$_{TAC}$* (complete suppression system). The resulting strains were incubated for 3 d on (I) M9(glu) and (II) M9(glu) + 2 µM pantothenate (pan) agar plates.

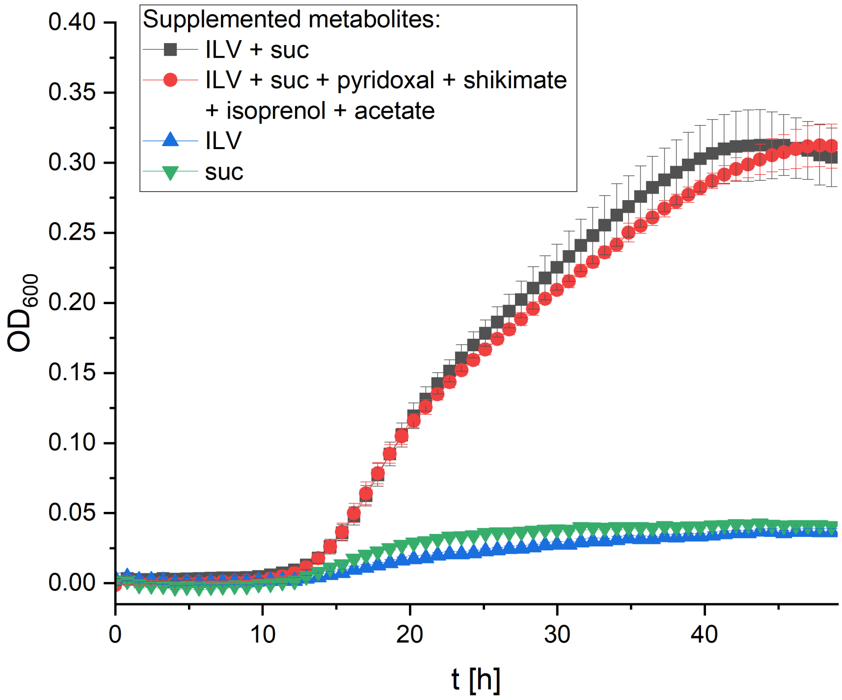

Supplementary fig. 7, The supplementation of isoleucine/leucin/valine (ILV) and succinate (suc) increases growth yields for Sel$_{aux}$ under thiamine limited conditions. Metabolites in pathways, which occur downstream of a thiamine-dependent step, were added to M9(glu) medium containing 2 µM pan and 0.25 nM thiamine. Growth curves of Sel$_{aux}$ were recorded in the following resulting media: (■) M9(glu) + 2 µM pan + 0.25 nM thiamine + 1 mM suc + 0.5 mM ILV; (●) M9(glu) + 2 µM pan + 0.25 nM thiamine + 1 mM suc + 0.5 mM ILV + 1 µM pyridoxal + 1 mM shikimate + 0.5 mM isoprenol + 10 mM acetate; (▲) M9(glu) + 2 µM pan + 0.25 nM thiamine + 0.5 mM ILV and (▼) M9(glu) + 2 µM pan + 0.25 nM thiamine + 1 mM suc. The growth assay was performed in triplicates and the mean ± 1 standard deviation are shown.

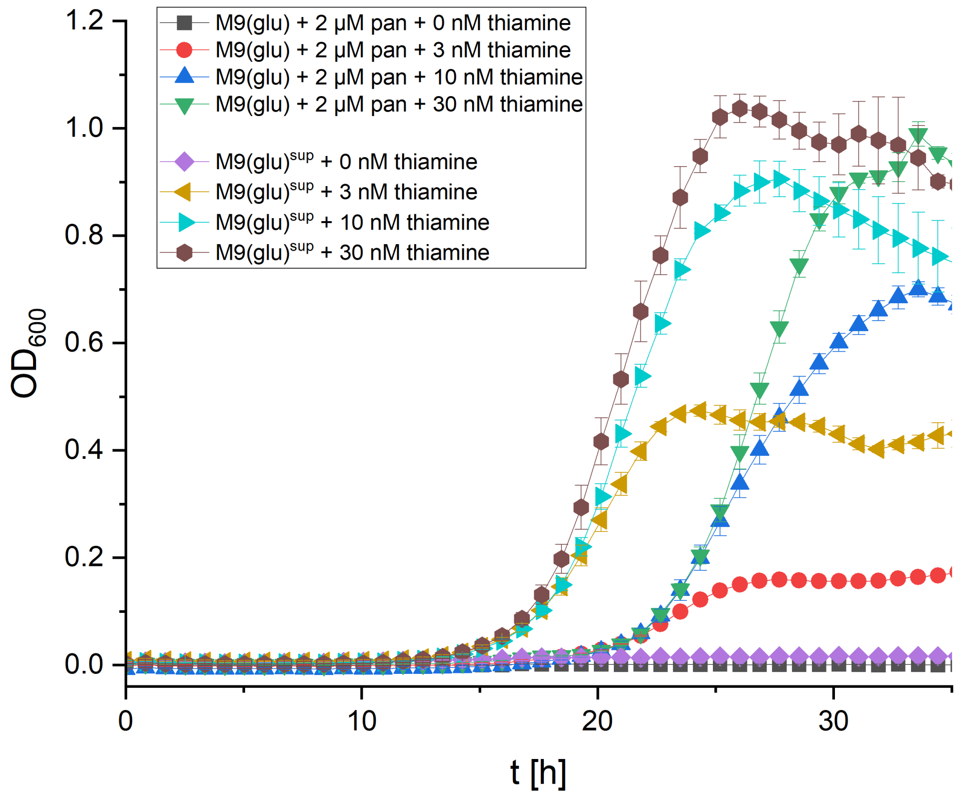

Supplementary fig. 8, Cultivation of Sel_{aux} in $M9(glu)^{sup}$ results in higher growth yields under thiamine-limited conditions compared to M9(glu) + pan. Growth curves of Sel_{aux} were recorded in M9(glu) medium + 2 µM pan and in $M9(glu)^{sup}$ medium supplemented with different concentrations of thiamine or no thiamine as a negative control. The growth assay was performed in triplicates and the mean ± 1 standard deviation are shown. For $M9(glu)^{sup}$ medium supplemented with 10 nM thiamine (~ $6*10^{12}$ molecules * ml^{-1}) an OD_{600} of nearly 1 was reached, which corresponds to ~ $5*10^{9}$ cells * ml^{-1} in the utilized plate reader device. Consequently, it is inferred that approximately 1000 molecules of thiamine are required per cell in the optimized medium.

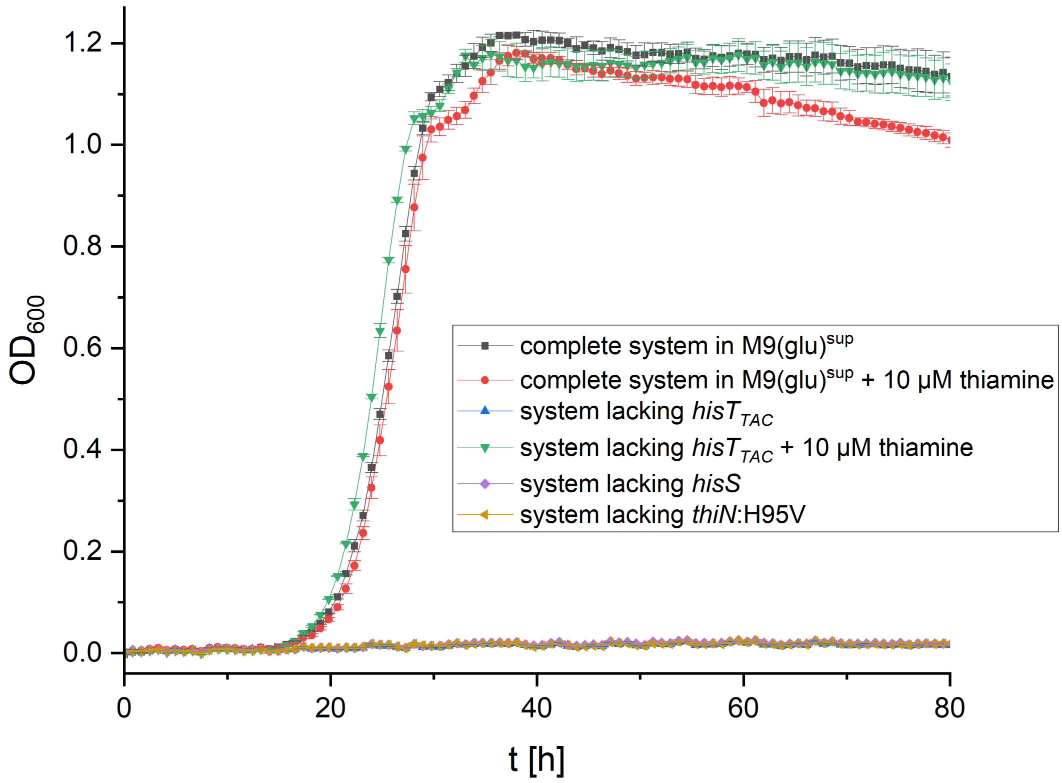

Supplementary fig. 9, All components of the suppression system are required to confer thiamine prototrophy to Sel_{aux} in $M9(glu)^{sup}$ medium. Growth curves of Sel_{aux} transformed with the complete suppression system or incomplete systems in $M9(glu)^{sup}$ medium without and with 10 µM thiamine (no-selective medium). (■) complete system: pHisS_$HisT_{TAC}$ and pThiN(H95V_{GTA}) without thiamine; (●) complete system: pHisS_$HisT_{TAC}$ and pThiN(H95V_{GTA}) with 10 µM thiamine; (▲) no *$hisT_{TAC}$*: pHisS and pThiN(H95V_{GTA}) without thiamine; (▼) no *$hisT_{TAC}$*: pHisS and pThiN(H95V_{GTA}) with 10 µM thiamine; (♦) no *hisS*: p$HisT_{TAC}$ and pThiN(H95V_{GTA}) without thiamine; (◄) no *thiN*: pHisS_$HisT_{TAC}$ and pSEVA191 without 10 µM thiamine. The growth assay was performed in triplicates and the mean ± 1 standard deviation are shown.

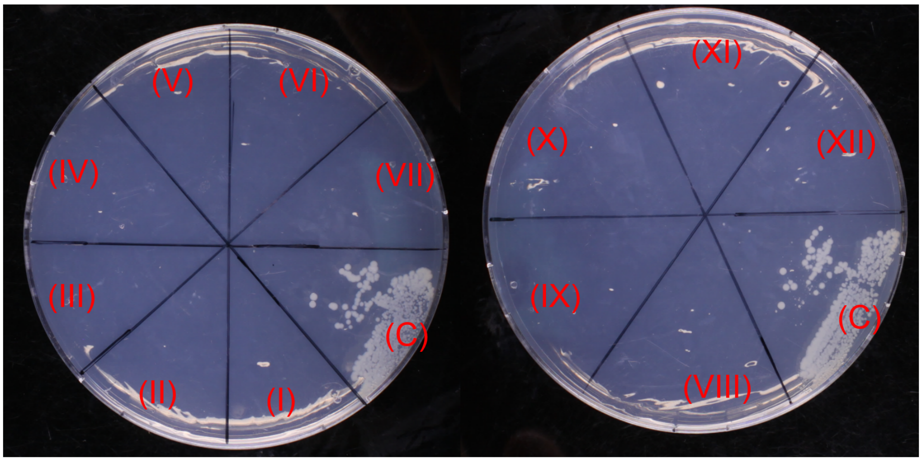

Supplementary fig. 10, Suppression systems containing non-canonical tRNA designs do not confer direct thiamine prototrophy. In the ThiN-based suppression system *hisT*$_{TAC}$ was replaced by non-canonical tRNA constructs. For this, Sel$_{aux}$ was transformed with pThiN(H95V$_{GTA}$) and one of the plasmids containing *hisS* and the non-canonical tRNA constructs: pHisT(I)_HisS, pHisT(II)_HisS, ..., pHisT(XII)_HisS. The resulting strains were named I to XII according to the non-canonical tRNA designs utilized. As a control (C) Sel$_{aux}$ was transformed with the complete suppression system containing full length *hisT*$_{TAC}$: pHisS_HisT$_{TAC}$ and pThiN(H95V$_{GTA}$). The strains were incubated for 7 d on M9(glu)^{sup} agar plates.

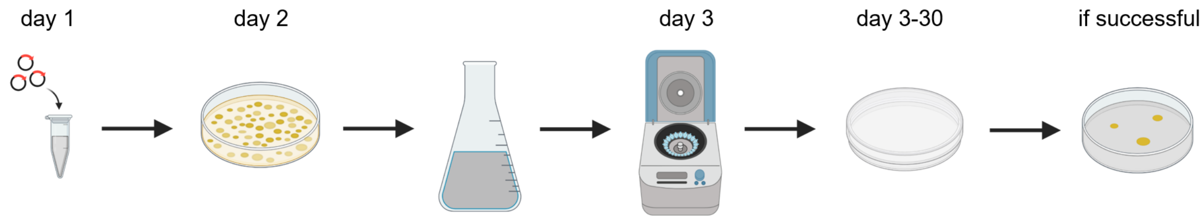

Supplementary fig. 11, Adaptive evolution experiment workflow to select for strains utilizing non-canonical tRNA designs in translation. Day 1: transformation of Sel$_{aux}$ with suppression systems containing non-canonical tRNA constructs, which encode for tRNAs that are initially not active. Day 2: Inoculation of non-selective M9(glu) medium supplemented with 10 µM thiamine for overnight incubation. Day 3: washing of cells to remove residual thiamine and streaking of cells on selective M9(glu)^{sup} agar plates. Day 3-30: incubation of plates for up to 30 d or until colonies appear. After a verification of the growth phenotype of the obtained strains, the evolutionary adaptations that resulted in growth can be identified by whole genome-sequencing. Figure created with Biorender.com.

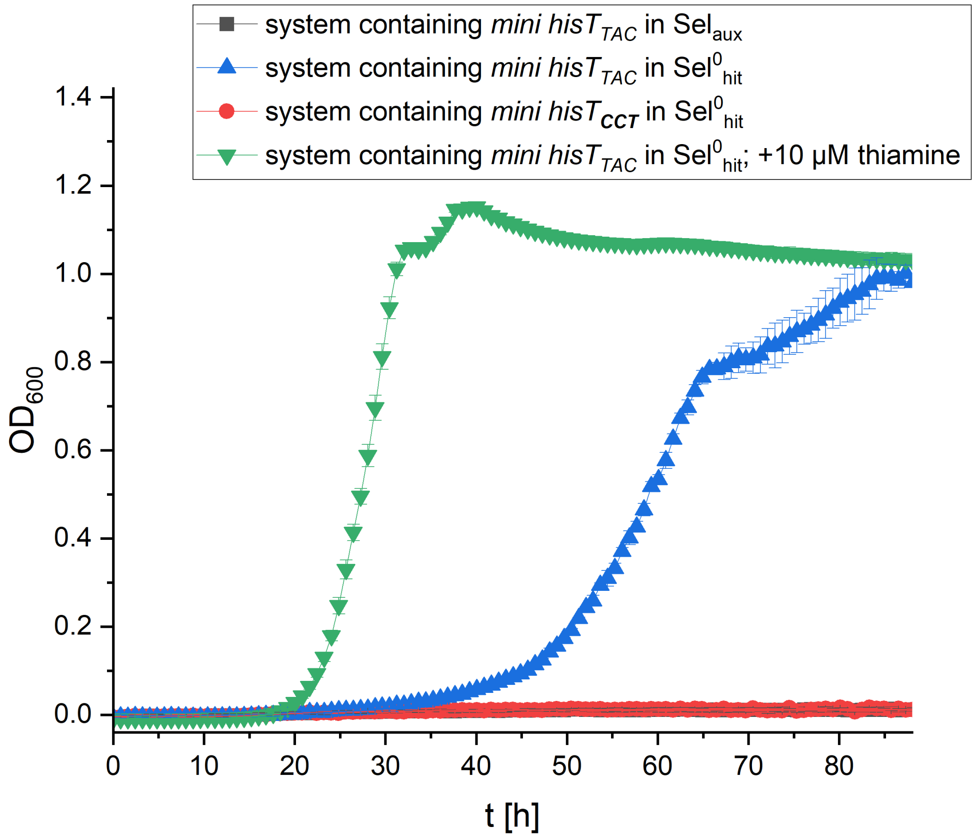

Supplementary fig. 12, Thiamine prototrophy conferred by a *mini hisT*$_{TAC}$ containing system is dependent on the correct mini HisT anticodon and requires the evolved strain. Growth curves in M9(glu)^{sup} medium. (■) Unevolved Sel$_{aux}$ transformed with *mini hisT$_{TAC}$* containing suppression system: pHisS_MiniHisT$_{TAC}$ and pThiN(H95V$_{GTA}$); (●) Evolved Sel$^{0}_{hit}$ retransformed with defective *mini hisT$_{CCT}$* containing suppression system (anticodon changed to avoid decoding of suppression target codon): pHisS_MiniHisT$_{CCT}$ and pThiN(H95V$_{GTA}$); (▲) Evolved Sel$^{0}_{hit}$ retransformed with *mini hisT$_{TAC}$* containing suppression system: pHisS_MiniHisT$_{TAC}$ and pThiN(H95V$_{GTA}$); (▼) Evolved Sel$^{0}_{hit}$ retransformed with *mini hisT$_{TAC}$* containing suppression system: pHisS_MiniHisT$_{TAC}$ and pThiN(H95V$_{GTA}$) with 10 μM thiamine in the medium (non-selective medium). The growth assay was performed in triplicates and the mean ± 1 standard deviation are shown.

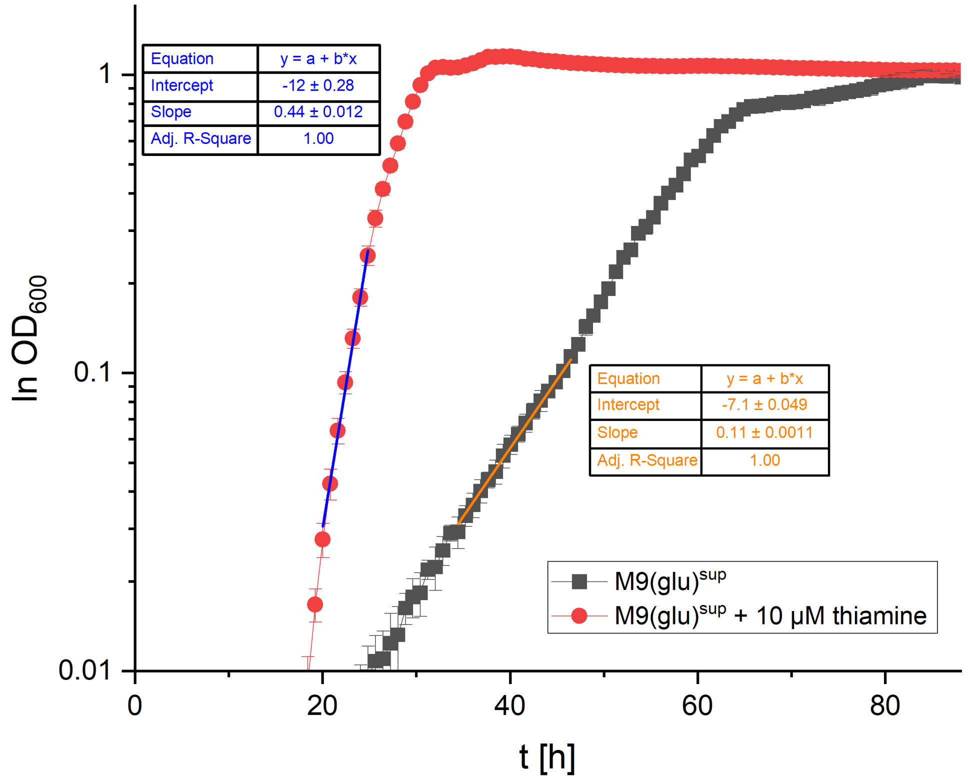

Supplementary fig. 13, The growth rate of evolved strain utilizing the *mini hisT_{TAC}* containing suppression system under selective conditions is reduced compared to non-selective conditions. Growth curves of Sel^{0}_{hit} retransformed with *mini* $hisT_{TAC}$ containing suppression system: pHisS_MiniHisT_{TAC} and pThiN(H95V_{GTA}) were recorded in (■): M9(glu)^{sup} and (●): M9(glu)^{sup} and 10 µM thiamine medium. The natural logarithm of the OD_{600} was plotted against the time. Linear regression curves were fitted in the exponential phase to determine μ_{max} as the slope of the respective curve. The growth assay was performed in triplicates and the mean ± 1 standard deviation are shown.

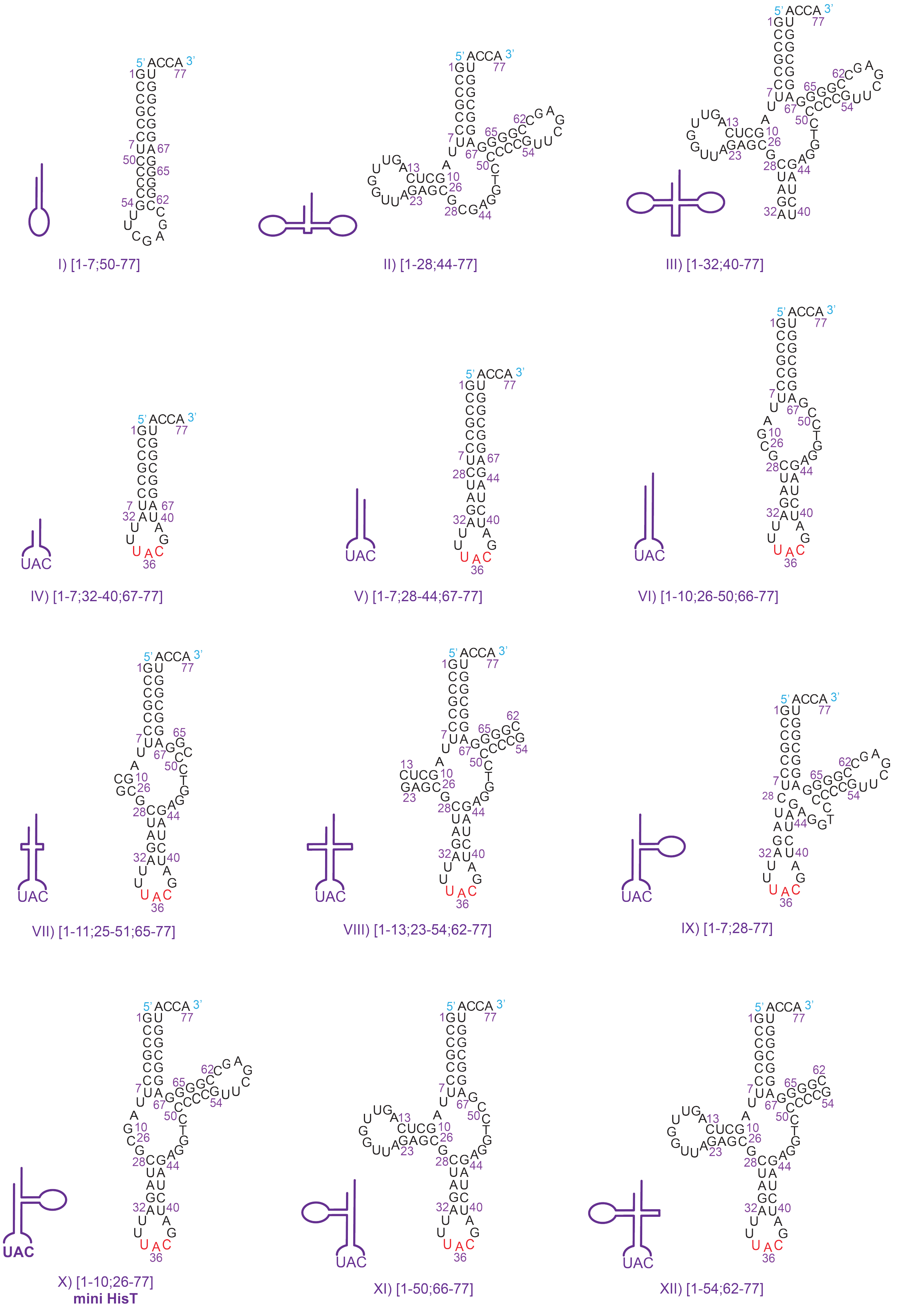

Supplementary fig. 14, 2D structures of non-canonical tRNA designs I-XII with sequences. The base numbering follows full length $HisT_{UAC}$ and the maintained nucleotides for each design are displayed in brackets. The anticodon is depicted in red.

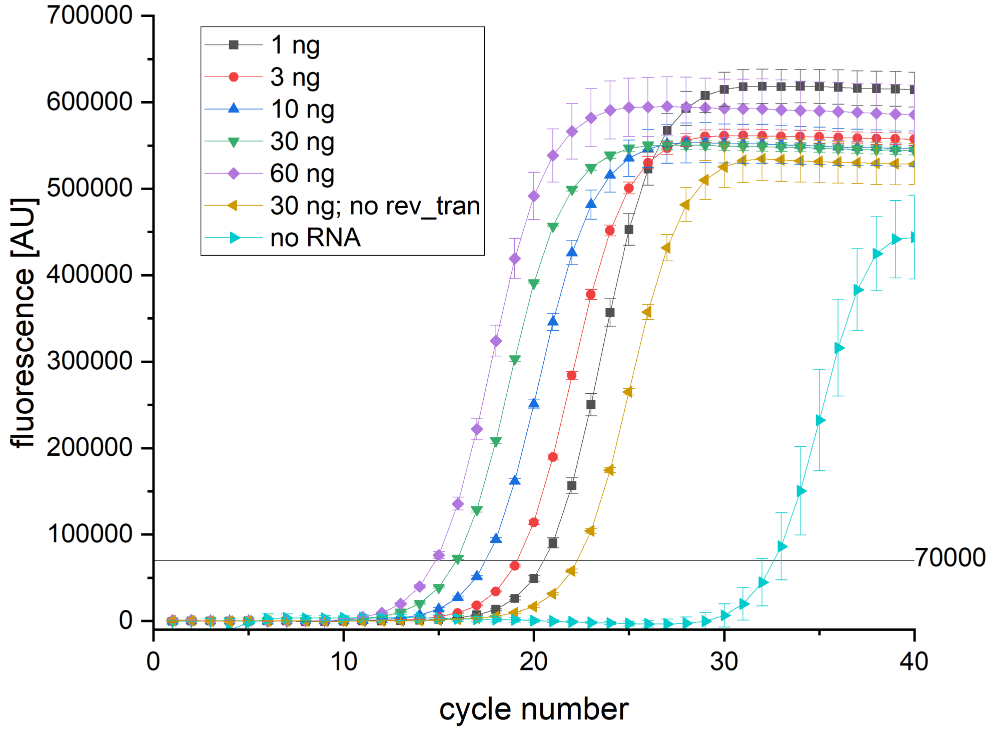

Supplementary fig. 15, qPCR amplification plots for *ihfB* reference as target at different template concentrations. Different amounts of total RNA extracted from a culture of Sel_{aux}* transformed with $pMiniHisT_{TAC}$_pur were used for the RT-qPCR reactions. Samples without any RNA or with 30 ng of RNA but without reverse transcription (no rev_tran) were used as negative controls. C_T values for each concentration were obtained at a fluorescence of 70,000. The RT-qPCRs were performed in triplicates and the mean ± 1 standard deviation are shown.

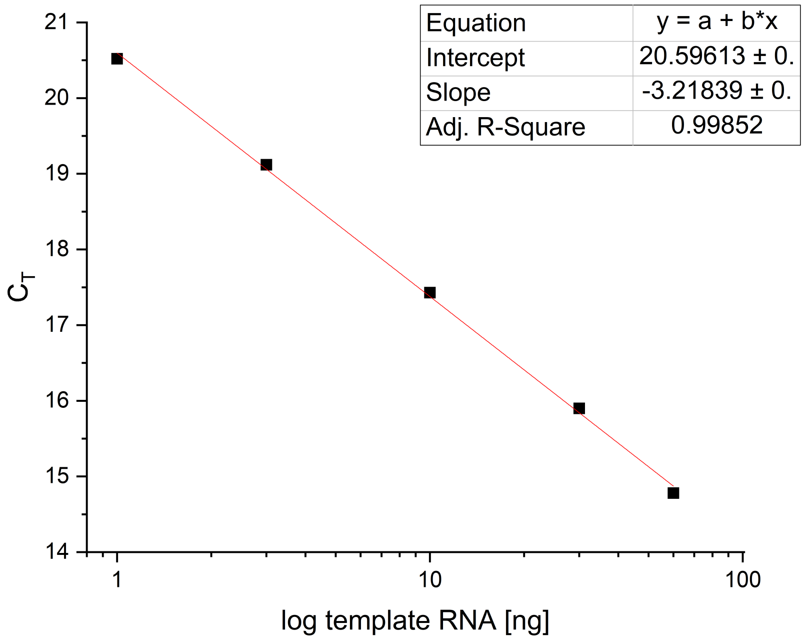

Supplementary fig. 16, qPCR standard curve for the *ihfB* reference as target demonstrates linearity. The mean of the C_T values (of technical triplicate) obtained from qPCR amplification plots in Supplementary fig. 15 were plotted against $\log_{10}$ of the used template (total RNA extracted from cells) concentration to perform a linear regression. qPCR efficiency = 105 %.

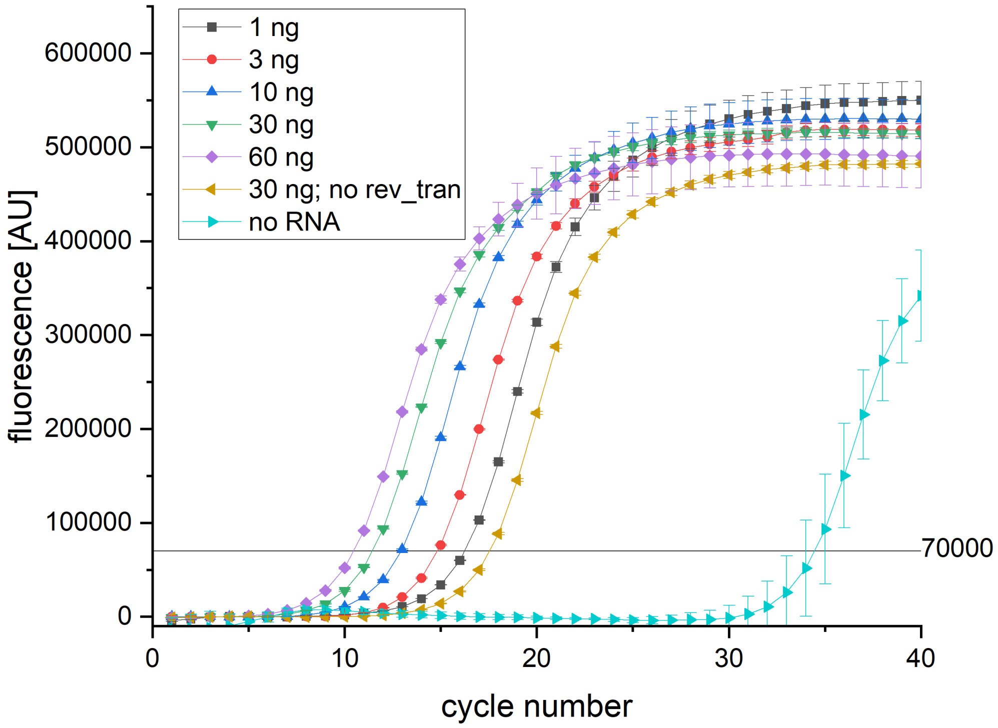

Supplementary fig. 17, qPCR amplification plots for mini HisT as target at different template concentrations. Different amounts of total RNA extracted from a culture of Sel_{aux}* transformed with $pMiniHisT_{TAC}$_pur were used for the RT-qPCR reactions. Samples without any RNA or with 30 ng of RNA but without reverse transcription (no rev_tran) were used as negative controls. C_T values for each concentration were obtained at a fluorescence of 70,000. The RT-qPCRs were performed in triplicates and the mean ± 1 standard deviation are shown.

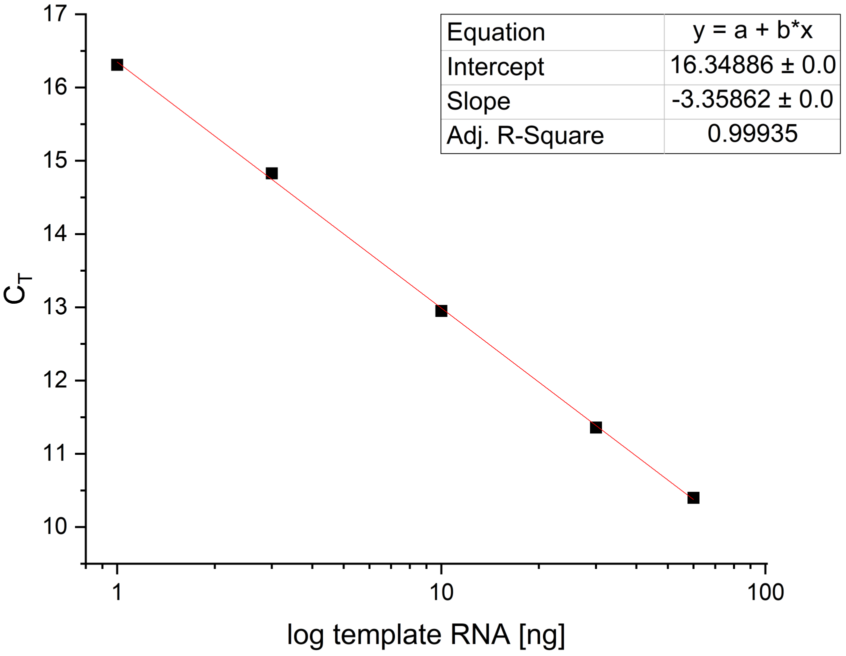

Supplementary fig. 18, qPCR standard curve for mini HisT as target demonstrates linearity. The mean of the C_T values (of technical triplicate) obtained from qPCR amplification plots in Supplementary fig. 17 were plotted against $\log_{10}$ of the used template (total RNA extracted from cells) concentration to perform a linear regression. qPCR efficiency = 98.52 %.

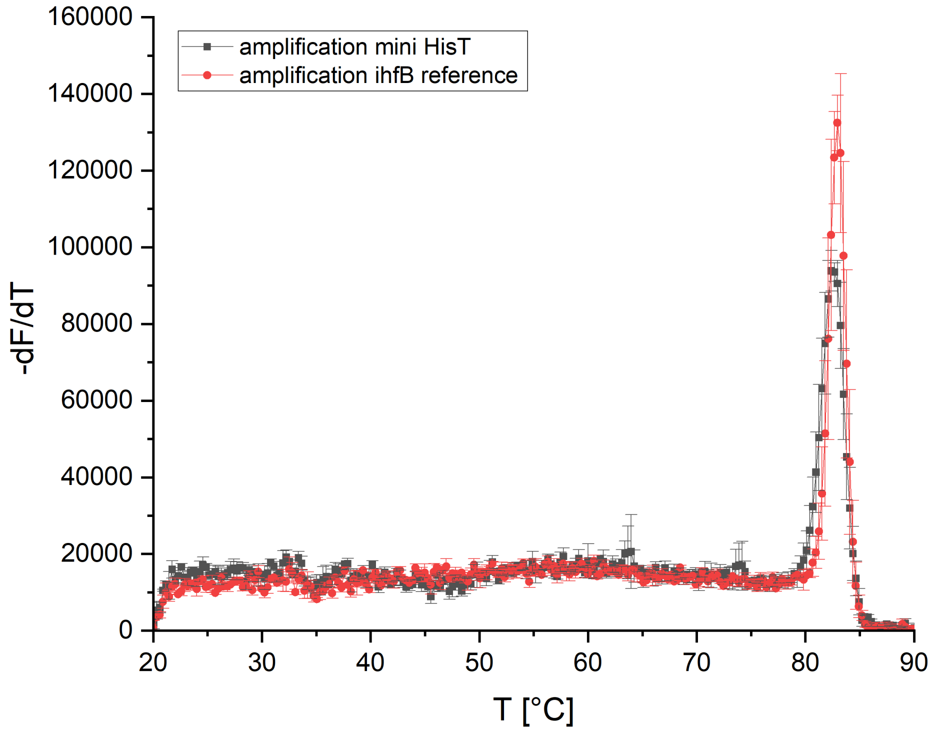

Supplementary fig. 19, Melting curves recorded after the RT-qPCR reactions with either mini HisT or the *ihfB* reference as the qPCR target show a single peak. The depicted graphs were recorded for 30 ng total RNA template. A absence of multiple peaks indicated the amplification of a single PCR product. The melting curves were recorded in triplicates and the mean ± 1 standard deviation are shown.

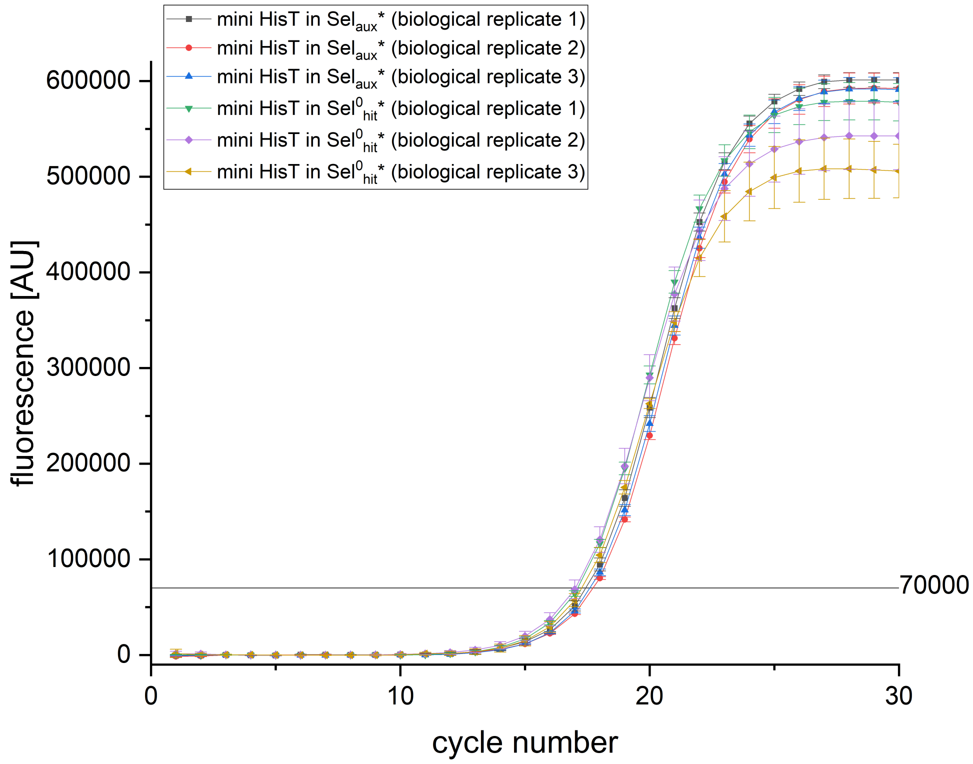

Supplementary fig. 20, qPCR amplification plots for the *ihfB* reference as the target in biological triplicates. Total RNA was extracted from either Sel_{aux}* (wt *pnp*) or Sel^{0}_{hit}* (*pnp*:G573S) each transformed with $pMiniHisT_{TAC}$_pur. 10 ng of total RNA template were used for each of the RT-qPCR reactions, which was in the linear range of the standard curves. C_T values for each replicate were obtained at a fluorescence of 70,000. The RT-qPCRs were performed in technical triplicates and the mean ± 1 standard deviation are shown.

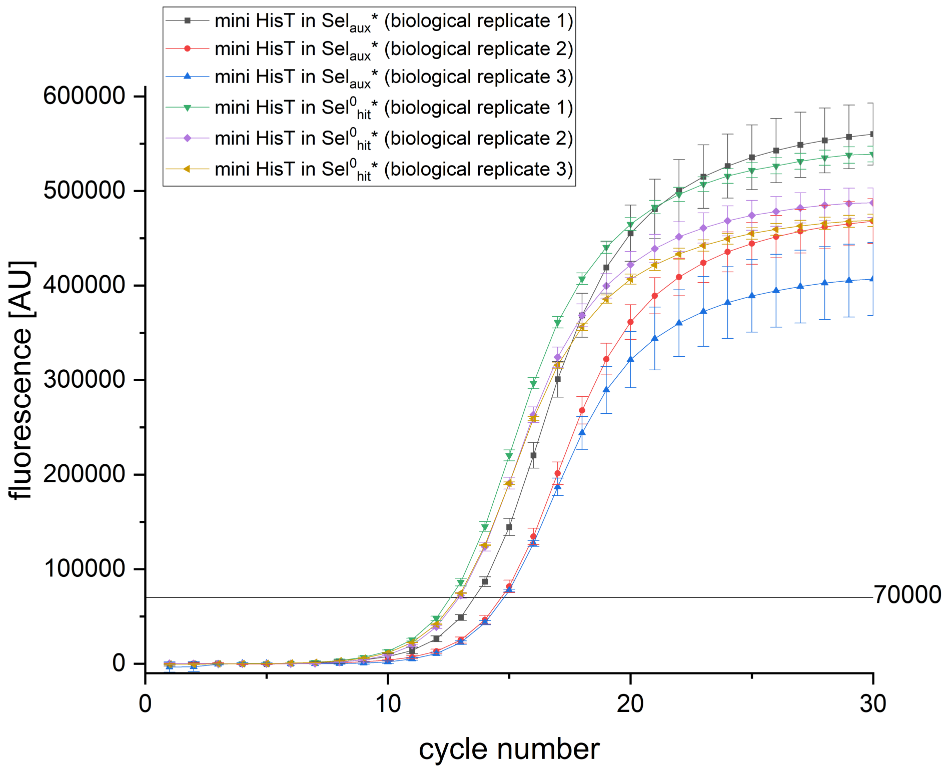

Supplementary fig. 21, qPCR amplification plots for mini HisT as the target in biological triplicates. Total RNA was extracted from either Sel$_{aux}$* (wt *pnp*) or Sel$^0_{hit}$* (*pnp*:G573S) each transformed with pMiniHisT$_{TAC}$_pur. 10 ng of total RNA template were used for each of the RT-qPCR reactions, which was in the linear range of the standard curves. C_T values for each replicate were obtained at a fluorescence of 70,000. The RT-qPCRs were performed in technical triplicates and the mean ± 1 standard deviation are shown.

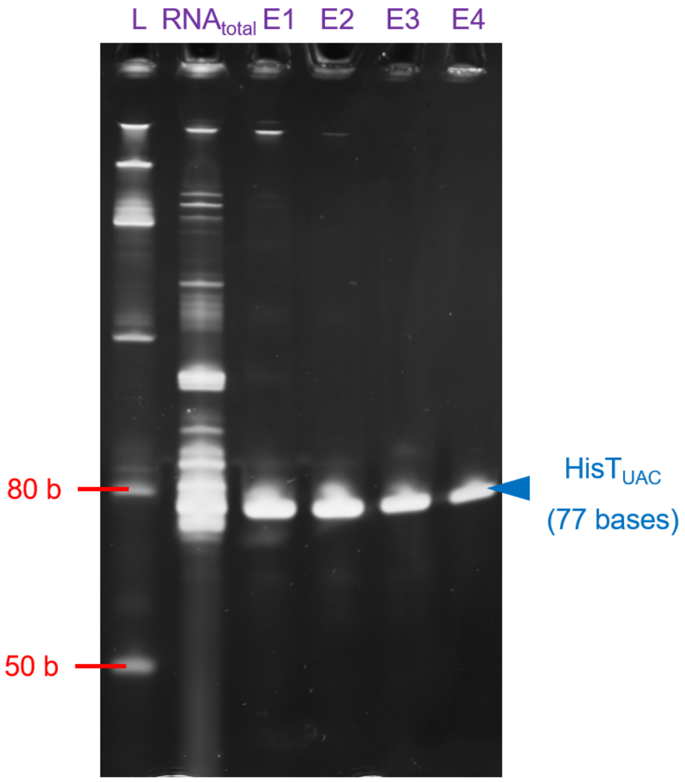

Supplementary fig. 22, Gel $HisT_{UAC}$ purification. Denaturing polyacrylamide gel with total RNA and elution fractions samples (E1-4) obtained from the purification of $HisT_{UAC}$. The *$hisT_{TAC}$* gene was expressed in in Sel_{aux}. $HisT_{UAC}$ (theoretical size 77 bases) was purified from the extracted RNA using complementary oligonucleotides attached to beads. L: ladder.

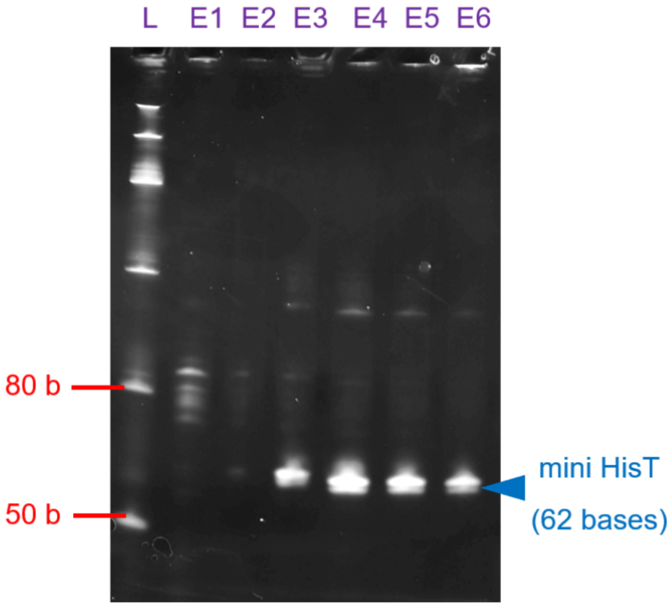

Supplementary fig. 23, Gel mini HisT purification Denaturing polyacrylamide gel with elution fractions samples (E1-6) obtained from the purification of mini HisT. The *mini $hisT_{TAC}$* gene was expressed in Sel^{0}_{hit}. Mini HisT (theoretical size 62 bases) was purified from the extracted RNA using complementary oligonucleotides attached to beads. L: ladder. Brightness of the entire picture increased.

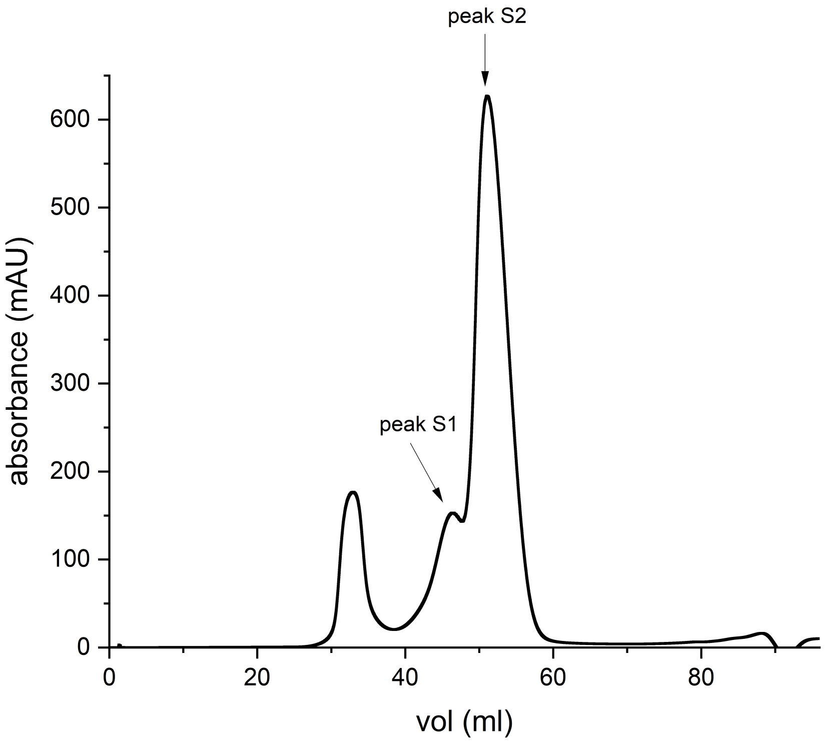

Supplementary fig. 24, Size-exclusion-chromatography profile of HisS with C-terminal twin-strep-tag. HisS containing a C-terminal twin-strep-tag was expressed in *E. coli* and purified by affinity chromatography followed by size exclusion chromatography. A Hiload 16/60 superdex 200 column was used. The two observed (non-aggregate) peaks were termed S1 and S2. An aliquot obtained from peak S2 was used for further assays.

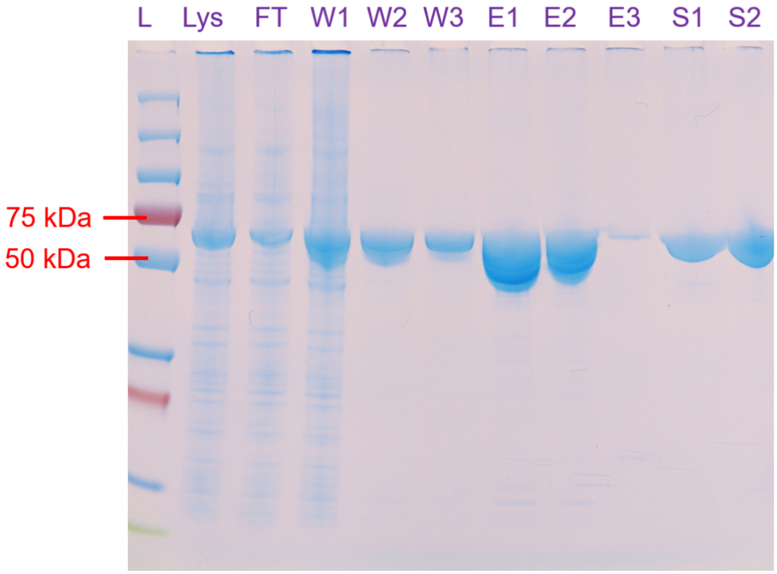

Supplementary fig. 25, SDS-PAGE gel with samples obtained during the purification of HisS with a C-terminal twin-strep-tag. HisS containing a C-terminal twin-strep-tag was expressed in *E. coli* and purified by affinity chromatography followed by size exclusion chromatography. $MW_{expected}$ (tagged HisS) = 56.4 kDa; L: ladder; Lys: lysate; FT: flow-through strep-tag purification column; W1-W3: wash fractions strep-tag purification column; E1-3: elution fractions strep-tag purification column; S1: sample from SEC peak S1; S2: sample from SEC peak S2. Brightness and contrast of the entire picture increased.

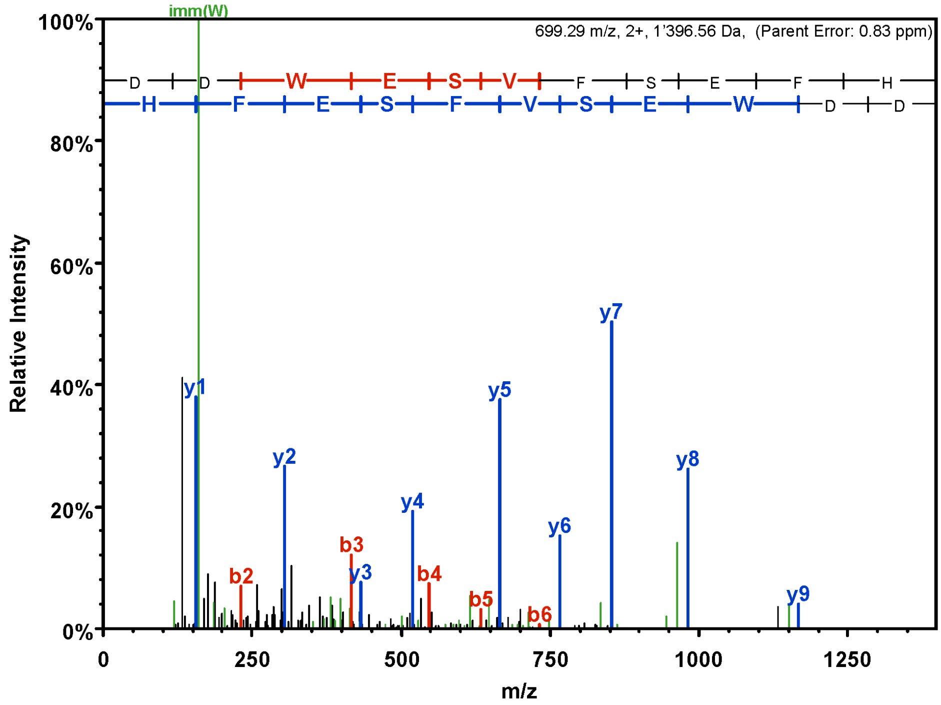

B	B Ions	B+2H	B-H2O	Y Ions	Y+2H	Y-H2O	Y
1	Missing,116.03		Missing,98.02	Missing,1'397.56	Missing,699.29	Missing,1'379.55	11
2	Found,231.06		Missing,213.05	Missing,1'282.54	Missing,641.77	Missing,1'264.53	10
3	Found,417.14		Found,399.13	Found,1'167.51	Missing,584.26	Found,1'149.50	9
4	Found,546.18		Found,528.17	Found,981.43	Missing,491.22	Found,963.42	8
5	Found,633.22		Found,615.20	Found,852.39	Missing,426.70	Found,834.38	7
6	Found,732.28	Missing,366.65	Found,714.27	Found,765.36	Missing,383.18	Found,747.35	6
7	Missing,879.35	Missing,440.18	Found,861.34	Found,666.29		Found,648.28	5
8	Missing,966.38	Missing,483.70	Missing,948.37	Found,519.22		Found,501.21	4
9	Missing,1'095.43	Missing,548.22	Missing,1'077.42	Found,432.19		Found,414.18	3
10	Missing,1'242.49	Missing,621.75	Missing,1'224.48	Found,303.15			2
11	Missing,1'397.56	Missing,699.29	Missing,1'379.55	Found,156.08			1

Supplementary fig. 26, Exemplary spectrum of peptide DDWESVFSEFH obtained by peptide mass fingerprinting and corresponding fragmentation table. A *folA* variant that contains a singular valine codon (for V136) was expressed in vitro using the PURE system in the presence of pre-aminoacylated mini HisT and HisS. The corresponding SDS-PAGE gel band of the DHFR variant (*folA* gene product) was analyzed by peptide mass fingerprinting (for details see materials and methods). The figure shows the measured spectrum assigned to the peptide DDWESVFSEFH containing the DHFR variant residue 136 (underlined, valine). The Peptide identification probability (scaffold internal algorithm) is 100 %.

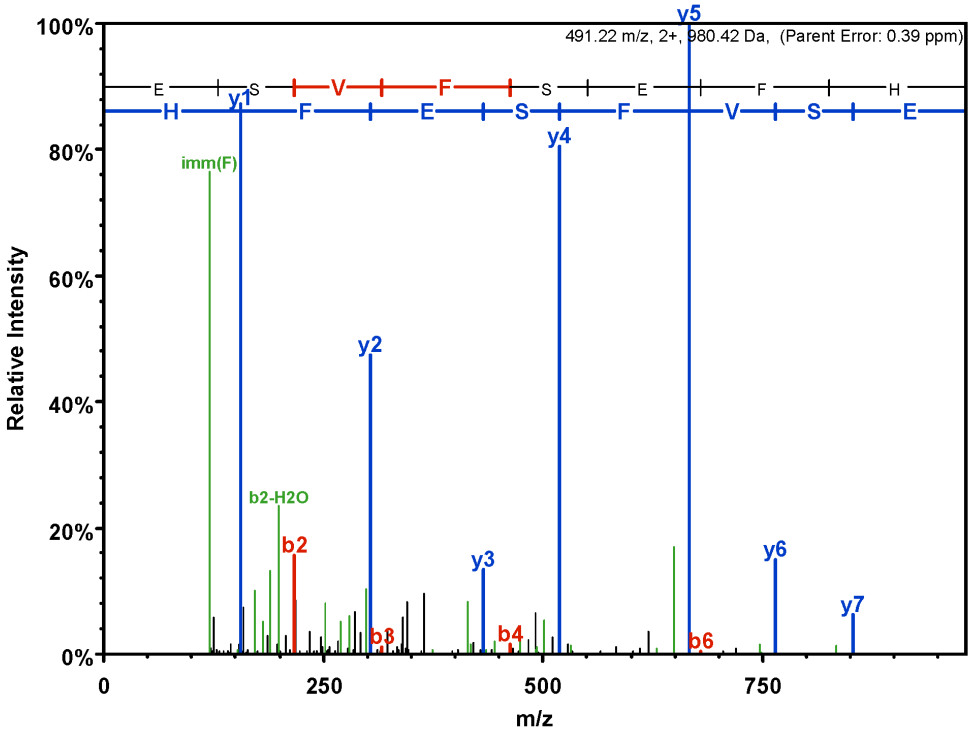

B	B Ions	B+2H	B-H2O	Y Ions	Y+2H	Y-H2O	Y
1	Missing,130.05		Missing,112.04	Missing,981.43	Missing,491.22	Missing,963.42	8
2	Found,217.08		Found,199.07	Found,852.39	Missing,426.70	Found,834.38	7
3	Found,316.15		Found,298.14	Found,765.36	Missing,383.18	Found,747.35	6
4	Found,463.22		Found,445.21	Found,666.29		Found,648.28	5
5	Missing,550.25		Found,532.24	Found,519.22		Found,501.21	4
6	Found,679.29	Missing,340.15	Missing,661.28	Found,432.19		Found,414.18	3
7	Missing,826.36	Missing,413.68	Missing,808.35	Found,303.15			2
8	Missing,981.43	Missing,491.22	Missing,963.42	Found,156.08			1

Supplementary fig. 27, Exemplary spectrum of peptide ESVFSEFH obtained by peptide mass fingerprinting and corresponding fragmentation table. A *folA* variant that contains a singular valine codon (for V136) was expressed in vitro using the PURE system in the presence of pre-aminoacylated mini HisT and HisS. The corresponding SDS-PAGE gel band of the DHFR variant (*folA* gene product) was analyzed by peptide mass fingerprinting (for details see materials and methods). The figure shows a measured spectrum assigned to the peptide ESVFSEFH containing the DHFR variant residue 136 (underlined, valine). The Peptide identification probability (scaffold internal algorithm) is 99 %.

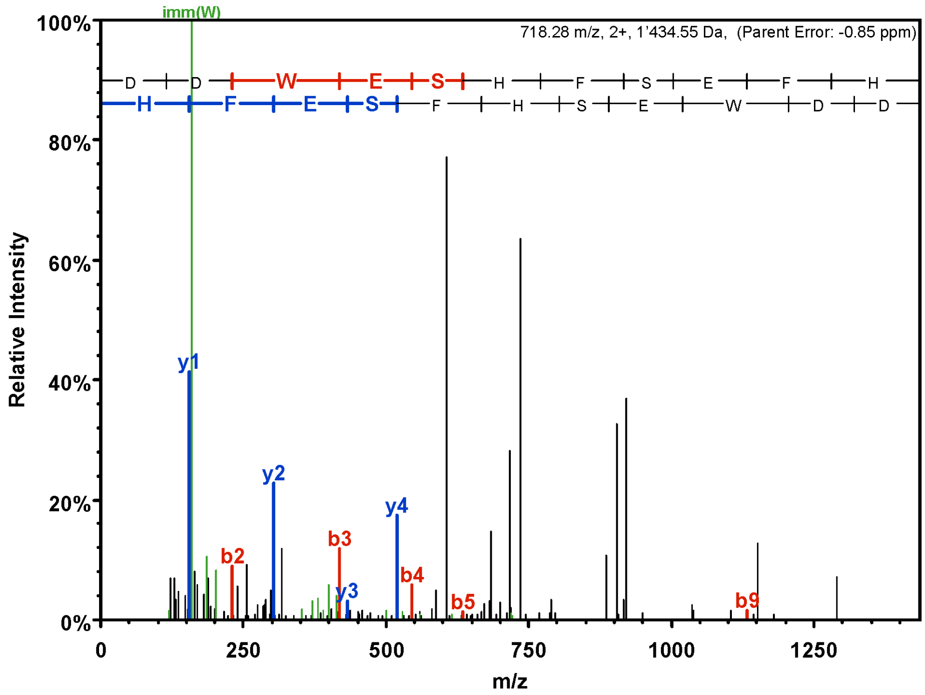

B	B Ions	B+2H	B-H2O	Y Ions	Y+2H	Y-H2O	Y
1	Missing,116.03		Missing,98.02	Missing,1'435.55	Missing,718.28	Missing,1'417.54	11
2	Found,231.06		Missing,213.05	Missing,1'320.53	Missing,660.77	Missing,1'302.52	10
3	Found,417.14		Found,399.13	Missing,1'205.50	Missing,603.25	Missing,1'187.49	9
4	Found,546.18		Found,528.17	Missing,1'019.42	Missing,510.21	Missing,1'001.41	8
5	Found,633.22		Found,615.20	Missing,890.38	Missing,445.69	Missing,872.37	7
6	Missing,770.27	Missing,385.64	Missing,752.26	Missing,803.35	Missing,402.18	Missing,785.34	6
7	Missing,917.34	Missing,459.17	Missing,899.33	Missing,666.29		Missing,648.28	5
8	Missing,1'004.37	Missing,502.69	Missing,986.36	Found,519.22		Found,501.21	4
9	Found,1'133.42	Missing,567.21	Missing,1'115.41	Found,432.19		Found,414.18	3
10	Missing,1'280.49	Missing,640.75	Missing,1'262.47	Found,303.15			2
11	Missing,1'435.55	Missing,718.28	Missing,1'417.54	Found,156.08			1

Supplementary fig. 28, Exemplary spectrum of peptide DDWESHFSEFH obtained by peptide mass fingerprinting and corresponding fragmentation table. A *folA* variant that contains a singular valine codon (for V136) was expressed in vitro using the PURE system in the presence of pre-aminoacylated mini HisT and HisS. The corresponding SDS-PAGE gel band of the DHFR variant (*folA* gene product) was analyzed by peptide mass fingerprinting (for details see materials and methods). The figure shows the measured spectrum assigned to the peptide DDWESHFSEFH containing the DHFR variant residue 136 (underlined, histidine). The Peptide identification probability (scaffold internal algorithm) is 100 %.

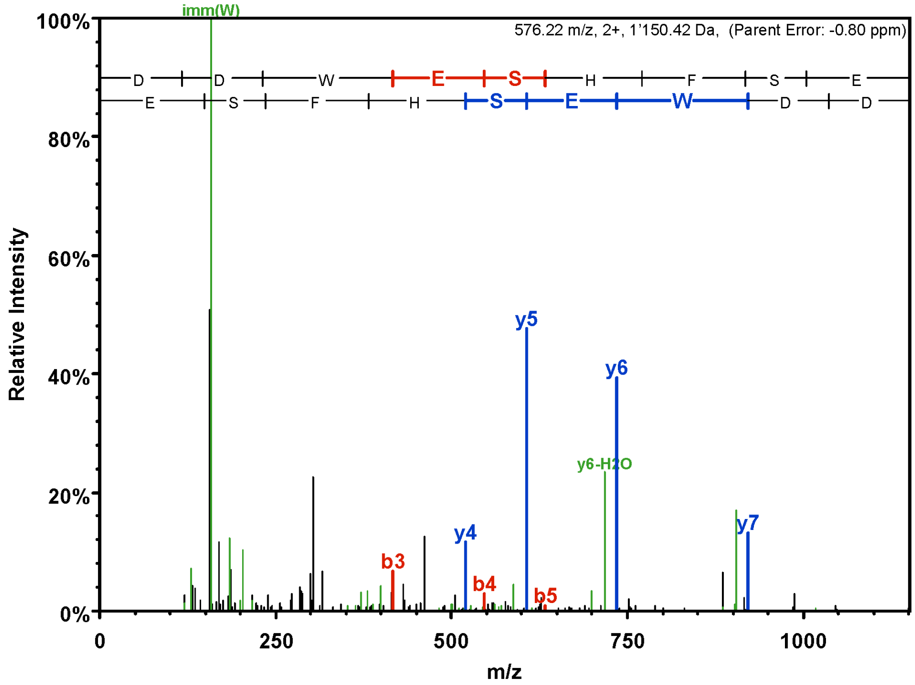

B	B Ions	B+2H	B-H2O	Y Ions	Y+2H	Y-H2O	Y
1	Missing,116.03		Missing,98.02	Missing,1'151.43	Missing,576.22	Missing,1'133.42	9
2	Missing,231.06		Missing,213.05	Missing,1'036.40	Missing,518.70	Found,1'018.39	8
3	Found,417.14		Found,399.13	Found,921.37	Missing,461.19	Found,903.36	7
4	Found,546.18		Found,528.17	Found,735.29	Missing,368.15	Found,717.28	6
5	Found,633.22		Found,615.20	Found,606.25		Found,588.24	5
6	Missing,770.27	Missing,385.64	Missing,752.26	Found,519.22		Found,501.21	4
7	Missing,917.34	Missing,459.17	Missing,899.33	Missing,382.16		Found,364.15	3
8	Missing,1'004.37	Missing,502.69	Missing,986.36	Missing,235.09		Found,217.08	2
9	Missing,1'151.43	Missing,576.22	Missing,1'133.42	Missing,148.06		Found,130.05	1

Supplementary fig. 29, Exemplary spectrum of peptide DDWESHFSE obtained by peptide mass fingerprinting and corresponding fragmentation table. A *folA* variant that contains a singular valine codon (for V136) was expressed in vitro using the PURE system in the presence of pre-aminoacylated mini HisT and HisS. The corresponding SDS-PAGE gel band of the DHFR variant (*folA* gene product) was analyzed by peptide mass fingerprinting (for details see materials and methods). The figure shows the measured spectrum assigned to the peptide DDWESHFSE containing the DHFR variant residue 136 (underlined, histidine). The Peptide identification probability (scaffold internal algorithm) is 99 %.

Supplementary tables

Supplementary table 1, *E. coli* strains used in this study.

Strain name	Genotype	Plasmids	Comment	Reference	WGS
BL21-DE3	F– ompT hsdSB (rB–, mB–) gal dcm (DE3)	-	Expression strain for protein production	Thermo Fisher	-
NEB turbo	F' $proA^+B^+$ $lacI^q$ ΔlacZM15 / fhuA2 Δ(lac-proAB) glnV galK16 galE15 R(zgb-210::Tn10)Tet^S endA1 thi-1 Δ(hsdS-mcrB)5	-	Cloning strain	New England Biolabs	-
DH5α	F– φ80lacZΔM15 Δ(lacZYA-argF)U169 recA1 endA1 hsdR17(rK–, mK+) phoA supE44 λ–thi-1 gyrA96 relA1	-	Cloning strain	Thermo Fisher	-
Sel_{aux}	K12 MG1655 Δ*thiE*::aad+FRT+ Δ*yjbQ*::aac+FRT+	-	Selection strain for suppression system	Gift from Valerie Pezo (unpublished)	Genotype confirmed by WGS
Sel_{aux}*	K12 MG1655 Δ*thiE* Δ*yjbQ*	-	Antibiotic resistances removed from Sel_{aux}	This study	Genotype confirmed by WGS
Sel_{hit}	K12 MG1655 Δ*thiE*::aad+FRT+ Δ*yjbQ*::aac+FRT+ *aspV*:T48G *pnp*:G573S	pThiN(H95V_{GTA}) pMiniHisT_{TAC}_HisS	Hit in adaptive evolution experiment	This study	Genotype and plasmid sequences confirmed by WGS
Sel^0_{hit}	K12 MG1655 Δ*thiE*::aad+FRT+ ΔyjbQ::aac+FRT+ *aspV*:T48G *pnp*:G573S	-	Plasmids cured from Sel_{hit}	This study	-

Sel^0_{hit}*	K12 MG1655 Δ*thiE* Δ*yjbQ* *aspV*:T48G *pnp*:G573S	-	Antibiotic resistances removed from Sel^0_{hit}	This study	Genotype confirmed by WGS
$Sel_{eng\_pnp-mut}$	K12 MG1655 Δ*thiE* Δ*yjbQ* *pnp*:G573S	pThiN(H95V_{GTA}) pMiniHisT_{TAC}_HisS	*pnp*:G573S introduced in targeted manner into Sel_{aux}*	This study	Genotype and plasmid sequences confirmed by WGS
$Sel_{aux\_pnp-mut}$	K12 MG1655 Δ*thiE* Δ*yjbQ* *pnp*:G573S	-	Plasmids cured from $Sel_{eng\_pnp-mut}$	This study	-

Supplementary table 2, Plasmids used in this study.

#	Plasmid name	Comment	Application	Reference
1	pSEVA191	Empty plasmid backbone (Carb resistance, pBR322 ori)	Negative control	(1)
2	pThiN	pSEVA19 backbone, wt *thiN* under control of BBaJ23100 promoter retrieved from Registry of standard biological parts (2) and a synthetic RBS designed by RBS calculator v2.0 with TIR of 23000 (3)	Expression of wt *thiN*	This study
3	pHisT_{TAC}	pSEVA36 backbone (Cm resistance, p15A ori), *hisT_{TAC}* under control of *E. coli* proK promoter and ending with proK terminator (4)	Expression of *hisT_{TAC}*	This study
4	pHisS	pSEVA36 backbone, *hisS* under control of glnS' promoter and RBS (4)	Expression of *hisS*	This study
5	pHisT_{TAC}_HisS	pSEVA36 backbone, *hisT_{TAC}* and *hisS*	Expression of *hisT_{TAC}* and *hisS*	This study
6	pThiN(H95V_{GTA})	pSEVA19 backbone, *thiN*:H95V under control of BBaJ23100 and a synthetic RBS with TIR of 23000	Expression of *thiN*:H95V variant	This study
7	pKD46-Kan	pDK46 derived plasmid with Kan resistance, pSC101 ori, lambda red genes under control of P_{BAD} system (5)	Recombineering	This study
8	pMiniHisT_{TAC}_HisS	pSEVA36 backbone, *mini hisT_{TAC}* under control of *E. coli* proK promoter and ending with proK terminator and *hisS*	Expression of *mini hisT_{TAC}* and *hisS*	This study

9	pMiniHisT$_{TAC}$	pSEVA36 backbone, *mini hisT$_{TAC}$*	Expression of mini *hisT$_{TAC}$*	This study
10	pMiniHisT$_{CCT}$_HisS	pSEVA36 backbone, *mini hisT$_{CCT}$* and *hisS*	Expression of *mini hisT$_{CCT}$* and *hisS*	This study
11	pHisS_tst_pur	pET30b backbone (Kan resistance, pBR322 ori), *hisS* with C-terminal twin-strep-tag under control of T7 expression system	Expression of *hisS* for protein purification	This study
12	pHisT$_{TAC}$_pur	pSEVA19 backbone, *hisT$_{TAC}$* under control of *E. coli* proK promoter and ending with proK terminator	Expression of *hisT$_{TAC}$* for purification	This study
13	pMiniHisT$_{TAC}$_pur	pSEVA19 backbone, *mini hisT$_{TAC}$* under control of *E. coli* proK promoter and ending with proK terminator	Expression of *mini hisT$_{TAC}$* for purification	This study
14	pDHFRvar	Modified commercial PURExpress plasmid, Carb resistance, ColE1 ori, *E. coli folA* variant under control of T7 promoter and RBS	Expression of *folA* variant	This study
15	pCP20	Carb resistance, pSC101 ori, flipase *flp* under control of P_R lambda promoter and RBS	Removal of antibiotic resistance cassettes	(6)
16	pHisT(I)_HisS	pSEVA36 backbone, non-canonical tRNA design I and *hisS*	Expression of non-canonical tRNA and *hisS*	This study
17	pHisT(II)_HisS	pSEVA36 backbone, non-canonical tRNA design II and *hisS*	Expression of non-canonical tRNA and *hisS*	This study
18	pHisT(III)_HisS	pSEVA36 backbone, non-canonical tRNA design III and *hisS*	Expression of non-canonical tRNA and *hisS*	This study
19	pHisT(IV)_HisS	pSEVA36 backbone, non-canonical tRNA design IV and *hisS*	Expression of non-canonical tRNA and *hisS*	This study
20	pHisT(V)_HisS	pSEVA36 backbone, non-canonical tRNA design V and *hisS*	Expression of non-canonical tRNA and *hisS*	This study
21	pHisT(VI)_HisS	pSEVA36 backbone, non-canonical tRNA design VI and *hisS*	Expression of non-canonical tRNA and *hisS*	This study
22	pHisT(VII)_HisS	pSEVA36 backbone, non-canonical tRNA design VII and *hisS*	Expression of non-canonical tRNA and *hisS*	This study
23	pHisT(VIII)_HisS	pSEVA36 backbone, non-canonical tRNA design VIII and *hisS*	Expression of non-canonical tRNA and *hisS*	This study

24	pHisT(IX)_HisS	pSEVA36 backbone, non-canonical tRNA design IX and *hisS*	Expression of non-canonical tRNA and *hisS*	This study
25	pHisT(XI)_HisS	pSEVA36 backbone, non-canonical tRNA design XI and *hisS*	Expression of non-canonical tRNA and *hisS*	This study
26	pHisT(XII)_HisS	pSEVA36 backbone, non-canonical tRNA design XII and *hisS*	Expression of non-canonical tRNA and *hisS*	This study
27	pThiN(H95I_{ATA})	pSEVA19 backbone, *thiN*:H95I_{ATA}	Expression of *thiN* variant	This study
28	pThiN(H95M_{ATG})	pSEVA19 backbone, *thiN*:H95M_{ATG}	Expression of *thiN* variant	This study
29	pThiN(H95S_{TCA})	pSEVA19 backbone, *thiN*:H95S_{TCA}	Expression of *thiN* variant	This study
30	pThiN(H95T_{ACA})	pSEVA19 backbone, *thiN*:H95T_{ACA}	Expression of *thiN* variant	This study
31	pThiN(H95A_{GCG})	pSEVA19 backbone, *thiN*:H95A_{GCG}	Expression of *thiN* variant	This study
32	pThiN(H95R_{AGA})	pSEVA19 backbone, *thiN*:H95R_{AGA}	Expression of *thiN* variant	This study
33	pThiN(H95G_{GGA})	pSEVA19 backbone, *thiN*:H95G_{GGA}	Expression of *thiN* variant	This study
34	pThiN(H95L_{TTA})	pSEVA19 backbone, *thiN*:H95L_{TTA}	Expression of *thiN* variant	This study
35	pThiN(H95D_{GAT})	pSEVA19 backbone, *thiN*:H95D_{GAT}	Expression of *thiN* variant	This study
36	pThiN(H95W_{TGG})	pSEVA19 backbone, *thiN*:H95W_{TGG}	Expression of *thiN* variant	This study
37	pThiN(H95F_{TTC})	pSEVA19 backbone, *thiN*:H95F_{TTC}	Expression of *thiN* variant	This study
38	pThiN(H95P_{CTT})	pSEVA19 backbone, *thiN*:H95P_{CTT}	Expression of *thiN* variant	This study

39	pThiN(H95Y_{TAT})	pSEVA19 backbone, *thiN*:H95Y_{TAT}	Expression of *thiN* variant	This study
40	pThiN(H95Q_{CAG})	pSEVA19 backbone, *thiN*:H95Q_{CAG}	Expression of *thiN* variant	This study
41	pThiN(H95N_{AAT})	pSEVA19 backbone, *thiN*:H95N_{AAT}	Expression of *thiN* variant	This study
42	pThiN(H95K_{AAG})	pSEVA19 backbone, *thiN*:H95K_{AAG}	Expression of *thiN* variant	This study
43	pThiN(H95E_{GAG})	pSEVA19 backbone, *thiN*:H95E_{GAG}	Expression of *thiN* variant	This study
44	pThiN(H95C_{TCG})	pSEVA19 backbone, *thiN*:H95C_{TCG}	Expression of *thiN* variant	This study
45	pThiN(H95L_{TTG})	pSEVA19 backbone, *thiN*:H95L_{TTG}	Expression of *thiN* variant	This study
46	pThiN(H95$*_{TGA}$)	pSEVA19 backbone, *thiN*:H95$*_{TGA}$	Expression of *thiN* variant	This study
47	pThiN(H95R_{AGG})	pSEVA19 backbone, *thiN*:H95R_{AGG}	Expression of *thiN* variant	This study
48	pThiN(H95G_{GGG})	pSEVA19 backbone, *thiN*:H95G_{GGG}	Expression of *thiN* variant	This study
49	pThiN(H95A_{GCA})	pSEVA19 backbone, *thiN*:H95A_{GCA}	Expression of *thiN* variant	This study
50	pThiN(H95V_{GTG})	pSEVA19 backbone, *thiN*:H95V_{GTG}	Expression of *thiN* variant	This study
51	pThiN(H95S_{TCA})	pSEVA19 backbone, *thiN*:H95S_{TCA}	Expression of *thiN* variant	This study
52	pThiN(H95T_{ACG})	pSEVA19 backbone, *thiN*:H95T_{ACG}	Expression of *thiN* variant	This study

Supplementary table 3, Oligonucleotides used in this study.

Oligo name	Sequence (5' to 3')	Application	Modifications	Vendor
prMPE95	GGTGATCGTCAATTCAGAAT	Amplification *yjbq* locus by colony PCR, forward primer	-	Microsynth AG
prMPE96	ACATCTTCCACTTTGATCTG	Amplification *yjbq* locus by colony PCR, reverse primer	-	Microsynth AG
prMPE97	CAGACCGATAATCAGCACCT	Amplification *thiE* locus by colony PCR, forward primer	-	Microsynth AG
prMPE98	TGCTCGATGAAAATCAGCCA	Amplification *thiE* locus by colony PCR, reverse primer	-	Microsynth AG
prMPE311	TGACGGCAGCAATTCA	Amplification *pnp* locus by colony PCR, reverse primer	-	Microsynth AG
prMPE312	CACTCCCGAAGACCAC	Amplification *pnp* locus by colony PCR, forward primer	-	Microsynth AG
mMPE7	GTGGTGCCAGTTTCTTCGGTCAGAGCACGGATTACAGAAC TGCCTTTACCGATAACATCTTTGATCTTGTCCGGGTTGAT	Introduction of genomic *pnp* point mutation to generate *pnp*:G573S by recombineering	5 nucleotides at 5'-end with phosphorothioate linkage	Merck
probeMPE2	TACCGCCTCCCCGGCTCGAACGGGGGACCT	Purification of $HisT_{UAC}$ or mini HisT	Biotinylated at 3'-end	Integrated DNA Technologies
qpcrMPE1	GCCAAGACGGTTGAAGATGC	Amplification *ihfB* reference in qPCR, forward primer	-	Microsynth AG
qpcrMPE2	GAGAAACTGCCGAAACCGC	Amplification *ihfB* reference in qPCR, reverse primer	-	Microsynth AG
qpcrMPE3	GCCGCCTTAGCGCTAGAT	Amplification *mini hisT* in qPCR, forward primer	-	Microsynth AG
qpcrMPE4	TACCGCCTCCCCGG	Amplification *mini hisT* in qPCR, reverse primer	-	Microsynth AG

Supplementary table 4, Testing *thiN*:H95X variants in growth experiment reveals only H confers immediate growth. Sel$_{aux}$ was transformed with plasmids harboring *thiN* variants mutated in the codon for residue 95 in ThiN. Variants with codons for all 20 proteinogenic amino acids were tested individually. For each transformed strain, a single colony obtained after the transformation was selected and transferred to a M9(glu) plate. After a period of 5 days, we evaluated whether the respective strain showed growth or not.

ThiN H95X variant (codon)	Growth observed on M9(glu) plates
H (CAC)	+
I (ATA)	-
M (ATG)	-
V (GTA)	-
S (TCA)	-
T (ACA)	-
A (GCG)	-
R (AGA)	-
G (GGA)	-
L (TTA)	-
D (GAT)	-
W (TGG)	-
F (TTC)	-
P (CCT)	-
Y (TAT)	-
Q (CAG)	-
N (AAT)	-
K (AAG)	-
E (GAG)	-
C (TGC)	-

Supplementary table 5, Some non-histidine *thiN*:H95X variants facilitate selection escape in adaptive evolution experiment. Sel_{aux} was transformed with a plasmid harboring one of the *thiN* variants, in which the codon encoding for H95 was mutated to a codon that differed in all three bases from any histidine codon. An adaptive evolution experiment on M9(glu) plates was performed to investigate whether escapees occur for the respective mutated codon. For each of the 18 strains, 150 µl cells with an OD_{600} of 1 were plated on M9(glu) agar. Three plates per variant were prepared and incubated for 14 d. The emergence of colonies was evaluated at the end of the incubation period.

ThiN H95X variant (codon)	Growth observed on M9(glu) plates
L (TTG)	-
L (TTA)	-
* (TGA)	-
I (ATA)	-
W (TGG)	-
M (ATG)	-
R (AGG)	+
R (AGA)	+
G (GGA)	-
G (GGG)	-
A (GCA)	-
A (GCG)	-
V (GTG)	-
V (GTA)	-
S (TCA)	+
S (TCG)	+
T (ACA)	-
T (ACG)	-

Supplementary table 6, Thiamine-utilizing enzymes and downstream products. *E. coli* MG1655 genes encoding for enzymes with thiamine as cofactor and their metabolic function. Listed metabolites are produced downstream of the reaction catalyzed by the enzyme.

**Gene encoding for enzyme with thiamine as cofactor**	**Involved in metabolic function**	**Downstream metabolite and used concentration**
sucA	citric acid cycle	1 mM succinate
aceE	pyruvate decarboxylation	10 mM acetate
tktA/tktB	pentose phosphate pathway	1 µM pyridoxal, 1 mM shikimate
poxB	pyruvate decarboxylation	10 mM acetate
dxs	isopentenyl diphosphate biosynthesis	1 µM pyridoxal, 0.5 mM isoprenol
ilvB/ilvN and *ilvL/ilvH*	Ile, Leu, Val and pan biosynthesis	0.5 mM Val, 0.5 mM Leu, 0.5 mM Ile
menD	menaquinone biosynthesis	-
gcl	glyoxylate degradation	-
panB	Phosphopantothenate biosynthesis	2 µM pantothenate

Supplementary table 7, No metabolic escape is observed for incomplete suppression system in long-term adaptive evolution experiments. Sel_{aux} was transformed with incomplete suppression systems either lacking *hisS* or $hisT_{TAC}$. The resulting strains were incubated on $M9(glu)^{sup}$ agar plates or in liquid culture for 26 d and 30 d, respectively. The experiment was performed in biological triplicates (three plates and cultures each). *) No escape detected in any of the cases.

**Condition**	**Plasmids in strain Sel_{aux}**	**Escape frequency ***
$M9(glu)^{sup}$ agar plates	pHisS and pThiN($H95V_{GTA}$)	$< 7 * 10^{-10}$
	$pHisT_{TAC}$ and pThiN($H95V_{GTA}$)	$< 7 * 10^{-10}$
$M9(glu)^{sup}$ liquid medium	pHisS and pThiN($H95V_{GTA}$)	$< 2 * 10^{-12}$
	$pHisT_{TAC}$ and pThiN($H95V_{GTA}$)	$< 2 * 10^{-12}$

Supplementary table 8, Only for the non-canonical tRNA design X, a hit is obtained during adaptive evolution experiment. Non-canonical HisT-derived tRNA designs I-XII are listed with their containing nucleotides. These were used to replace $HisT_{UAC}$ in the suppression system. Sel_{aux} was transformed with pThiN($H95V_{GTA}$) and one of the plasmids indicated below. The resulting strains were subjected to long-term adaptive evolution experiments on M9(glu)^{sup} agar plates for 21 d and the emergence of colonies was monitored.

Non-canonical tRNA design [plasmid name]	Nucleotides present in construct (numbering according to full length HisT)	Colony observed	Time until colony
I [pHisT(I)_HisS]	[1-7; 50-77]	-	-
II [pHisT(II)_HisS]	[1-28; 44-77]	-	-
III [pHisT(III)_HisS]	[1-32; 40-77]	-	-
IV [pHisT(IV)_HisS]	[1-7; 32-40; 67-77]	-	-
V [pHisT(V)_HisS]	[1-7; 28-44; 67-77]	-	-
VI [pHisT(VI)_HisS]	[1-10; 26-50; 66-77]	-	-
VII [pHisT(VII)_HisS]	[1-11; 25-51; 65-77]	-	-
VIII [pHisT(VIII)_HisS]	[1-13; 23-54; 62-77]	-	-
IX [pHisT(IX)_HisS]	[1-7; 28-77]	-	-
X [pMiniHisT$_{TAC]}$	[1-10; 26-77]	+	10 d
XI [pHisT(XI)_HisS]	[1-50; 66-77]	-	-
XII [pHisT(XII)_HisS]	[1-54; 62-77]	-	-

Supplementary table 9, Measurement of histidine and valine incorporation at target position 136 in DHFR variants by peptide mass fingerprinting. Gel bands of the DHFR variants from PURE reactions that contained either HisS and mini HisT (3) or only HisS (4) were used. Following proteolytic digest, resulting peptides were analyzed by LC-MS/MS. The detected peptides were mapped against the sequence of DHFR variants. The count of all identified peptides that contained the suppression target residue (136 in DHFR variants) for condition (3) and (4) are shown in the table. For each experimental condition two independent gel bands (two replicates) were analyzed. The individual results for each gel band and the pooled results are presented.

	**Number of times peptide detected in sample (3): mini HisT in PURE**			**Number of times peptide detected in sample (4): no mini HisT in PURE**		
**Peptide identified**	**Replicate 1**	**Replicate 2**	**Replicate 1+2**	**Replicate 1**	**Replicate 2**	**Replicate 1+2**
DDWES**V**FSEFH	5	0	5	4	0	4
ES**V**FSEFH	7	8	15	8	3	11
DDWES**H**FSEFH	3	0	3	0	0	0
DDWES**H**FSE	7	4	11	0	0	0

Supplementary sequences of selected plasmids

Complete plasmid sequence of pHisT$_{TAC}$_HisS (nucleotides 1-3794)

hisT$_{TAC}$ under control of proK promoter and followed by proK terminator; *hisS* under control of glnS′ promoter and RBS

1_GCGGCCGCTTATCCCAGGATGCGCCGGACTTCCGCGACCAGGTCGGCGCGGGCCACCTCGGTCTGGGCGGGCTGGGCCTTCCATTCCTCGTGGCTGGCCTCGGCCGCGATCTTCGCGC
CCAGGACCAGATCCTTGACCTGCACGACGCCGCGCGCCGCCTCGTCCCCGCCCTGGATGATGGCGACGGGGGCATTGCGCTTGTCGGCATATTTCAACTGGTTGCCGAAGTTCTTGGGATT
GCCCAGATAGACCTCGGCCCGGATGCCGGCGGCGCGCAGTTCGGCGGCCATGGCCTGGTAATCGGCCATGCGTTCGCGATCCATGACGGTGACGACGACGGGGCCCTGTTCGGTCCCGC
CCATCAACCCCTTGGCGCGCAGGGCGGCAAGAAGCCGGTCGACGCCGATGGAAACCCCGGTCGCGGGCACCTTCTGGCCGGTGAAGCGTTCCACCAGCCCGTCATAGCGCCCGCCGCCG
GCGACCGAGCCGAACTGGCGCTTGCGGCCTTTGTCGTCGAGGATCTCGAAGGTCAGTTCGGCCTCGAAGACCGGGCCGGTGTAATAGCCCAGGCCCCGCACGATGGAAGGGTCGACGAT
GGCGCGCTCCTCGCCCACGCCCATGGCGGCGAGCATCCGGGCGATCTGCGCCAGTTCCTCGACGCCCTCGGCCCCGGTGGCCGAGGCGCCCACGGCGGCGCGCAGGTTTGCCAGCGTCC
CGGCATTGTCCGCGCCTTTCGAGGTCAGGAAGGCCAGCACCGGCTCGGCCTGTTCGGGGCTCAGGCCCACGCCCTCGATGAAGGCGCCGGATTCGTCCTTGCGGCCGGTGGTCAGCAATT
GCCGCACGCCTTGCTCGCCGACTTTGTCGAACTTGTCGATGGTGCGCAGCACGTCGTCGGCGGGCTTGCCCTCGGCCACGCCCATGGATTCCAGGATGCCGTTCAGCACCTTGCGGTTGTT
GATGCGGATCAGGTAGTCGCCGCGCCGGATGCCGGCATGCTCCAGCGCCGCAGCCAGCATGGCGCAGATCTCGGCATCCGCGGCGACCGAAGCCGAGCCCACGGTATCGGCATCGCATT
GGTAGAACTGGCGGAAGCGGCCGGGGCCGGGCTTTTCGTTGCGCCAGACCGGGCCCATGGCATAGCGGCGATAGGGCGACGGCAGGTCACCCCGGAACTGCGCGGCGACGCGCGCAA
GCGGCGCGGTCAGGTCATAGCGCAACGCCAGCCAGTCGCCGGCCCCGCCGCCGGGAACCTCTGCCTCTTGCCAGGCAAAGACGCCGGCATTGGGGCGGTCCACGTCGGGCAGGAACTTT
CCCAGCGCCTCGACCGTTTCGACGGCACTGGTTTCAAGCGGCTCGAAGCCGTGGCGATGATAGATCTGGGCGATGCGGTCGAGCATCTGCTTGCGCTCGGTCACATCCGCGCCGAAATAG
TCTCGGAACCCCTTGGGCGTCTCGGCCTTGGGACGGGGCTGTTTCTTCTGATCTTTCGCCATTAATTAATTCCTCAAAGCGTAAACAACGTATAACGGCGTATGATATTAAAGCGGGACAGG
CTGACAACTGTTAGTTTTTTAAAATTGATAATCTAACAAGGATTATGGGGATGATTGATGCCTGCAGGAGCGGATAACAATTTCGCGATCGCGGCTAACTAAGCGGCCTGCTGACTTTCTCG
CCGATCAAAAGGCATTTTGCTATTAAGGGATTGACGAGGGCGTATCTGCGCAGTAAGATGCGCCCCGCATTGCCGCCTTAGCTCAGTTGGTTAGAGCGCTAGATT**TAC**GATCTAGAGGTCC
CCCGTTCGAGCCGGGGAGGCGGTACCAAATTCGAAAAGCCTGCTCAACGAGCAGGCTTTTTTGCATGTTTAAACGGGTCCCCAATAATTACGATGGCGAAAATGAGACGTTGATCGGCACG
TAAGAGGTTCCAACTTTCACCATAATGAAATAAGATCACTACCGGGCGTATTTTTTGAGTTATCGAGATTTTCAGGAGCTAAGGAAGCTAAAATGGAGAAAAAAATCACTGGATATACCACC
GTTGATATATCCCAATGGCATCGTAAAGAACATTTTGAGGCATTTCAGTCAGTTGCTCAATGTACCTATAACCAGACCGTTCAGCTGGATATTACGGCCTTTTTAAAGACCGTAAAGAAAAAT
AAGCACAAGTTTTATCCGGCCTTTATTCACATTCTTGCCCGCCTGATGAATGCTCATCCGGAATTTCGTATGGCAATGAAAGACGGTGAGCTGGTGATATGGGATAGTGTTCACCCTTGTTAC
ACCGTTTTCCATGAGCAAACTGAAACGTTTTCATCGCTCTGGAGTGAATACCACGACGATTTCCGGCAGTTTCTACACATATATTCGCAAGATGTGGCGTGTTACGGTGAAAACCTGGCCTAT
TTCCCTAAAGGGTTTATTGAGAATATGTTTTTCGTCTCAGCCAATCCCTGGGTGAGTTTCACCAGTTTTGATTTAAACGTGGCCAATATGGACAACTTCTTCGCCCCCGTTTTCACCATGGGCA
AATATTATACGCAAGGCGACAAGGTGCTGATGCCGCTGGCGATTCAGGTTCATCATGCCGTTTGTGATGGCTTCCATGTCGGCAGAATGCTTAATGAATTACAACAGTACTGCGATGAGTGG
CAGGGCGGGGCGTAATTTGACTTTCGCGCGTTGTCCTTTTCCGCTGCATAACCCTGCTTCGGGGTCATTATAGCGATTTTTTCGGTATATCCATCCTTTTTCGCACGATATACAGGATTTTGCC
AAAGGGTTCGTGTAGACTTTCCTTGGTGTATCCAACGGCGTCAGCCGGGCAGGATAGGTGAAGTAGGCCCACCCGCGAGCGGGTGTTCCTTCTTCACTGTCCCTTATTCGCACCTGGCGGT
GCTCAACGGGAATCCTGCTCTGCGAGGCTGGCCGTAGGCCGGCCCTAGAAATATTTTATCTGATTAATAAGATGATCTTCTTGAGATCGTTTTGGTCTGCGCGTAATCTCTTGCTCTGAAAAC
GAAAAAACCGCCTTGCAGGGCGGTTTTTCGAAGGTTCTCTGAGCTACCAACTCTTTGAACCGAGGTAACTGGCTTGGAGGAGCGCAGTCACCAAAACTTGTCCTTTCAGTTTAGCCTTAACC
GGCGCATGACTTCAAGACTAACTCCTCTAAATCAATTACCAGTGGCTGCTGCCAGTGGTGCTTTTGCATGTCTTTCCGGGTTGGACTCAAGACGATAGTTACCGGATAAGGCGCAGCGGTCG
GACTGAACGGGGGGTTCGTGCATACAGTCCAGCTTGGAGCGAACTGCCTACCCGGAACTGAGTGTCAGGCGTGGAATGAGACAAACGCGGCCATAACAGCGGAATGACACCGGTAAACC
GAAAGGCAGGAACAGGAGAGCGCACGAGGGAGCCGCCAGGGGGAAACGCCTGGTATCTTTATAGTCCTGTCGGGTTTCGCCACCACTGATTTGAGCGTCAGATTTCGTGATGCTTGTCAG
GGGGGCGGAGCCTATGGAAAAACGGCTTTGCCGCGGCCCTCTCACTTCCCTGTTAAGTATCTTCCTGGCATCTTCCAGGAAATCTCCGCCCCGTTCGTAAGCCATTTCCGCTCGCCGCAGTC
GAACGACCGAGCGTAGCGAGTCAGTGAGCGAGGAAGCGGAATATATCCGGCGCGCCCAGCTGTCTAGGGCGGCGGATTTGTCCTACTCAGGAGAGCGTTCACCGACAAACAACAGATAA
AACGAAAGGCCCAGTCTTTCGACTGAGCCTTTCGTTTTATTTGATGCC_**3794**

Complete plasmid sequence of pThiN(H95V$_{GTA}$) (nucleotides 1-3635)

thiN:H95V(CAC→**GTA**) under control of BBaJ23100 promoter and synthetic RBS

1_CCTGCAGGTTGACGGCTAGCTCAGTCCTAGGTACAGTGCTAGCTCCACTAAAATTATAATTATAAGGGAGGTTAATTAATGGAGCTTCTGAAGAAGATCTTAGAACGCTCGTCGGGGGTC
GTGGAGACTGAAAAATTACTTCGCGACTGGTATCGTTACGATACTCTGAACACTTTAGATGAGATTCTGCCTGAGTTTTTAGAAATCGGGCATTTAACAGTTCCTGAAGTTGGTCAGAATGTG
TCCTACGCGCTTCCCTGGGCCAAGAACGAATTCGAGGTTGGCAAGTTTCCTGGTCGCATTCGTCTTAAAGAAGGGAAAGCCGTGGCGGTGAGTTGCGCATCATTCAAAGATCGCTCC**GTA**A
CCGCCCGTATGGCAGTCACTATGATGCGCTACCATCCCCACATGCGCTGTGTGGTTAATGTGCGCTACGAACGTGAGTATGTGGAACGTGCCAAGAAACGCGGGTTAAAAGTTTTCCATTAC
GATCGTTCCAAGGAGCCGAAAGAAGTCCAGGAAAAGGAAGGCCAAAGTATGGTGTGGATGATCGAGCAAGCTATCGCAGAGTTAAAATCTCCTCCTGACGTGATTTACGACGAAGGCTGG
TGGGGTAAGGAAGCGATGATTCGTGTGTTTGGGCGTAACCCAAAGGAAGTCCTGGAGAAAATTAAACTTATGGTTCGTGAGTAATAAACTAGTCTTGGACTCCTGTTGATAGATCCAGTAAT
GACCTCAGAACTCCATCTGGATTTGTTCAGAACGCTCGGTTGCCGCCGGGCGTTTTTTATTGGTGAGAATCCAGGGGTCCCCAATAATTACGATTTAAATTAGTAGCCCGCCTAATGAGCGG
GCTTTTTTTTAATTCCCCTATTTGTTTATTTTTCTAAATACATTCAAATATGTATCCGCTCATGAGACAATAACCCTGATAAATGCTTCAATAATATTGAAAAAGGAAGAGTATGAGCATTCAGC
ATTTTCGTGTGGCGCTGATTCCGTTTTTTGCGGCGTTTTGCCTGCCGGTGTTTGCGCATCCGGAAACCCTGGTGAAAGTGAAAGATGCGGAAGATCAACTGGGTGCGCGCGTGGGCTATATT
GAACTGGATCTGAACAGCGGCAAAATTCTGGAATCTTTTCGTCCGGAAGAACGTTTTCCGATGATGAGCACCTTTAAAGTGCTGCTGTGCGGTGCGGTTCTGAGCCGTGTGGATGCGGGCC
AGGAACAACTGGGCCGTCGTATTCATTATAGCCAGAACGATCTGGTGGAATATAGCCCGGTGACCGAAAAACATCTGACCGATGGCATGACCGTGCGTGAACTGTGCAGCGCGGCGATTA
CCATGAGCGATAACACCGCGGCGAACCTGCTGCTGACGACCATTGGCGGTCCGAAAGAACTGACCGCGTTTCTGCATAACATGGGCGATCATGTGACCCGTCTGGATCGTTGGGAACCGG
AACTGAACGAAGCGATTCCGAACGATGAACGTGATACCACCATGCCGGCAGCAATGGCGACCACCCTGCGTAAACTGCTGACGGGTGAGCTGCTGACCCTGGCAAGCCGCCAGCAACTG
ATTGATTGGATGGAAGCGGATAAAGTGGCGGGTCCGCTGCTGCGTAGCGCGCTGCCGGCTGGCTGGTTTATTGCGGATAAAAGCGGTGCGGGCGAACGTGGCAGCCGTGGCATTATTGCG
GCGCTGGGCCCGGATGGTAAACCGAGCCGTATTGTGGTGATTTATACCACCGGCAGCCAGGCGACGATGGATGAACGTAACCGTCAGATTGCGGAAATTGGCGCGAGCCTGATTAAACAT
TGGTAAACCGATACAATTAAAGGCTCCTTTTGGAGCCTTTTTTTTTGGACGACCCTTGTCCTTTTCCGCTGCATAACCCTGCTTCGGGGTCATTATAGCGATTTTTTCGGTATATCCATCCTTTTT
CGCACGATATACAGGATTTTGCCAAAGGGTTCGTGTAGACTTTCCTTGGTGTATCCAACGGCGTCAGCCGGGCAGGATAGGTGAAGTAGGCCCACCCGCGAGCGGGTGTTCCTTCTTCACT
GTCCCTTATTCGCACCTGGCGGTGCTCAACGGGAATCCTGCTCTGCGAGGCTGGCCGTAGGCCGGCCCCGTAGAAAAGATCAAAGGATCTTCTTGAGATCCTTTTTTTCTGCGCGTAATCTG
CTGCTTGCAAACAAAAAAACCACCGCTACCAGCGGTGGTTTGTTTGCCGGATCAAGAGCTACCAACTCTTTTTCCGAAGGTAACTGGCTTCAGCAGAGCGCAGATACCAAATACTGTCCTTC
TAGTGTAGCCGTAGTTAGGCCACCACTTCAAGAACTCTGTAGCACCGCCTACATACCTCGCTCTGCTAATCCTGTTACCAGTGGCTGCTGCCAGTGGCGATAAGTCGTGTCTTACCGGGTTG
GACTCAAGACGATAGTTACCGGATAAGGCGCAGCGGTCGGGCTGAACGGGGGGTTCGTGCACACAGCCCAGCTTGGAGCGAACGACCTACACCGAACTGAGATACCTACAGCGTGAGCT
ATGAGAAAGCGCCACGCTTCCCGAAGGGAGAAAGGCGGACAGGTATCCGGTAAGCGGCAGGGTCGGAACAGGAGAGCGCACGAGGGAGCTTCCAGGGGGAAACGCCTGGTATCTTTAT
AGTCCTGTCGGGTTTCGCCACCTCTGACTTGAGCGTCGATTTTTGTGATGCTCGTCAGGGGGGCGGAGCCTATGGAAAAACGCCAGCAACGCGGCCTTTTTACGGTTCCTGGCCTTTTGCTG
GCCTTTTGCTCACATGTTCTTTCCTGCGTTATCCCCTGATTCTGTGGATAACCGTATTACCGCCTTTGAGTGAGCTGATACCGCTCGCCGCAGCCGAACGACCGAGCGCAGCGAGTCAGTGA
GCGAGGAAGCGGAAGAGCGCCTGATGCGGTATTTTCTCCTTACGCATCTGTGCGGTATTTCACACCGCATATGGTGCACTCTCAGTACAATCTGCTCTGATGCCGCATAGTTAAGCCAGTAT
ACACTCCGCTATCGCTACGTGACTGGGTCATGGCTGCGCCCCGACACCCGCCAACACCCGCTGACGCGCCCTGACGGGCTTGTCTGCTCCCGGCATCCGCTTACAGACAAGCTGTGACCGT
CTCCGGGAGCTGCATGTGTCAGAGGTTTTCACCGTCATCACCGAAACGCGCGAGGCAGCTGCGGTAAAGCTCATCAGCGTGGTCGTGCAGCGATTCACAGATGTCTGCCTGTTCATCCGCG
TCCAGCTCGTTGAGTTTCTCCAGAAGCGTTAATGTCTGGCTTCTGATAAAGCGGGCCATGTTAAGGGCGGTTTTTTCCTGTTTGGTCACTGATGCCTCCGTGTAAGGGGGATTTCTGTTCATG
GGGGTAATGATACCGATGAAACGAGAGAGGATGCTCACGATACGGGTTACTGATGATGAACATGCCCGGTTACTGGAACGTTGTGAGGGTAAACAACTGGCGGTATGGATGCGGCGGGG
GCGCGCCCAGCTGTCTAGGGCGGCGGATTTGTCCTACTCAGGAGAGCGTTCACCGACAAACAACAGATAAAACGAAAGGCCCAGTCTTTCGACTGAGCCTTTCGTTTTATTTGATGCC_**363
5**

Complete plasmid sequence of pMiniHisT$_{TAC}$_HisS (nucleotides 1-3834)

mini hisT$_{TAC}$ under control of proK promoter and followed by proK terminator; *hisS* under control of glnS' promoter and RBS

1_GCGGCCGCTTATCCCAGGATGCGCCGGACTTCCGCGACCAGGTCGGCGCGGGCCACCTCGGTCTGGGCGGGCTGGGCCTTCCATTCCTCGTGGCTGGCCTCGGCCGCGATCTTCGCGC
CCAGGACCAGATCCTTGACCTGCACGACGCCGCGCGCCGCCTCGTCCCCGCCCTGGATGATGGCGACGGGGGCATTGCGCTTGTCGGCATATTTCAACTGGTTGCCGAAGTTCTTGGGATT
GCCCAGATAGACCTCGGCCCGGATGCCGGCGGCGCGCAGTTCGGCGGCCATGGCCTGGTAATCGGCCATGCGTTCGCGATCCATGACGGTGACGACGACGGGGCCCTGTTCGGTCCCGC
CCATCAACCCCTTGGCGCGCAGGGCGGCAAGAAGCCGGTCGACGCCGATGGAAACCCCGGTCGCGGGCACCTTCTGGCCGGTGAAGCGTTCCACCAGCCCGTCATAGCGCCCGCCGCCG
GCGACCGAGCCGAACTGGCGCTTGCGGCCTTTGTCGTCGAGGATCTCGAAGGTCAGTTCGGCCTCGAAGACCGGGCCGGTGTAATAGCCCAGGCCCCGCACGATGGAAGGGTCGACGAT
GGCGCGCTCCTCGCCCACGCCCATGGCGGCGAGCATCCGGGCGATCTGCGCCAGTTCCTCGACGCCCTCGGCCCCGGTGGCCGAGGCGCCCACGGCGGCGCGCAGGTTTGCCAGCGTCC
CGGCATTGTCCGCGCCTTTCGAGGTCAGGAAGGCCAGCACCGGCTCGGCCTGTTCGGGGCTCAGGCCCACGCCCTCGATGAAGGCGCCGGATTCGTCCTTGCGGCCGGTGGTCAGCAATT
GCCGCACGCCTTGCTCGCCGACTTTGTCGAACTTGTCGATGGTGCGCAGCACGTCGTCGGCGGGCTTGCCCTCGGCCACGCCCATGGATTCCAGGATGCCGTTCAGCACCTTGCGGTTGTT
GATGCGGATCAGGTAGTCGCCGCGCCGGATGCCGGCATGCTCCAGCGCCGCAGCCAGCATGGCGCAGATCTCGGCATCCGCGGCGACCGAAGCCGAGCCCACGGTATCGGCATCGCATT
GGTAGAACTGGCGGAAGCGGCCGGGGCCGGGCTTTTCGTTGCGCCAGACCGGGCCCATGGCATAGCGGCGATAGGGCGACGGCAGGTCACCCCGGAACTGCGCGGCGACGCGCGCAA
GCGGCGCGGTCAGGTCATAGCGCAACGCCAGCCAGTCGCCGGCCCCGCCGCCGGGAACCTCTGCCTCTTGCCAGGCAAAGACGCCGGCATTGGGGCGGTCCACGTCGGGCAGGAACTTT
CCCAGCGCCTCGACCGTTTCGACGGCACTGGTTTCAAGCGGCTCGAAGCCGTGGCGATGATAGATCTGGGCGATGCGGTCGAGCATCTGCTTGCGCTCGGTCACATCCGCGCCGAAATAG
TCTCGGAACCCCTTGGGCGTCTCGGCCTTGGGACGGGGCTGTTTCTTCTGATCTTTCGCCATTAATTAATTCCTCAAAGCGTAAACAACGTATAACGGCGTATGATATTAAAGCGGGACAGG
CTGACAACTGTTAGTTTTTTAAAATTGATAATCTAACAAGGATTATGGGGATGATTGATGCCTGCAGGAGCGGATAACAATTTCGCGATCGCGGCTAACTAAGCGGCCTGCTGACTTTCTCG
CCGATCAAAAGGCATTTTGCTATTAAGGGATTGACGAGGGCGTATCTGCGCAGTAAGATGCGCCCCGCATTGCCGCCTTAGCGCTAGATT**TAC**GATCTAGAGGTCCCCCGTTCGAGCCGGG
GAGGCGGTACCAAATTCGAAAAGCCTGCTCAACGAGCAGGCTTTTTTGCATGTTTAAACGGGTCCCCAATAATTACGATGGCGAAAATGAGACGTACTGAACCCAATGTCGTTAGTGACGC
TTACCTCTTAAGAGGTCACTGACCTAACATGATCGGCACGTAAGAGGTTCCAACTTTCACCATAATGAAATAAGATCACTACCGGGCGTATTTTTTGAGTTATCGAGATTTTCAGGAGCTAAG
GAAGCTAAAATGGAGAAAAAAATCACTGGATATACCACCGTTGATATATCCCAATGGCATCGTAAAGAACATTTTGAGGCATTTCAGTCAGTTGCTCAATGTACCTATAACCAGACCGTTCA
GCTGGATATTACGGCCTTTTTAAAGACCGTAAAGAAAAATAAGCACAAGTTTTATCCGGCCTTTATTCACATTCTTGCCCGCCTGATGAATGCTCATCCGGAATTTCGTATGGCAATGAAAGA
CGGTGAGCTGGTGATATGGGATAGTGTTCACCCTTGTTACACCGTTTTCCATGAGCAAACTGAAACGTTTTCATCGCTCTGGAGTGAATACCACGACGATTTCCGGCAGTTTCTACACATATA
TTCGCAAGATGTGGCGTGTTACGGTGAAAACCTGGCCTATTTCCCTAAAGGGTTTATTGAGAATATGTTTTTCGTCTCAGCCAATCCCTGGGTGAGTTTCACCAGTTTTGATTTAAACGTGGCC
AATATGGACAACTTCTTCGCCCCCGTTTTCACCATGGGCAAATATTATACGCAAGGCGACAAGGTGCTGATGCCGCTGGCGATTCAGGTTCATCATGCCGTTTGTGATGGCTTCCATGTCGG
CAGAATGCTTAATGAATTACAACAGTACTGCGATGAGTGGCAGGGCGGGGCGTAATTTGACTTTCGCGCGTTGTCCTTTTCCGCTGCATAACCCTGCTTCGGGGTCATTATAGCGATTTTTTC
GGTATATCCATCCTTTTTCGCACGATATACAGGATTTTGCCAAAGGGTTCGTGTAGACTTTCCTTGGTGTATCCAACGGCGTCAGCCGGGCAGGATAGGTGAAGTAGGCCCACCCGCGAGC
GGGTGTTCCTTCTTCACTGTCCCTTATTCGCACCTGGCGGTGCTCAACGGGAATCCTGCTCTGCGAGGCTGGCCGTAGGCCGGCCCTAGAAATATTTTATCTGATTAATAAGATGATCTTCTT
GAGATCGTTTTGGTCTGCGCGTAATCTCTTGCTCTGAAAACGAAAAAACCGCCTTGCAGGGCGGTTTTTCGAAGGTTCTCTGAGCTACCAACTCTTTGAACCGAGGTAACTGGCTTGGAGGA
GCGCAGTCACCAAAACTTGTCCTTTCAGTTTAGCCTTAACCGGCGCATGACTTCAAGACTAACTCCTCTAAATCAATTACCAGTGGCTGCTGCCAGTGGTGCTTTTGCATGTCTTTCCGGGTTG
GACTCAAGACGATAGTTACCGGATAAGGCGCAGCGGTCGGACTGAACGGGGGGTTCGTGCATACAGTCCAGCTTGGAGCGAACTGCCTACCCGGAACTGAGTGTCAGGCGTGGAATGAG
ACAAACGCGGCCATAACAGCGGAATGACACCGGTAAACCGAAAGGCAGGAACAGGAGAGCGCACGAGGGAGCCGCCAGGGGGAAACGCCTGGTATCTTTATAGTCCTGTCGGGTTTCG
CCACCACTGATTTGAGCGTCAGATTTCGTGATGCTTGTCAGGGGGGCGGAGCCTATGGAAAAACGGCTTTGCCGCGGCCCTCTCACTTCCCTGTTAAGTATCTTCCTGGCATCTTCCAGGAA
ATCTCCGCCCCGTTCGTAAGCCATTTCCGCTCGCCGCAGTCGAACGACCGAGCGTAGCGAGTCAGTGAGCGAGGAAGCGGAATATATCCGGCGCGCCCAGCTGTCTAGGGCGGCGGATTT
GTCCTACTCAGGAGAGCGTTCACCGACAAACAACAGATAAAACGAAAGGCCCAGTCTTTCGACTGAGCCTTTCGTTTTATTTGATGCC_**3834**

Complete plasmid sequence of pMiniHisT$_{TAC}$_pur (nucleotides 1-3242)

mini hisT$_{TAC}$ under control of proK promoter and followed by proK terminator

1_GCGATCGCGGCTAACTAAGCGGCCTGCTGACTTTCTCGCCGATCAAAAGGCATTTTGCTATTAAGGGATTGACGAGGGCGTATCTGCGCAGTAAGATGCGCCCCGCATTGCCGCCTTAGC
GCTAGATT**TAC**GATCTAGAGGTCCCCCGTTCGAGCCGGGGAGGCGGTACCAAATTCGAAAAGCCTGCTCAACGAGCAGGCTTTTTTGCATGTTTAAACATCACGTGTGCTATATCCAATGGC
CCTCCCATCCCGTCATGACCAGAAGTAGCGAATCTATTGAGCGACTGAACCCAATGTCACTAGTCTTGGACTCCTGTTGATAGATCCAGTAATGACCTCAGAACTCCATCTGGATTTGTTCAG
AACGCTCGGTTGCCGCCGGGCGTTTTTTATTGGTGAGAATCCAGGGGTCCCCAATAATTACGATTTAAATTAGTAGCCCGCCTAATGAGCGGGCTTTTTTTTAATTCCCCTATTTGTTTATTTTT
CTAAATACATTCAAATATGTATCCGCTCATGAGACAATAACCCTGATAAATGCTTCAATAATATTGAAAAAGGAAGAGTATGAGCATTCAGCATTTTCGTGTGGCGCTGATTCCGTTTTTTGC
GGCGTTTTGCCTGCCGGTGTTTGCGCATCCGGAAACCCTGGTGAAAGTGAAAGATGCGGAAGATCAACTGGGTGCGCGCGTGGGCTATATTGAACTGGATCTGAACAGCGGCAAAATTCT
GGAATCTTTTCGTCCGGAAGAACGTTTTCCGATGATGAGCACCTTTAAAGTGCTGCTGTGCGGTGCGGTTCTGAGCCGTGTGGATGCGGGCCAGGAACAACTGGGCCGTCGTATTCATTATA
GCCAGAACGATCTGGTGGAATATAGCCCGGTGACCGAAAAACATCTGACCGATGGCATGACCGTGCGTGAACTGTGCAGCGCGGCGATTACCATGAGCGATAACACCGCGGCGAACCTG
CTGCTGACGACCATTGGCGGTCCGAAAGAACTGACCGCGTTTCTGCATAACATGGGCGATCATGTGACCCGTCTGGATCGTTGGGAACCGGAACTGAACGAAGCGATTCCGAACGATGAA
CGTGATACCACCATGCCGGCAGCAATGGCGACCACCCTGCGTAAACTGCTGACGGGTGAGCTGCTGACCCTGGCAAGCCGCCAGCAACTGATTGATTGGATGGAAGCGGATAAAGTGGC
GGGTCCGCTGCTGCGTAGCGCGCTGCCGGCTGGCTGGTTTATTGCGGATAAAAGCGGTGCGGGCGAACGTGGCAGCCGTGGCATTATTGCGGCGCTGGGCCCGGATGGTAAACCGAGCC
GTATTGTGGTGATTTATACCACCGGCAGCCAGGCGACGATGGATGAACGTAACCGTCAGATTGCGGAAATTGGCGCGAGCCTGATTAAACATTGGTAAACCGATACAATTAAAGGCTCCTT
TTGGAGCCTTTTTTTTTGGACGACCCTTGTCCTTTTCCGCTGCATAACCCTGCTTCGGGGTCATTATAGCGATTTTTTCGGTATATCCATCCTTTTTCGCACGATATACAGGATTTTGCCAAAGG
GTTCGTGTAGACTTTCCTTGGTGTATCCAACGGCGTCAGCCGGGCAGGATAGGTGAAGTAGGCCCACCCGCGAGCGGGTGTTCCTTCTTCACTGTCCCTTATTCGCACCTGGCGGTGCTCAA
CGGGAATCCTGCTCTGCGAGGCTGGCCGTAGGCCGGCCCCGTAGAAAAGATCAAAGGATCTTCTTGAGATCCTTTTTTTCTGCGCGTAATCTGCTGCTTGCAAACAAAAAAACCACCGCTAC
CAGCGGTGGTTTGTTTGCCGGATCAAGAGCTACCAACTCTTTTTCCGAAGGTAACTGGCTTCAGCAGAGCGCAGATACCAAATACTGTCCTTCTAGTGTAGCCGTAGTTAGGCCACCACTTC
AAGAACTCTGTAGCACCGCCTACATACCTCGCTCTGCTAATCCTGTTACCAGTGGCTGCTGCCAGTGGCGATAAGTCGTGTCTTACCGGGTTGGACTCAAGACGATAGTTACCGGATAAGGC
GCAGCGGTCGGGCTGAACGGGGGGTTCGTGCACACAGCCCAGCTTGGAGCGAACGACCTACACCGAACTGAGATACCTACAGCGTGAGCTATGAGAAAGCGCCACGCTTCCCGAAGGGA
GAAAGGCGGACAGGTATCCGGTAAGCGGCAGGGTCGGAACAGGAGAGCGCACGAGGGAGCTTCCAGGGGGAAACGCCTGGTATCTTTATAGTCCTGTCGGGTTTCGCCACCTCTGACTT
GAGCGTCGATTTTTGTGATGCTCGTCAGGGGGGCGGAGCCTATGGAAAAACGCCAGCAACGCGGCCTTTTTACGGTTCCTGGCCTTTTGCTGGCCTTTTGCTCACATGTTCTTTCCTGCGTTA
TCCCCTGATTCTGTGGATAACCGTATTACCGCCTTTGAGTGAGCTGATACCGCTCGCCGCAGCCGAACGACCGAGCGCAGCGAGTCAGTGAGCGAGGAAGCGGAAGAGCGCCTGATGCGG
TATTTTCTCCTTACGCATCTGTGCGGTATTTCACACCGCATATGGTGCACTCTCAGTACAATCTGCTCTGATGCCGCATAGTTAAGCCAGTATACACTCCGCTATCGCTACGTGACTGGGTCAT
GGCTGCGCCCCGACACCCGCCAACACCCGCTGACGCGCCCTGACGGGCTTGTCTGCTCCCGGCATCCGCTTACAGACAAGCTGTGACCGTCTCCGGGAGCTGCATGTGTCAGAGGTTTTCA
CCGTCATCACCGAAACGCGCGAGGCAGCTGCGGTAAAGCTCATCAGCGTGGTCGTGCAGCGATTCACAGATGTCTGCCTGTTCATCCGCGTCCAGCTCGTTGAGTTTCTCCAGAAGCGTTA
ATGTCTGGCTTCTGATAAAGCGGGCCATGTTAAGGGCGGTTTTTTCCTGTTTGGTCACTGATGCCTCCGTGTAAGGGGGATTTCTGTTCATGGGGGTAATGATACCGATGAAACGAGAGAGG
ATGCTCACGATACGGGTTACTGATGATGAACATGCCCGGTTACTGGAACGTTGTGAGGGTAAACAACTGGCGGTATGGATGCGGCGGGGGCGCGCCCAGCTGTCTAGGGCGGCGGATTTG
TCCTACTCAGGAGAGCGTTCACCGACAAACAACAGATAAAACGAAAGGCCCAGTCTTTCGACTGAGCCTTTCGTTTTATTTGATGCC_**3242**

Complete plasmid sequence of pHisS_tst_pur (nucleotides 1-6797)

hisS with twin-strep-tag under control of T7 promoter and RBS

1_TGGCGAATGGGACGCGCCCTGTAGCGGCGCATTAAGCGCGGCGGGTGTGGTGGTTACGCGCAGCGTGACCGCTACACTTGCCAGCGCCCTAGCGCCCGCTCCTTTCGCTTTCTTCCCTT
CCTTTCTCGCCACGTTCGCCGGCTTTCCCCGTCAAGCTCTAAATCGGGGGCTCCCTTTAGGGTTCCGATTTAGTGCTTTACGGCACCTCGACCCCAAAAAACTTGATTAGGGTGATGGTTCAC
GTAGTGGGCCATCGCCCTGATAGACGGTTTTTCGCCCTTTGACGTTGGAGTCCACGTTCTTTAATAGTGGACTCTTGTTCCAAACTGGAACAACACTCAACCCTATCTCGGTCTATTCTTTTGA
TTTATAAGGGATTTTGCCGATTTCGGCCTATTGGTTAAAAAATGAGCTGATTTAACAAAAATTTAACGCGAATTTTAACAAAATATTAACGCTTACAATTTAGGTGGCACTTTTCGGGGAAATG
TGCGCGGAACCCCTATTTGTTTATTTTTCTAAATACATTCAAATATGTATCCGCTCATGAATTAATTCTTAGAAAAACTCATCGAGCATCAAATGAAACTGCAATTTATTCATATCAGGATTATC

AATACCATATTTTTGAAAAAGCCGTTTCTGTAATGAAGGAGAAAACTCACCGAGGCAGTTCCATAGGATGGCAAGATCCTGGTATCGGTCTGCGATTCCGACTCGTCCAACATCAATACAAC
CTATTAATTTCCCCTCGTCAAAAATAAGGTTATCAAGTGAGAAATCACCATGAGTGACGACTGAATCCGGTGAGAATGGCAAAAGTTTATGCATTTCTTTCCAGACTTGTTCAACAGGCCAGC
CATTACGCTCGTCATCAAAATCACTCGCATCAACCAAACCGTTATTCATTCGTGATTGCGCCTGAGCGAGACGAAATACGCGATCGCTGTTAAAAGGACAATTACAAACAGGAATCGAATGC
AACCGGCGCAGGAACACTGCCAGCGCATCAACAATATTTTCACCTGAATCAGGATATTCTTCTAATACCTGGAATGCTGTTTTCCCGGGGATCGCAGTGGTGAGTAACCATGCATCATCAGG
AGTACGGATAAAATGCTTGATGGTCGGAAGAGGCATAAATTCCGTCAGCCAGTTTAGTCTGACCATCTCATCTGTAACATCATTGGCAACGCTACCTTTGCCATGTTTCAGAAACAACTCTGG
CGCATCGGGCTTCCCATACAATCGATAGATTGTCGCACCTGATTGCCCGACATTATCGCGAGCCCATTTATACCCATATAAATCAGCATCCATGTTGGAATTTAATCGCGGCCTAGAGCAAG
ACGTTTCCCGTTGAATATGGCTCATAACACCCCTTGTATTACTGTTTATGTAAGCAGACAGTTTTATTGTTCATGACCAAAATCCCTTAACGTGAGTTTTCGTTCCACTGAGCGTCAGACCCCG
TAGAAAAGATCAAAGGATCTTCTTGAGATCCTTTTTTTCTGCGCGTAATCTGCTGCTTGCAAACAAAAAAACCACCGCTACCAGCGGTGGTTTGTTTGCCGGATCAAGAGCTACCAACTCTTT
TTCCGAAGGTAACTGGCTTCAGCAGAGCGCAGATACCAAATACTGTCCTTCTAGTGTAGCCGTAGTTAGGCCACCACTTCAAGAACTCTGTAGCACCGCCTACATACCTCGCTCTGCTAATC
CTGTTACCAGTGGCTGCTGCCAGTGGCGATAAGTCGTGTCTTACCGGGTTGGACTCAAGACGATAGTTACCGGATAAGGCGCAGCGGTCGGGCTGAACGGGGGGTTCGTGCACACAGCCC
AGCTTGGAGCGAACGACCTACACCGAACTGAGATACCTACAGCGTGAGCTATGAGAAAGCGCCACGCTTCCCGAAGGGAGAAAGGCGGACAGGTATCCGGTAAGCGGCAGGGTCGGAA
CAGGAGAGCGCACGAGGGAGCTTCCAGGGGGAAACGCCTGGTATCTTTATAGTCCTGTCGGGTTTCGCCACCTCTGACTTGAGCGTCGATTTTTGTGATGCTCGTCAGGGGGGCGGAGCCT
ATGGAAAAACGCCAGCAACGCGGCCTTTTTACGGTTCCTGGCCTTTTGCTGGCCTTTTGCTCACATGTTCTTTCCTGCGTTATCCCCTGATTCTGTGGATAACCGTATTACCGCCTTTGAGTGA
GCTGATACCGCTCGCCGCAGCCGAACGACCGAGCGCAGCGAGTCAGTGAGCGAGGAAGCGGAAGAGCGCCTGATGCGGTATTTTCTCCTTACGCATCTGTGCGGTATTTCACACCGCAAT
GGTGCACTCTCAGTACAATCTGCTCTGATGCCGCATAGTTAAGCCAGTATACACTCCGCTATCGCTACGTGACTGGGTCATGGCTGCGCCCCGACACCCGCCAACACCCGCTGACGCGCCC
TGACGGGCTTGTCTGCTCCCGGCATCCGCTTACAGACAAGCTGTGACCGTCTCCGGGAGCTGCATGTGTCAGAGGTTTTCACCGTCATCACCGAAACGCGCGAGGCAGCTGCGGTAAAGCT
CATCAGCGTGGTCGTGAAGCGATTCACAGATGTCTGCCTGTTCATCCGCGTCCAGCTCGTTGAGTTTCTCCAGAAGCGTTAATGTCTGGCTTCTGATAAAGCGGGCCATGTTAAGGGCGGTT
TTTTCCTGTTTGGTCACTGATGCCTCCGTGTAAGGGGGATTTCTGTTCATGGGGGTAATGATACCGATGAAACGAGAGAGGATGCTCACGATACGGGTTACTGATGATGAACATGCCCGGTT
ACTGGAACGTTGTGAGGGTAAACAACTGGCGGTATGGATGCGGCGGGACCAGAGAAAAATCACTCAGGGTCAATGCCAGCGCTTCGTTAATACAGATGTAGGTGTTCCACAGGGTAGCCA
GCAGCATCCTGCGATGCAGATCCGGAACATAATGGTGCAGGGCGCTGACTTCCGCGTTTCCAGACTTTACGAAACACGGAAACCGAAGACCATTCATGTTGTTGCTCAGGTCGCAGACGTT
TTGCAGCAGCAGTCGCTTCACGTTCGCTCGCGTATCGGTGATTCATTCTGCTAACCAGTAAGGCAACCCCGCCAGCCTAGCCGGGTCCTCAACGACAGGAGCACGATCATGCGCACCCGTG
GGGCCGCCATGCCGGCGATAATGGCCTGCTTCTCGCCGAAACGTTTGGTGGCGGGACCAGTGACGAAGGCTTGAGCGAGGGCGTGCAAGATTCCGAATACCGCAAGCGACAGGCCGATC
ATCGTCGCGCTCCAGCGAAAGCGGTCCTCGCCGAAAATGACCCAGAGCGCTGCCGGCACCTGTCCTACGAGTTGCATGATAAAGAAGACAGTCATAAGTGCGGCGACGATAGTCATGCCC
CGCGCCCACCGGAAGGAGCTGACTGGGTTGAAGGCTCTCAAGGGCATCGGTCGAGATCCCGGTGCCTAATGAGTGAGCTAACTTACATTAATTGCGTTGCGCTCACTGCCCGCTTTCCAGT
CGGGAAACCTGTCGTGCCAGCTGCATTAATGAATCGGCCAACGCGCGGGGAGAGGCGGTTTGCGTATTGGGCGCCAGGGTGGTTTTTCTTTTCACCAGTGAGACGGGCAACAGCTGATTG
CCCTTCACCGCCTGGCCCTGAGAGAGTTGCAGCAAGCGGTCCACGCTGGTTTGCCCCAGCAGGCGAAAATCCTGTTTGATGGTGGTTAACGGCGGGATATAACATGAGCTGTCTTCGGTAT
CGTCGTATCCCACTACCGAGATGTCCGCACCAACGCGCAGCCCGGACTCGGTAATGGCGCGCATTGCGCCCAGCGCCATCTGATCGTTGGCAACCAGCATCGCAGTGGGAACGATGCCCT
CATTCAGCATTTGCATGGTTTGTTGAAAACCGGACATGGCACTCCAGTCGCCTTCCCGTTCCGCTATCGGCTGAATTTGATTGCGAGTGAGATATTTATGCCAGCCAGCCAGACGCAGACGC
GCCGAGACAGAACTTAATGGGCCCGCTAACAGCGCGATTTGCTGGTGACCCAATGCGACCAGATGCTCCACGCCCAGTCGCGTACCGTCTTCATGGGAGAAAATAATACTGTTGATGGGT
GTCTGGTCAGAGACATCAAGAAATAACGCCGGAACATTAGTGCAGGCAGCTTCCACAGCAATGGCATCCTGGTCATCCAGCGGATAGTTAATGATCAGCCCACTGACGCGTTGCGCGAGA
AGATTGTGCACCGCCGCTTTACAGGCTTCGACGCCGCTTCGTTCTACCATCGACACCACCACGCTGGCACCCAGTTGATCGGCGCGAGATTTAATCGCCGCGACAATTTGCGACGGCGCGT
GCAGGGCCAGACTGGAGGTGGCAACGCCAATCAGCAACGACTGTTTGCCCGCCAGTTGTTGTGCCACGCGGTTGGGAATGTAATTCAGCTCCGCCATCGCCGCTTCCACTTTTTCCCGCGT
TTTCGCAGAAACGTGGCTGGCCTGGTTCACCACGCGGGAAACGGTCTGATAAGAGACACCGGCATACTCTGCGACATCGTATAACGTTACTGGTTTCACATTCACCACCCTGAATTGACTCT
CTTCCGGGCGCTATCATGCCATACCGCGAAAGGTTTTGCGCCATTCGATGGTGTCCGGGATCTCGACGCTCTCCCTTATGCGACTCCTGCATTAGGAAGCAGCCCAGTAGTAGGTTGAGGCC
GTTGAGCACCGCCGCCGCAAGGAATGGTGCATGCAAGGAGATGGCGCCCAACAGTCCCCCGGCCACGGGGCCTGCCACCATACCCACGCCGAAACAAGCGCTCATGAGCCCGAAGTGG
CGAGCCCGATCTTCCCCATCGGTGATGTCGGCGATATAGGCGCCAGCAACCGCACCTGTGGCGCCGGTGATGCCGGCCACGATGCGTCCGGCGTAGAGGATCGAGATCGATCTCGATCCC
GCGAAATTAATACGACTCACTATAGGGGAATTGTGAGCGGATAACAATTCCCCTCTAGAAATAATTTTGTTTAACTTTAAGAAGGAGATATACATATGATGGCGAAAGATCAGAAGAAACAG
CCCCGTCCCAAGGCCGAGACGCCCAAGGGGTTCCGAGACTATTTCGGCGCGGATGTGACCGAGCGCAAGCAGATGCTCGACCGCATCGCCCAGATCTATCATCGCCACGGCTTCGAGCCG
CTTGAAACCAGTGCCGTCGAAACGGTCGAGGCGCTGGGAAAGTTCCTGCCCGACGTGGACCGCCCCAATGCCGGCGTCTTTGCCTGGCAAGAGGCAGAGGTTCCCGGCGGCGGGGCCGG
CGACTGGCTGGCGTTGCGCTATGACCTGACCGCGCCGCTTGCGCGCGTCGCCGCGCAGTTCCGGGGTGACCTGCCGTCGCCCTATCGCCGCTATGCCATGGGCCCGGTCTGGCGCAACGA
AAAGCCCGGCCCCGGCCGCTTCCGCCAGTTCTACCAATGCGATGCCGATACCGTGGGCTCGGCTTCGGTCGCCGCGGATGCCGAGATCTGCGCCATGCTGGCTGCGGCGCTGGAGCATGC
CGGCATCCGGCGCGGCGACTACCTGATCCGCATCAACAACCGCAAGGTGCTGAACGGCATCCTGGAATCCATGGGCGTGGCCGAGGGCAAGCCCGCCGACGACGTGCTGCGCACCATCG
ACAAGTTCGACAAAGTCGGCGAGCAAGGCGTGCGGCAATTGCTGACCACCGGCCGCAAGGACGAATCCGGCGCCTTCATCGAGGGCGTGGGCCTGAGCCCCGAACAGGCCGAGCCGGT
GCTGGCCTTCCTGACCTCGAAAGGCGCGGACAATGCCGGGACGCTGGCAAACCTGCGCGCCGCCGTGGGCGCCTCGGCCACCGGGGCCGAGGGCGTCGAGGAACTGGCGCAGATCGCC
CGGATGCTCGCCGCCATGGGCGTGGGCGAGGAGCGCGCCATCGTCGACCCTTCCATCGTGCGGGGCCTGGGCTATTACACCGGCCCGGTCTTCGAGGCCGAACTGACCTTCGAGATCCTC
GACGACAAAGGCCGCAAGCGCCAGTTCGGCTCGGTCGCCGGCGGCGGGCGCTATGACGGGCTGGTGGAACGCTTCACCGGCCAGAAGGTGCCCGCGACCGGGGTTTCCATCGGCGTCG
ACCGGCTTCTTGCCGCCCTGCGCGCCAAGGGGTTGATGGGCGGGACCGAACAGGGCCCCGTCGTCGTCACCGTCATGGATCGCGAACGCATGGCCGATTACCAGGCCATGGCCGCCGAA
CTGCGCGCCGCCGGCATCCGGGCCGAGGTCTATCTGGGCAATCCCAAGAACTTCGGCAACCAGTTGAAATATGCCGACAAGCGCAATGCCCCCGTCGCCATCATCCAGGGCGGGGACGA
GGCGGCGCGCGGCGTCGTGCAGGTCAAGGATCTGGTCCTGGGCGCGAAGATCGCGGCCGAGGCCAGCCACGAGGAATGGAAGGCCCAGCCCGCCCAGACCGAGGTGGCCCGCGCCGA
CCTGGTCGCGGAAGTCCGGCGCATCCTGGGAAGCGCTTGGAGCCATCCACAATTTGAGAAGGGTGGAGGTTCTGGCGGTGGATCGGGAGGTTCAGCGTGGAGCCACCCGCAGTTCGAAA
AAGGCGCCGGATCCTGAGATCCGGCTGCTAACAAAGCCCGAAAGGAAGCTGAGTTGGCTGCTGCCACCGCTGAGCAATAACTAGCATAACCCCTTGGGGCCTCTAAACGGGTCTTGAGGG
GTTTTTTGCTGAAAGGAGGAACTATATCCGGAT_**6797**

Complete plasmid sequence of pDHFRvar (nucleotides 1-2727)

folA variant (all valine codons except **GTA** encoding for V136 were mutated to alanine codons) under control of T7 promoter and RBS

1_GCTAGTGGTGCTAGCCCCGCGAAATTAATACGACTCACTATAGGGTCTAGAAATAATTTTGTTTAACTTTAAGAAGGAGATATACATATGATCAGTCTGATTGCGGCGTTAGCGGCGGATC
GCGCCATCGGCATGGAAAACGCCATGCCGTGGAACCTGCCTGCCGATCTCGCCTGGTTTAAACGCAACACCTTAAATAAACCCGCGATTATGGGCCGCCATACCTGGGAATCAATCGGTCG
TCCGTTGCCAGGACGCAAAAATATTATCCTCAGCAGTCAACCGGGTACGGACGATCGCGCAACGTGGGCAAAGTCGGCTGATGAAGCCATCGCGGCGTGTGGTGACGCGCCAGAAATCAT
GGCCATTGGCGGCGGTCGCGCTTATGAACAGTTCTTGCCAAAAGCGCAAAAACTGTATCTGACGCATATCGACGCAGAAGCTGAAGGCGACACCCATTTCCCGGATTACGAGCCGGATGA
CTGGGAATCG**GTA**TTCAGCGAATTCCACGATGCTGATGCGCAGAACTCTCACAGCTATTGCTTTGAGATTCTGGAGCGGCGGTAATGAGGATCCCGGGAATTCTCGAGTAAGGTTAACCTG
CAGGAGGCCTTTAATTAAGGTGGTGCGGCCGCGCTAGCGGTCCCGGGGGATCGATCCGGCTGCTAACAAAGCCCGAAAGGAAGCTGAGTTGGCTGCTGCCACCGCTGAGCAATAACTAG
CATAACCCCTTGGGGCCTCTAAACGGGTCTTGAGGGGTTTTTTGCTGAAAGGAGGAACTATATCCGGAAGCTTGGCACTGGCCGACCGGGGTCGAGCACTGACTCGCTGCGCTCGGTCGTT
CGGCTGCGGCGAGCGGTATCAGCTCACTCAAAGGCGGTAATACGGTTATCCACAGAATCAGGGGATAACGCAGGAAAGAACATGTGAGCAAAAGGCCAGCAAAAGGCCAGGAACCGTA
AAAAGGCCGCGTTGCTGGCGTTTTTCCATAGGCTCCGCCCCCCTGACGAGCATCACAAAAATCGACGCTCAAGTCAGAGGTGGCGAAACCCGACAGGACTATAAAGATACCAGGCGTTTC
CCCCTGGAAGCTCCCTCGTGCGCTCTCCTGTTCCGACCCTGCCGCTTACCGGATACCTGTCCGCCTTTCTCCCTTCGGGAAGCGTGGCGCTTTCTCATAGCTCACGCTGTAGGTATCTCAGTT
CGGTGTAGGTCGTTCGCTCCAAGCTGGGCTGTGTGCACGAACCCCCCGTTCAGCCCGACCGCTGCGCCTTATCCGGTAACTATCGTCTTGAGTCCAACCCGCTAAGACACGACTTATCGCCA
CTGGCAGCAGCCACTGGTAACAGGATTAGCAGAGCGAGGTATGTAGGCGGTGCTACAGAGTTCTTGAAGTGGTGGCCTAACTACGGCTACACTAGAAGAACAGTATTTGGTATCTGCGCTC
TGCTGAAGCCAGTTACCTTCGGAAAAAGAGTTGGTAGCTCTTGATCCGGCAAACAAACCACCGCTGGTAGCGGTGGTTTTTTTGTTTGCAAGCAGCAGATTACGCGCAGAAAAAAAGGATC
TCAAGAAGATCCTTTGATCTTTTCTACGGGGTCTGACGCTCAGTGGAACGAAAACTCACAGATCCGGGATTTTGGTCATGAGATTATCAAAAAGGATCTTCACCTAGATCCTTTTAAATTAAA
AATGAAGTTTTAAATCAATCTAAAGTATATATGAGTAAACTTGGTCTGACAGTTACCAATGCTTAATCAGTGAGGCACCTATCTCAGCGATCTGTCTATTTCGTTCATCCATAGTTGCCTGACT
CCCCGTCGTGTAGATAACTACGATACGGGAGGGCTTACCATCTGGCCCCAGTGCTGCAATGATACCGCGAGACCCACGCTCACCGGCTCCAGATTTATCAGCAATAAACCAGCCAGCCGG
AAGGGCCGAGCGCAGAAGTGGTCCTGCAACTTTATCCGCCTCCATCCAGTCTATTAATTGTTGCCGGGAAGCTAGAGTAAGTAGTTCGCCAGTTAATAGTTTGCGCAACGTTGTTGCCATTG
CTACAGGCATCGTGGTGTCACGCTCGTCGTTTGGTATGGCTTCATTCAGCTCCGGTTCCCAACGATCAAGGCGAGTTACATGATCCCCCATGTTGTGCAAAAAAGCGGTTAGCTCCTTCGGT
CCTCCGATCGTTGTCAGAAGTAAGTTGGCCGCAGTGTTATCACTCATGGTTATGGCAGCACTGCATAATTCTCTTACTGTCATGCCATCCGTAAGATGCTTTTCTGTGACTGGTGAGTACTCAA
CCAAGTCATTCTGAGAATAGTGTATGCGGCGACCGAGTTGCTCTTGCCCGGCGTCAATACGGGATAATACCGCGCCACATAGCAGAACTTTAAAAGTGCTCATCATTGGAAAACGTTCTTCG
GGGCGAAAACTCTCAAGGATCTTACCGCTGTTGAGATCCAGTTCGATGTAACCCACTCGTGCACCCAACTGATCTTCAGCATCTTTTACTTTCACCAGCGTTTCTGGGTGAGCAAAAACAGG
AAGGCAAAATGCCGCAAAAAAGGGAATAAGGGCGACACGGAAATGTTGAATACTCATACTCTTCCTTTTTCAATATTATTGAAGCATTTATCAGGGTTATTGTCTCATGAGCGGATACATATT
TGAATGTATTTAGAAAAATAAACAAATAGGGGTTCCGCGCACATTTCCCCGAAAAGT_**2727**

References

1. Martínez-García, E., Fraile, S., Algar, E., Aparicio, T., Velázquez, E., Calles, B., Tas, H., Blázquez, B., Martín, B., Prieto, C. *et al.* (2023) SEVA 4.0: an update of the Standard European Vector Architecture database for advanced analysis and programming of bacterial phenotypes. *Nucleic Acids Res*, **51**, D1558-D1567.
2. Endy, D. (2005) Foundations for engineering biology. *Nature*, **438**, 449-453.
3. Borujeni, A.E., Channarasappa, A.S. and Salis, H.M. (2014) Translation rate is controlled by coupled trade-offs between site accessibility, selective RNA unfolding and sliding at upstream standby sites. *Nucleic Acids Res*, **42**, 2646-2659.
4. Ryu, Y.H. and Schultz, P.G. (2006) Efficient incorporation of unnatural amino acids into proteins in. *Nat Methods*, **3**, 263-265.
5. Datsenko, K.A. and Wanner, B.L. (2000) One-step inactivation of chromosomal genes in Escherichia Coli K-12 using PCR products. *P Natl Acad Sci USA*, **97**, 6640-6645.
6. Cherepanov, P.P. and Wackernagel, W. (1995) Gene Disruption in Escherichia-Coli - Tcr and Km(R) Cassettes with the Option of Flp-Catalyzed Excision of the Antibiotic-Resistance Determinant. *Gene*, **158**, 9-14.
